# Supplementary material for: Somatic mutations of CADM1 in aldosterone-producing adenomas and gap junction-dependent regulation of aldosterone production
Source: Nat Genet. 2023 Jun 8;55(6):1009–21. doi: 10.1038/s41588-023-01403-0 (PMC10260400; doi:10.1038/s41588-023-01403-0)
Supplement: Supplementary file 1 — Supplementary Figs. 1–11, Supplementary Tables 1–16, supporting data for Supplementary Figs. 2a–c and 8c,f (uncropped scans of blots) and Supplementary Note [file 41588_2023_1403_MOESM1_ESM.pdf]

nature genetics

Article https://doi.org/10.1038/s41588-023-01403-0

Somatic mutations of *CADM1* in aldosterone-producing adenomas and gap junction-dependent regulation of aldosterone production

In the format provided by the authors and unedited

Supplementary Fig. 1
CADM1 somatic mutations found in APAs affect protein tertiary structure

a. Primer set specific for targeted regions in the cDNA of CADM1.
Different primer sets were used for RT-PCR of CADM1 in human adrenal cells that could differentiate between alternatively spliced transcripts' expression.

***b. CADM1* isoform expression in adjacent normal adrenals (N) and APAs (T).**
RT-PCR for paired adjacent normal adrenal (N) and APA (T) cDNA samples were performed using primer set A. Isoform encoding CADM1 of 442aa length (244bp) was found to be the most predominant of all the membrane localised CADM1 isoforms in N and T samples from patients with different somatic mutation status (as indicated in the figure).

***c. CADM1* isoform expression in the human adrenocortical cell line H295R.**
Primer set A verified that expression pattern of CADM1 isoforms in H295R cells follow that in primary adrenal cells. This expression pattern was confirmed by another pair of primers, primer set C with the shorter product found to be predominant, i.e. predominantly of 442aa length. The isoform with 453 aa length was also found to be present, though less abundant. In addition to these two membrane localised isoforms of CADM1, positive amplification with primer set C confirmed the expression of soluble/secreted isoform of CADM1.

Supplementary Fig. 1

CADM1 expression in in human adrenal cells

a. Primer set specific for targeted regions in the cDNA of CADM1

Primer set	Forward Primer	Reverse Primer	Expected size (bp) (442aa/453aa/471aa)	Expected size (bp) (for soluble isoform)
A	GCTGTATGTATACG ATCCCCCCAC	AGTGAAGTATGTACC TTTATGTCTGGC	244/277/331	N/A
B	GCTTGAGTTAACA TGTGAAGCCATCG	CCAACTGGTTTTTGA TTTTCAACTTGACC	N/A	317
C	GCTTGAGTTAACA TGTGAAGCCATCG	AGTGAAGTATGTACC TTTATGTCTGGC	439/472/526	N/A

***b.* *CADM1* isoform expression in adjacent normal adrenals (N) and APAs (T)**

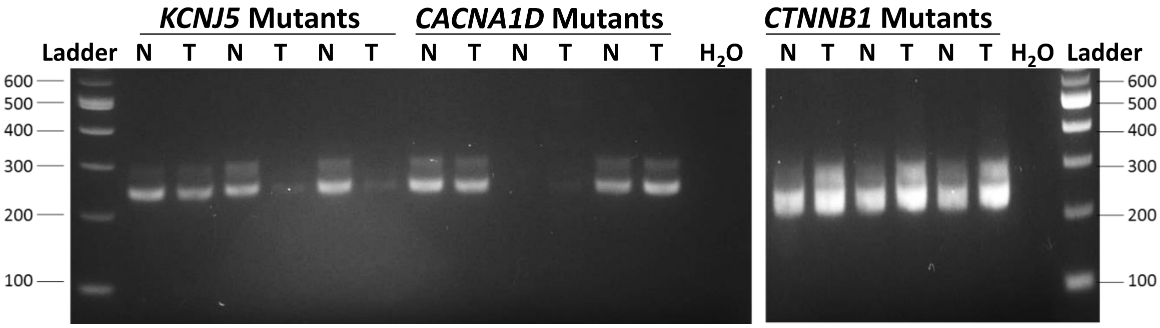

***c.* *CADM1* isoform expression in the human adrenocortical cell line H295R**

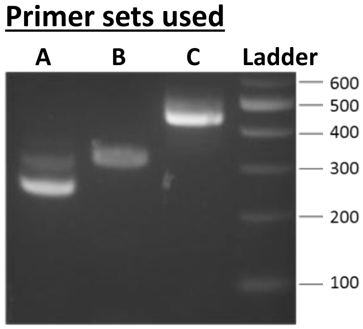

Supplementary Fig. 2
***CADM1* somatic mutations found in APAs affect protein tertiary structure**

a. **Western Blot of Mouse NIH 3T3 embryo fibroblast cells transduced with either wild-type (WT) or mutant *CADM1* (G379D or V380D) in a pLOC vector.**
Top immunoblot show cell lysates stained using the same C-terminal CADM1 antibody used in (a). NIH 3T3 cells does not express native CADM1 as confirmed by appearance of only non-specific bands on immunoblot of untransduced (UT) cell lysates. Cell lysate of WT CADM1 (442aa isoform) stably transfected cells (442aa WT stable) was used as a positive control. Experiment repeated twice independently with similar results. Bottom immunoblot show the medium supernatant from same cells stained using an N-terminal CADM1 antibody (#CM004-3, MBL, Japan) to detect shed CADM1 N-terminal fragments (NTF). Experiment repeated once independently with similar results.

b. **Western Blot of H295R cells transfected with wild-type (WT) or mutant *CADM1* (G379D and V380D) in a pCX4bsr vector after short exposure (*Left immunoblot*) or long exposure (Right immunoblot).**
The effect of mutant *CADM1* on the short isoform (442aa) and long isoform (453aa) of CADM1 were investigated. Cell lysates were immunoblotted with a custom-made anti-CADM1 C-terminal antibody. Experiment repeated twice independently with similar results. Zoomed image of short exposure of glycosylated full length CADM1 bands, long exposure of α-CTF bands, and β-actin immunoblot of same cell lysates are shown in Figure 3a. UT, untransfected.

c. **Western Blot of H295R cells transduced with wild-type (WT) or mutant *CADM1* (G379D or V380D) in a pLOC vector.**
Top immunoblot show cell lysates stained using a commercial C-terminal CADM1 antibody (S4945, Sigma-Aldrich, USA). Cell lysates of untransduced (UT) cells or cells transduced with the empty vector (EV) were used to differentiate from native CADM1 expressed in H295R cells. To note, a longer exposure of regions between 15-20 kDa was needed to visualize the CTF bands. Experiment repeated once independently with similar results. Middle immunoblot show the same cell lysates stained using the anti-GAPDH antibody to estimate total protein loaded in each well. Bottom immunoblot show the medium supernatant of same cells stained using the same N-terminal CADM1 antibody used in Supplementary Fig. 2a to detect shed CADM1 N-terminal fragments (NTF) .

d. **Protein modelling data predicts reduced length of the transmembrane (TM) helix in mutant CADM1.**
Top panel, representative schematic diagrams depicting reduction in length of the TM helix (red double-headed arrow) from 33A in wild-type (WT) to 28.35A and 25.07A in G379D and V380D mutants respectively as predicted using QUARK[96]. This leads to an overall reduction in length of the TM domain (black double-headed arrow) from 33A (WT) to 31.74A (G379D) and 31.42A (V380D).
Bottom panel, representative schematic diagrams depicting increase in tilt angle of the TM helix within the lipid bilayer from 49.4° in wild-type to 62.1° and 90° in the G379D and V380D CADM1 mutants respectively. In dimers of wild-type and mutant CADM1, the shorter length of the TM domain in the mutant is expected to make the tilt angle nearer to 90° than 49.4°.

e. **Schematic of predicted homodimerization of mutant CADM1 TM domain.**
Lateral view of CADM1 TM domain inserted in the lipid bilayer (presented as yellow spheres). The introduction of a charged, unpaired aspartic acid residue (379D and 380D) in the hydrophobic lipid bilayer is likely to affect the tilt angle of the TM domain, and possibly CADM1 TM dimerization, thus leading to a change in the exit angle of the extracellular stalk. The proteins structure of the TM domain for this schematic diagram was modelled using Phyre[105] (for mutants) and TMDOCK[106] (for wild-type) to predict the insertion and homodimerization of the TM region.

Supplementary Fig. 2
***CADM1* somatic mutations found in APAs affect protein tertiary structure**

a. Western Blot of Mouse NIH 3T3 embryo fibroblast cells transduced with either wild-type (WT) or mutant *CADM1* (G379D or V380D) in a pLOC vector.

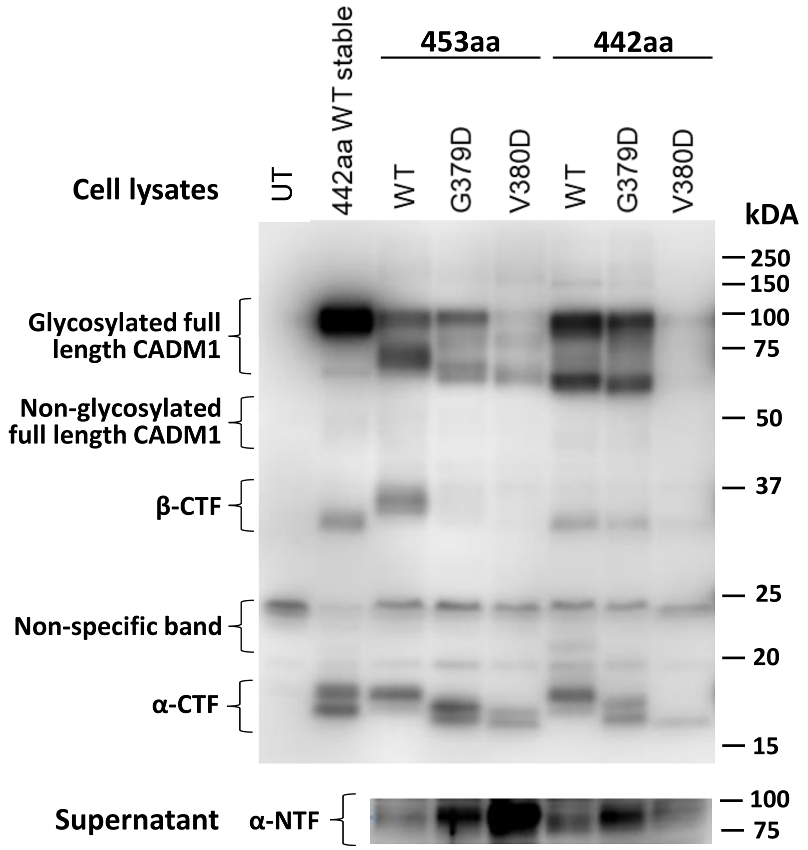

b. Western Blot of H295R cells transfected with wild-type (WT) or mutant *CADM1* (G379D and V380D) in a pCX4bsr vector after short exposure (Left immunoblot) or long exposure (Right immunoblot).

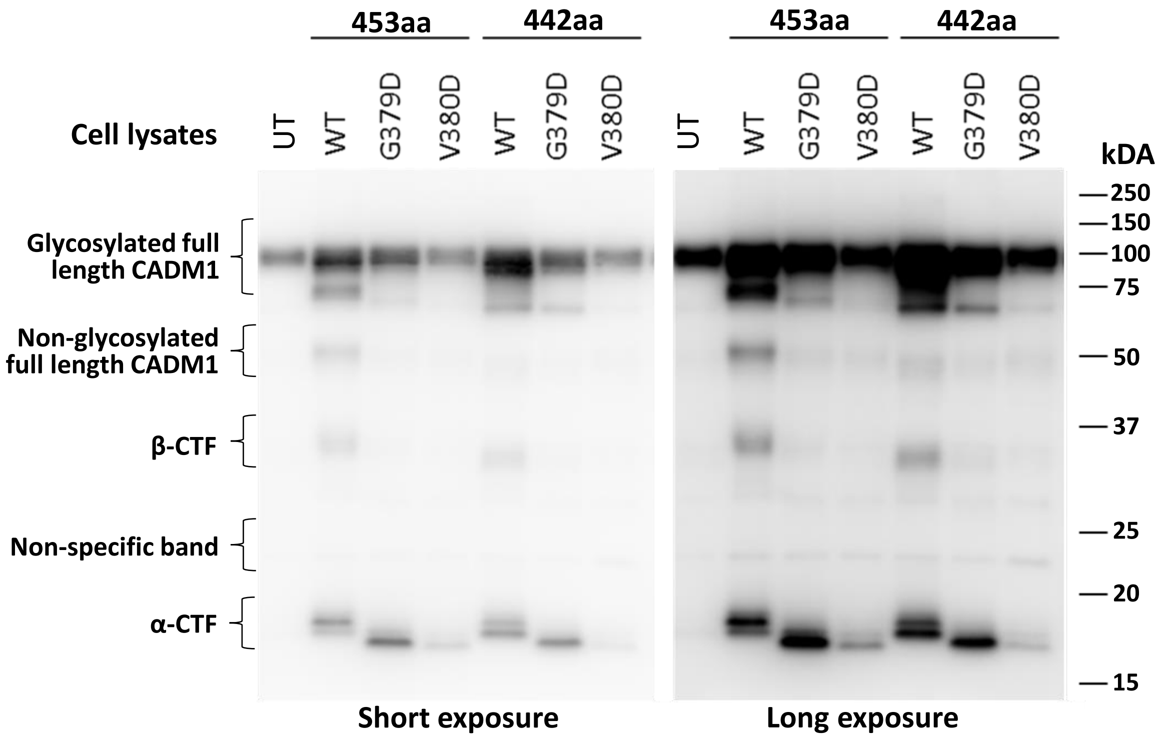

Supplementary Fig. 2

CADM1 somatic mutations found in APAs affect protein tertiary structure

c. **Western Blot of H295R cells transduced with wild-type (WT) or mutant *CADM1* (G379D or V380D) in a pLOC vector.**

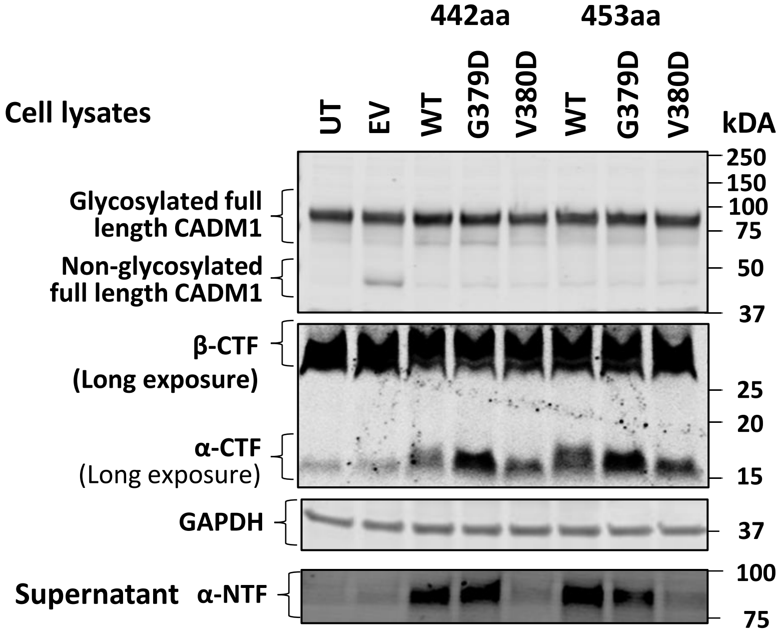

d. **Protein modelling data predicts reduced length of the transmembrane (TM) helix in mutant CADM1.**

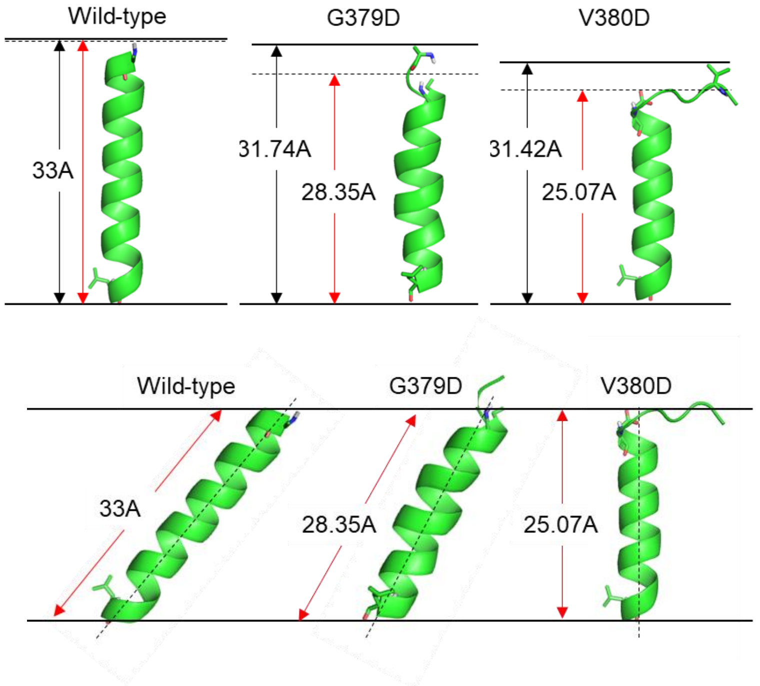

e. **Schematic of predicted homodimerization of mutant CADM1 TM domain.**

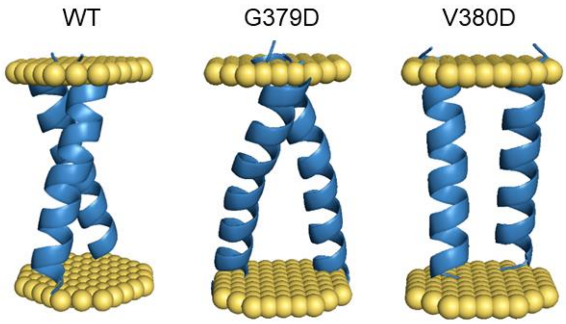

Supplementary Fig. 3
Modulation of CADM1 affects gap junction (GJ) communication

a. **Representative images of H295R cells transfected with 442aa wild-type *CADM1*-GFP or mutant (V380D) *CADM1*-GFP at start of dye transfer assay (0h).**
Dye transfer assays was performed in single GFP-positive (transfected) H295R cells injected with the GJ-impermeable WGA dye (orange) and the GJ-permeable Calcein-Red dye (red). Images of cells were taken immediately (0 h) and 1 h post dye injection. Representative images of cells at end of dye transfer assay are shown in Fig. 4a. Image representative of 11 independent experiments.

b. **Representative images of H295R cells transfected with non-targeting shRNA-GFP or sh*CADM1*-GFP at start of dye transfer assay (0h).**
Dye transfer assays was performed as in Supplementary Fig. 3a. Images of cells shown were taken immediately post dye injection (0h). Representative images of cells at end of dye transfer assay (1h) are shown in Supplementary Fig. 3c. Image representative of 13 cells transfected with non-targeting shRNA-GFP and 17 cells transfected with shCADM1-GFP.

c. **Representative images of H295R cells transfected with non-targeting shRNA-GFP or sh*CADM1*-GFP at end of dye transfer assay (1 h).**
H295R cells transfected with sh*CADM1*-GFP (green) had diminished transfer of GJ-permeable Calcein-Red dye (red) 1 h post dye injection in adjacent cells compared to cells transfected with non-targeting shRNA suggesting silencing *CADM1* decrease GJ intercellular communication. Quantification of dye transfer assay is shown in Fig. 4b. Image representative of 13 cells transfected with non-targeting shRNA-GFP and 17 cells transfected with shCADM1-GFP.

d. **Representative images of H295R cells cells treated with Fc-control or with soluble CADM1 at start of dye transfer assay (0 h) using GJ impermeable dye Dil (red) and GJ permeable dye BCECF (green).**
Dye transfer assays was performed in single H295R cells injected with a gap junction impermeable dye Dil (red) and gap junction permeable dye BCECF (green) pre-treated with Fc-control (10 µg/ml) or soluble CADM1 (10 µg/ml) . The soluble CADM1 is the secreted form of CADM1 fused to the fragment crystallizable (Fc) region of immunoglobulin IgG whereas the Fc-control is the Fc region of IgG without the 3 immunoglobulin ectodomains of CADM1. Treatment was performed 24 hours prior to dye injection. Images of cells shown were taken immediately post dye injection (0h). Representative images of cells at end of dye transfer assay 1 h post dye-injection are shown in Supplementary Fig. 3e. Image representative of 5 independent experiments.

e. **Representative images of H295R cells cells treated with Fc-control or with soluble CADM1 at end of dye transfer assay (1h) using GJ impermeable dye Dil (red) and GJ permeable dye BCECF (green).**
H295R cells treated with soluble CADM1 had diminished transfer of GJ permeable dye BCECF (green) 1 h post dye injection in adjacent cells compared to cells treated with Fc-control suggesting shed CADM1 can decrease GJ intercellular communication. Image representative of 5 independent experiments.

f. **Representative images of recent GJ communication (yellow arrow) in a H295R cell co-transfected with *CADM1*-GFP and *GJA1*-mApple co-cultured with H295R cells transfected with *GJA1*-Venus only.**
To quantify directly the effect of *CADM1* mutation on GJ communication, H295R cells were co-transfected with *GJA1*-mApple (red) and either WT or mutant *CADM1*-pLOC vectors (GFP-tagged). Hoechst was used to identify cell nucleus. These cells were plated with cells transfected with *GJA1*-Venus (represented in yellow) only. Double transfected cells (GFP positive and mApple positive) were imaged using confocal microscopy. Recent GJ communication is estimated by presence of GJA1-Venus GJ plaque (yellow arrow) in a double transfected cell.

White scale bar = 20 µm.

Supplementary Fig. 3
Modulation of CADM1 affects gap junction (GJ) communication

a. Representative images of H295R cells transfected with 442aa wild-type *CADM1*-GFP or mutant (V380D) *CADM1*-GFP at start of dye transfer assay (0h).

Wild-Type *CADM1*-GFP (0h)

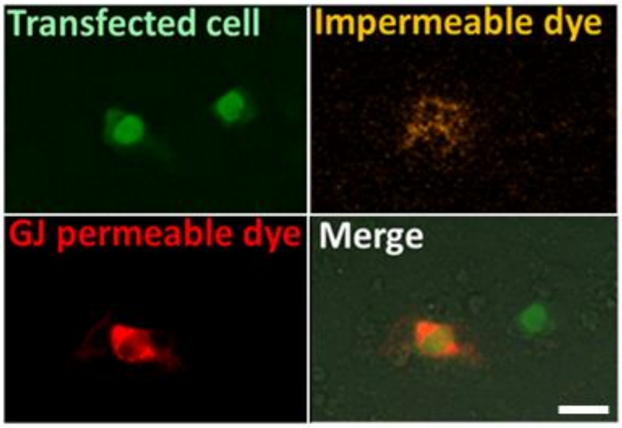

Mutant *CADM1*-GFP (0h)

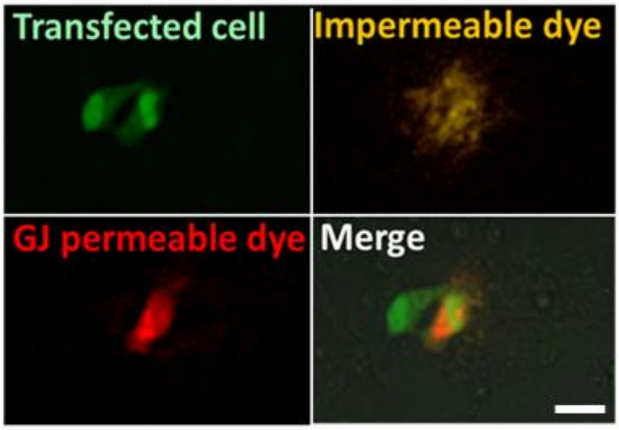

b. Representative images of H295R cells transfected with non-targeting shRNA-GFP or shCADM1-GFP at start of dye transfer assay (0h).

Non-targeting shRNA-GFP (0h)

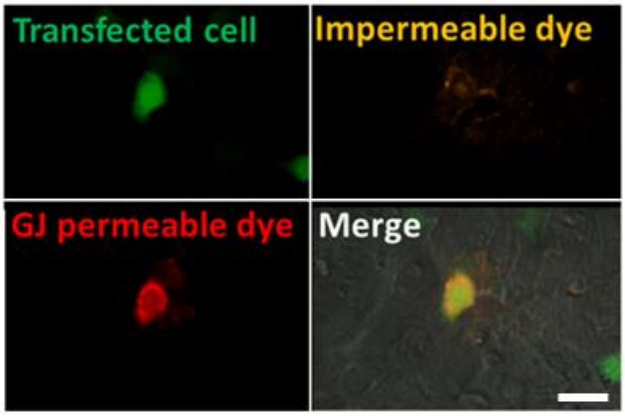

sh*CADM1*-GFP (0h)

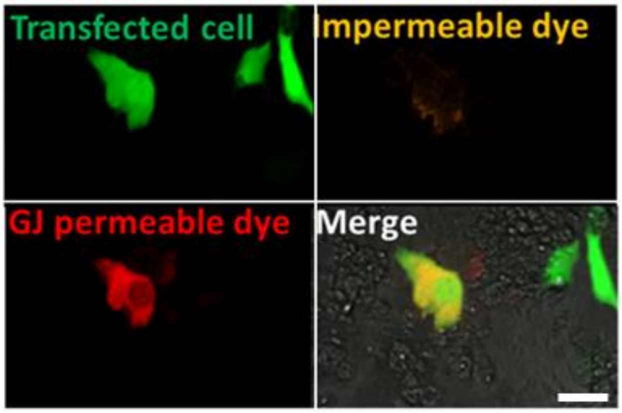

c. Representative images of H295R cells transfected with non-targeting shRNA-GFP or sh*CADM1*-GFP at end of dye transfer assay (1 h).

Non-targeting shRNA-GFP (1h)

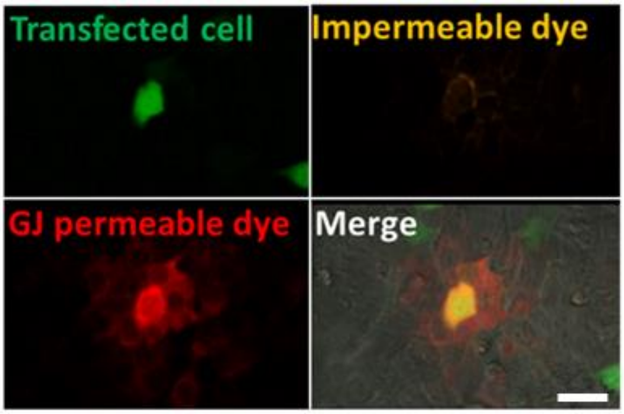

sh*CADM1*-GFP (1h)

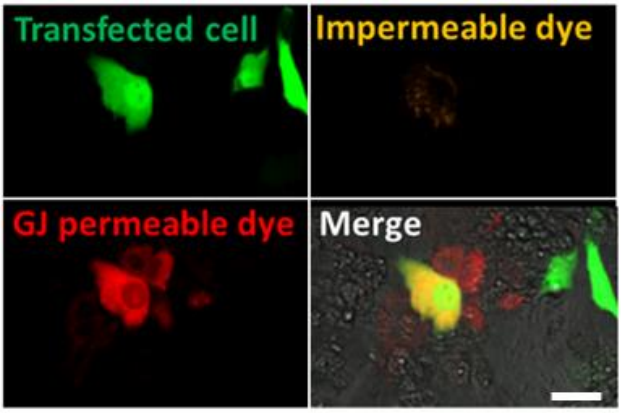

Supplementary Fig. 3
Modulation of CADM1 affects gap junction (GJ) communication

d. **Representative images of H295R cells cells treated with Fc-control or with soluble CADM1 at start of dye transfer assay (0h) using GJ impermeable dye Dil (red) and GJ permeable dye BCECF (green).**

Fc-control (0h)

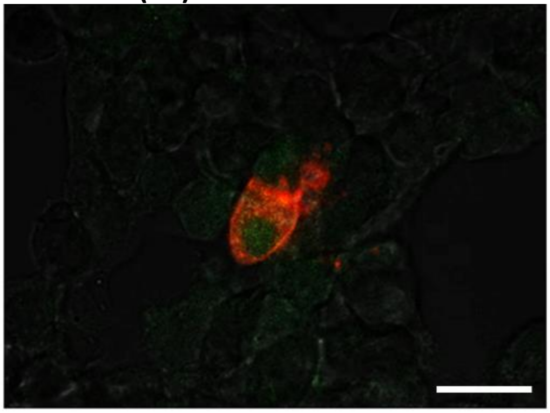

Soluble CADM1 (0h)

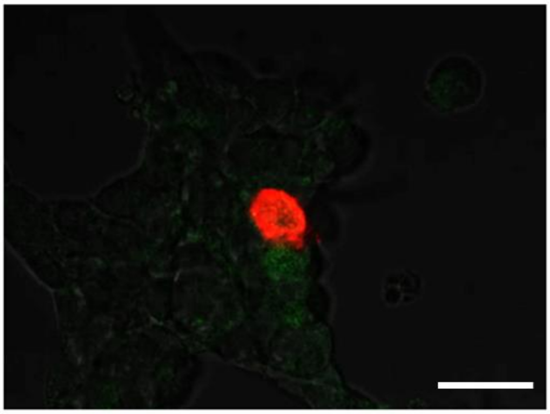

e. **Representative images of H295R cells cells treated with Fc-control or with soluble CADM1 (at end of dye transfer assay (1 h) using GJ impermeable dye Dil (red) and GJ permeable dye BCECF (green).**

Fc-control (1h)

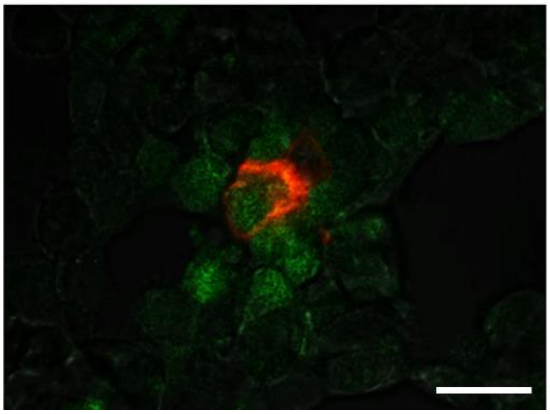

Soluble CADM1 (1h)

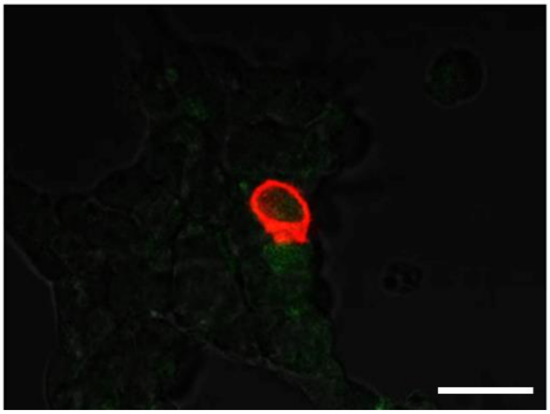

f. **Representative images of recent GJ communication (yellow arrow) in a H295R cell co-transfected with *CADM1*-GFP and *GJA1*-mApple co-cultured with H295R cells transfected with *GJA1*-Venus only.**

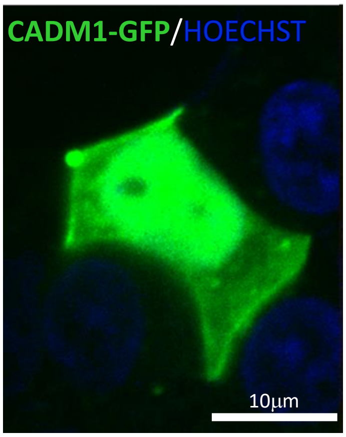

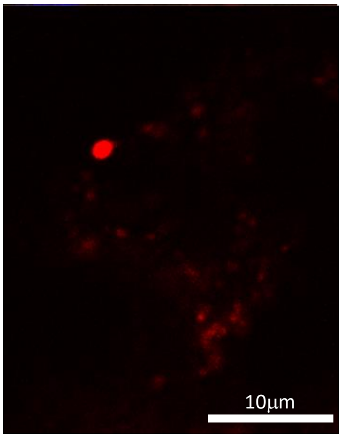

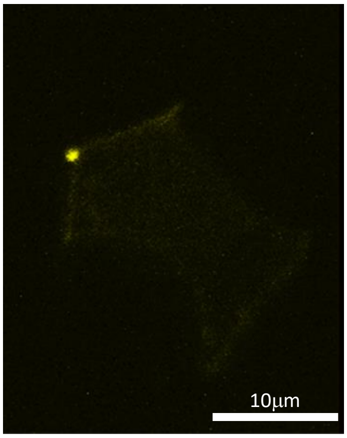

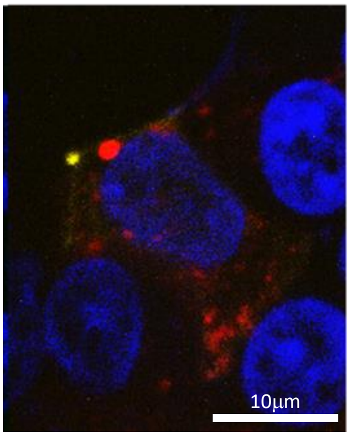

Supplementary Fig. 4
Differential expression of *GJA1* mRNA in human adrenal cortex

a. mRNA expression of the ZG marker *LGR5* in LCM samples of ZF and ZG as detected by microarray (*Left panel*) and as validated by qPCR (*Right panel*).

Cresyl Violet staining followed by LCM sampling allowed selective acquisition of the different adrenal zones as depicted **in Extended Data Fig. 3a from** the adjacent adrenal gland (AAG) of 7 *KCNJ5*-mutant APAs, 7 ZG-like APAs, and 7 phaeochromocytomas (Phaeo). Validation of microarray results by qPCR was performed in triplicates from 3 independent samples. Selective sampling of ZG cells is shown by the high expression of *LGR5*. The hepatocellular carcinoma cell line HepG2 was used as a positive control as this cell line expresses high levels of *LGR5*. For microarray data $^{\#\#\#}p<0.0001$. For qPCR data Adrenal 1:***$p=0.0017$, Adrenal 2 *$p=0.0093$, ****$p<0.0001$

b. mRNA expression of the ZF marker *GSTA3* in LCM samples of ZF and ZG as detected by microarray (*Left panel*) and as validated by qPCR (Rig*ht panel*).

Same samples used in (a) were analyzed for the ZF marker *GSTA3*. Selective sampling of ZF cells is shown by the high expression of *GSTA3* mRNA, whereas selective sampling of ZG cells is verified by the low expression of *GSTA3* mRNA. The HepG2 cell line was used as a negative control as it does not express *GSTA3*. For microarray data $^{\#\#\#}p<0.0001$. For qPCR data Adrenal 1:***$p=0.0007$, Adrenal 2 **$p=0.0076$, ****$p<0.0001$

***c.* *GJA1* mRNA expression in LCM samples of ZF and ZG as detected by microarray (Left panel) and as validated by qPCR (Right panel).**

Same samples used in (a-b) were analysed for *GJA1*. *GJA1* was highly expressed in ZF samples, with a lower expression detected in ZG. No expression of *GJA1* was not detected in the HepG2 cell line. For microarray data $^{\#\#\#}p<0.0001$. For qPCR data Adrenal 1:***$p=0.0009$, Adrenal 2 *$p=0.0206$, ****$p<0.0001$

Data are presented as mean values +/- s.e.m. Statistical analysis was performed using two-sided Student's T-test. For microarray data, comparison made with ZF. For qPCR data, comparison made with ZF of corresponding adrenal.

Supplementary Fig. 4
Differential expression of GJA1 mRNA in human adrenal cortex

a. mRNA expression of the ZG marker LGR5 in LCM samples of ZF and ZG as detected by microarray (Left panel) and as validated by qPCR (Right panel).

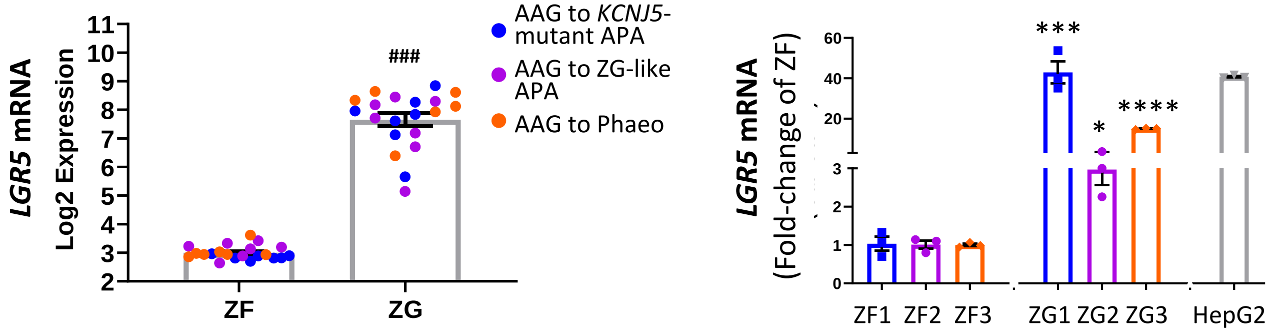

b. mRNA expression of the ZF marker GSTA3 in LCM samples of ZF and ZG as detected by microarray (Left panel) and as validated by qPCR (Right panel).

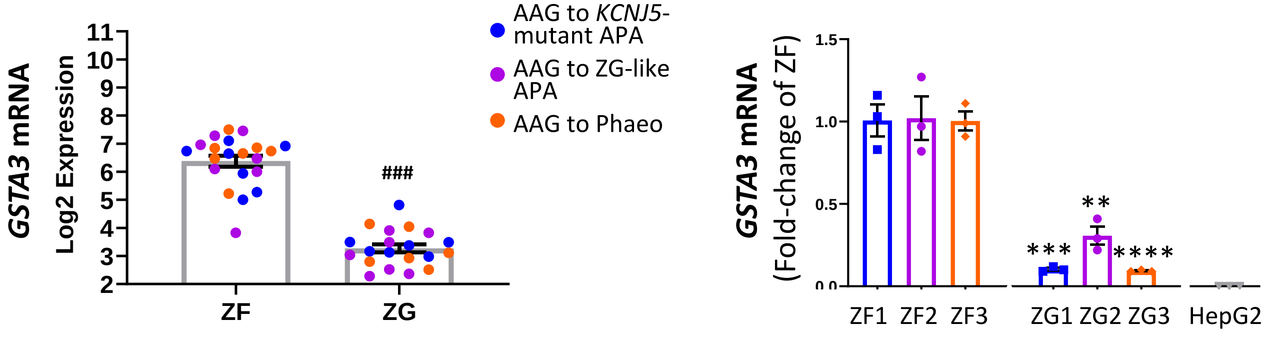

c. GJA1 mRNA expression in LCM samples of ZF and ZG as detected by microarray (Left panel) and as validated by qPCR (Right panel).

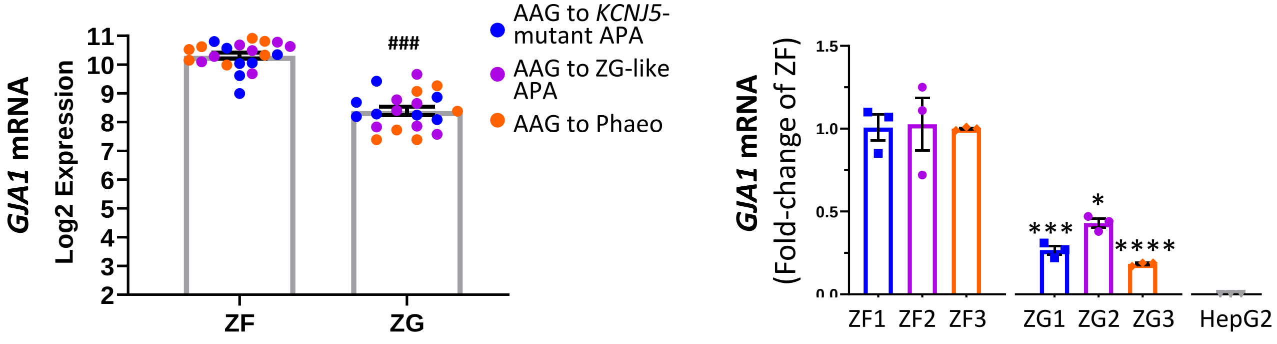

Supplementary Fig. 5
Variable expression of GJA1 protein in human adrenal cortex

a. **Immunostaining of positive control tissues for antibodies used to determine zonation of adrenals.** Expected positive staining of TJP1, CADM1, CYP17A1, KCNJ5 and CYP11B2 in human kidney, testis, and adrenal as indicated. *Left* panels, low magnification; *Right* panels, high magnification. Dashed lines demarcate the APA in the adrenal sections.

b. **Selective immunostaining of GJA1 is detected using two different primary antibodies.** Antigen peptide control for GJA1 (APREST87105, Sigma-Aldrich, USA) competed with anti-GJA1 antibody in a dose response manner in 2 positive control tissues, heart (*Top* panel) and testis (*Middle* panel). To note, the two different primary antibodies that were used had similar positive staining in serial testis sections. The no primary (1°) antibody control staining validates the GJA1 positive staining seen by IFC is not autofluorescence (*Bottom* panel). The specific GJA1 staining is present in compact cells with elongated nucleus (visualized using DAPI) adjacent to the capsule (C). These cells are assumed to be ZG cells based on cell histology and morphology.

c. **Presence of GJA1 protein expression in the ZG of the excised adrenal from the index patient P1 and a control adrenal.** GJA1 expression in ZG was lower than in ZF in the index patient P1 and control adrenal 1 from a patient with a neuroendocrine tumour. However, valid serial controls [two different primary antibodies – GJA1(HPA) and GJA1 (C6219), no primary antibody control (No 1° Ab), antigen peptide for GJA1 control, and relevant ZG and ZF markers (KCNJ5 and CYP17A1 respectively)] confirm expression of GJA1 in human ZG. *Left* panels, low magnifications; Right panels, high magnifications of colour coded boxed regions.

d. **IFC for CYP17A1 (ZF marker), DAB2 (ZG marker), and GJA1 in serial adrenal sections from 2 adrenals.** IFC of a ZF marker, ZG marker, and GJA1 was performed in 2 adrenals, one excised from a 56 years-old PA patient and the other from a 59 years-old PA patient. IFC stainings are colour coded as indicated on each image. A no primary (1°) antibody control confirmed autofluorescence in this region was limited to red blood cells (white arrows). GJA1 expression in ZG regions (DAB2 positive regions) was present although lower compared to ZF regions (CYP17A1 positive regions).

e. **High magnification of IFC for CYP17A1 (ZF marker) and GJA1 identifies AGJ.** Zoomed images of adrenal sections presented in d identifies AGJ (yellow arrows) in both CYP17A1 negative (-ve) and CYP17A1 positive (+ve) regions suggesting presence of gap junctions in both ZG and ZF.

Individual scale bars are shown with each image.

Supplementary Fig. 5
Variable expression of GJA1 protein in human adrenal cortex

a. Immunostaining of positive control tissues for antibodies used to determine zonation of adrenals.

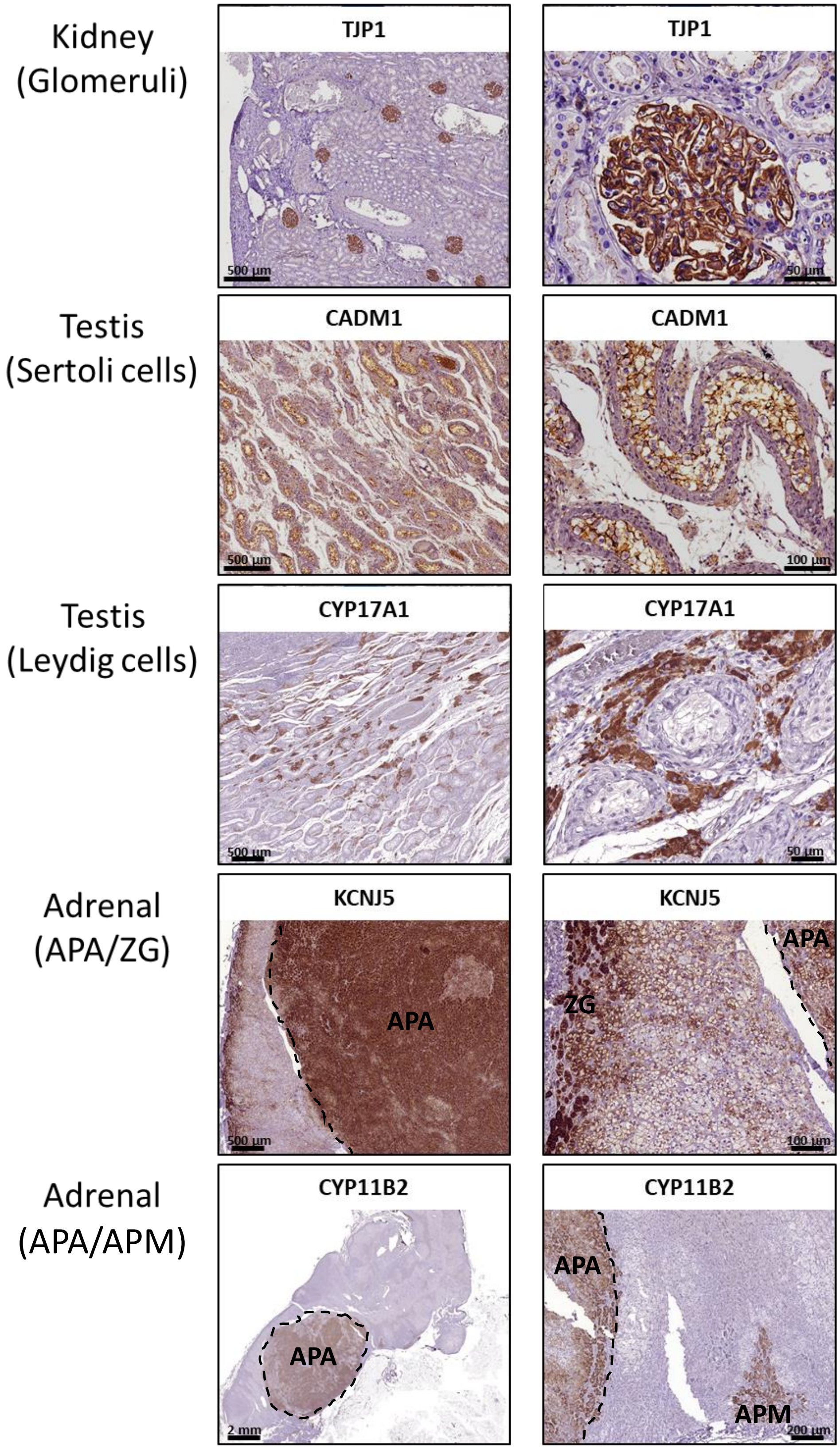

Supplementary Fig. 5
Variable expression of GJA1 protein in human adrenal cortex

b. Selective immunostaining of GJA1 is detected using two different primary antibodies.

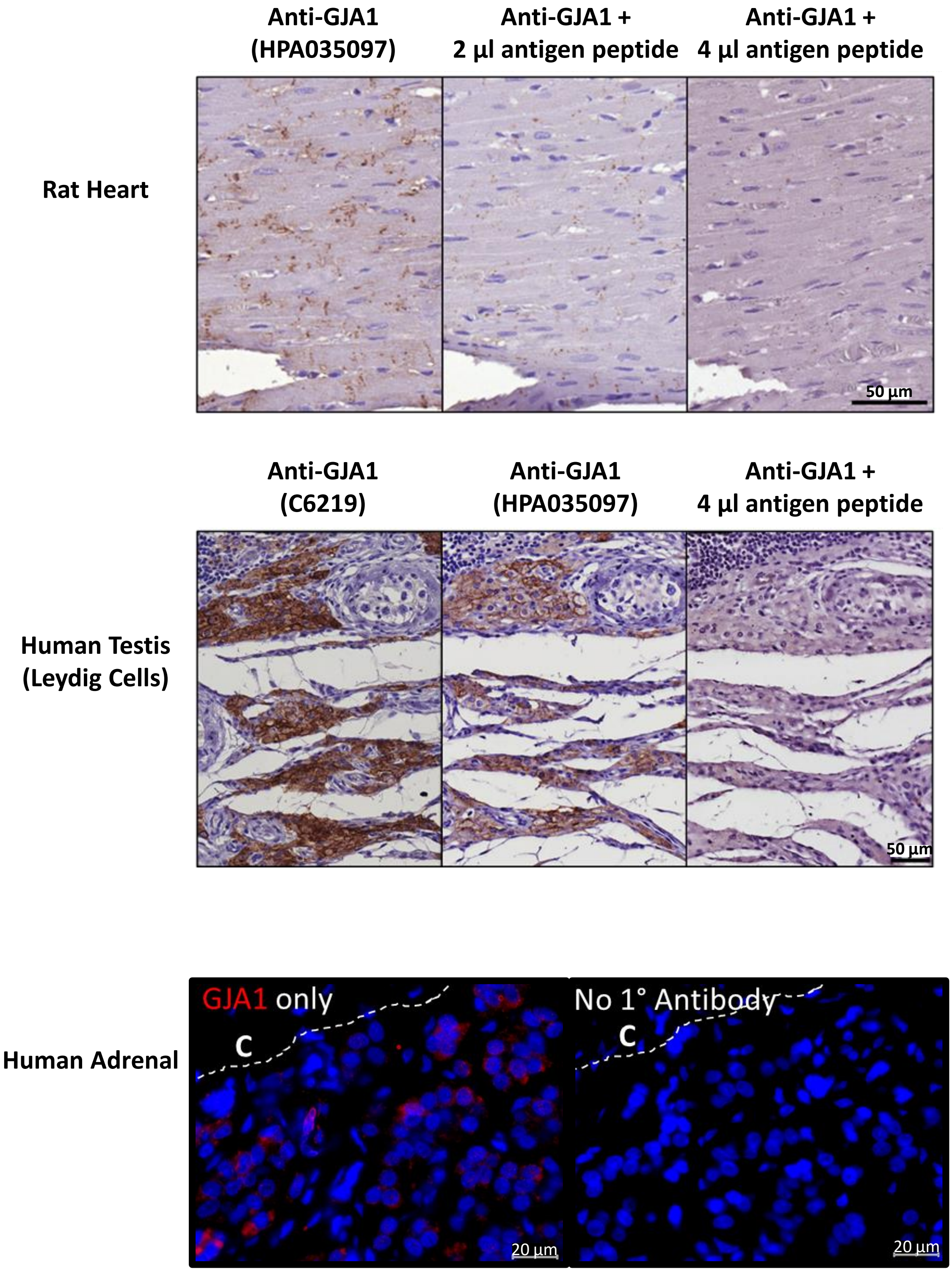

Supplementary Fig. 5

Differential expression of GJA1 in human adrenal cortex

c. **Presence of GJA1 protein expression in the ZG of the excised adrenal from the index patient P1 and a control adrenal.**

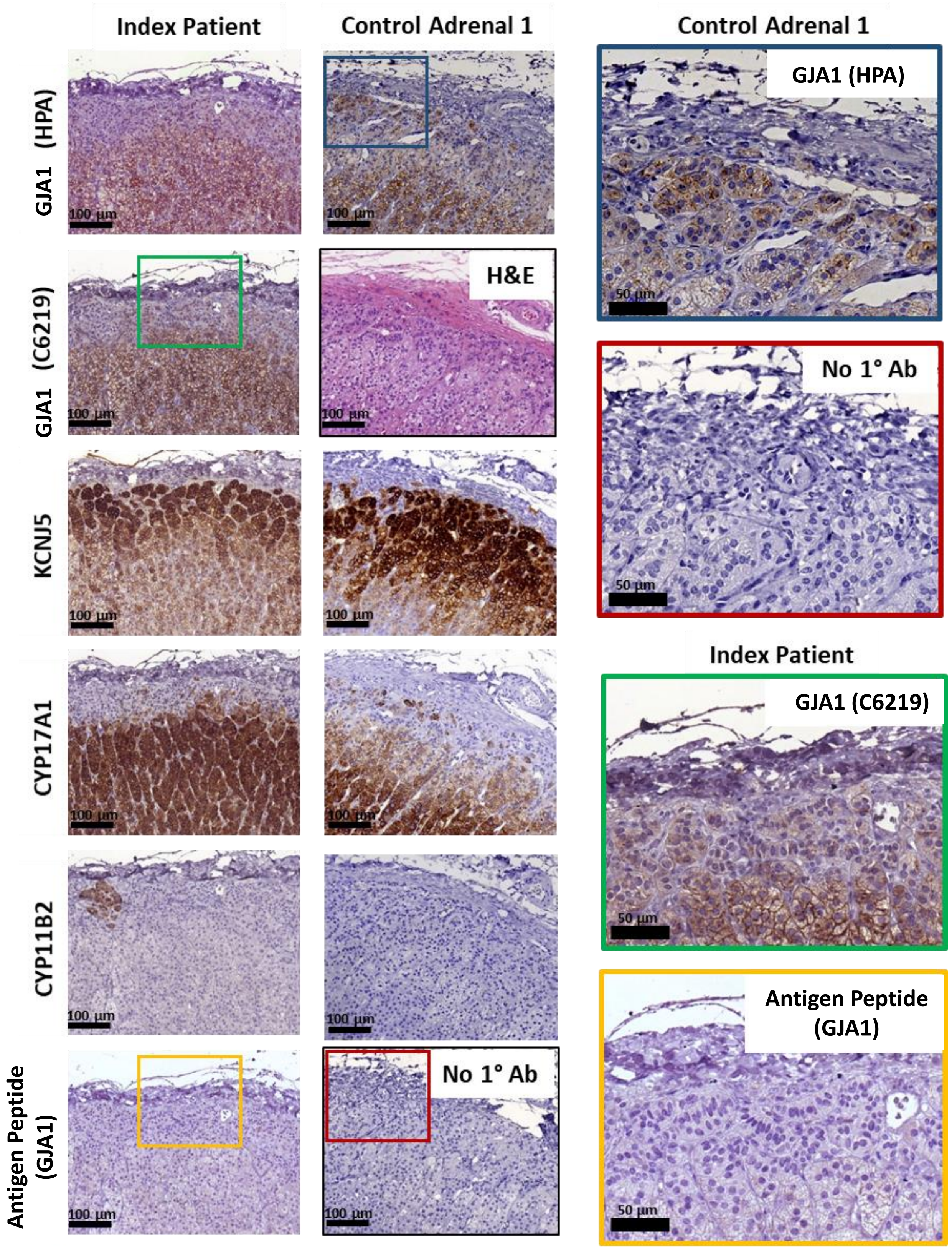

Supplementary Fig. 5
Differential expression of GJA1 in human adrenal cortex

d. IFC for CYP17A1 (ZF marker), DAB2 (ZG marker), and GJA1 in serial adrenal sections from 2 adrenals.

Adrenal of a
56 yo female
PA patient

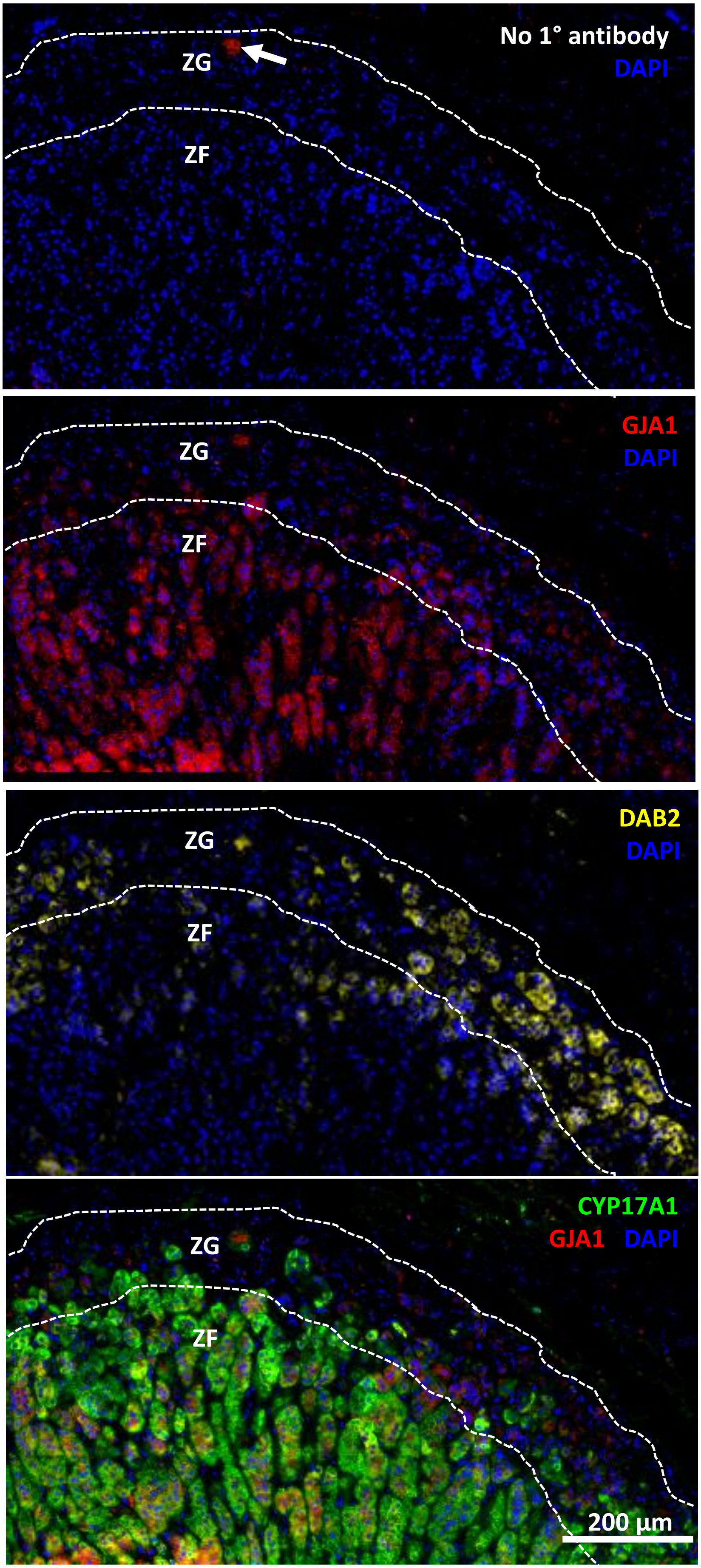

Supplementary Fig. 5
Differential expression of GJA1 in human adrenal cortex

d. IFC for CYP17A1 (ZF marker), DAB2 (ZG marker), and GJA1 in serial adrenal sections from 2 adrenals.

Adrenal of a
59 yo female
PA patient

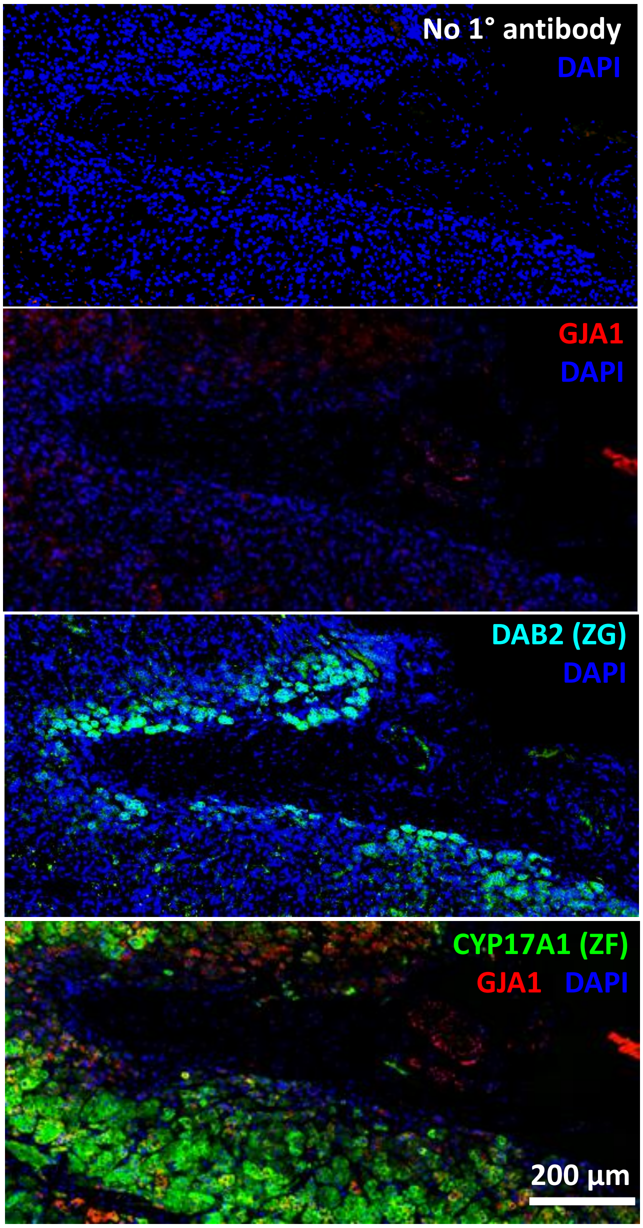

e. High magnification of IFC for CYP17A1 (ZF marker) and GJA1 identifies AGJ.

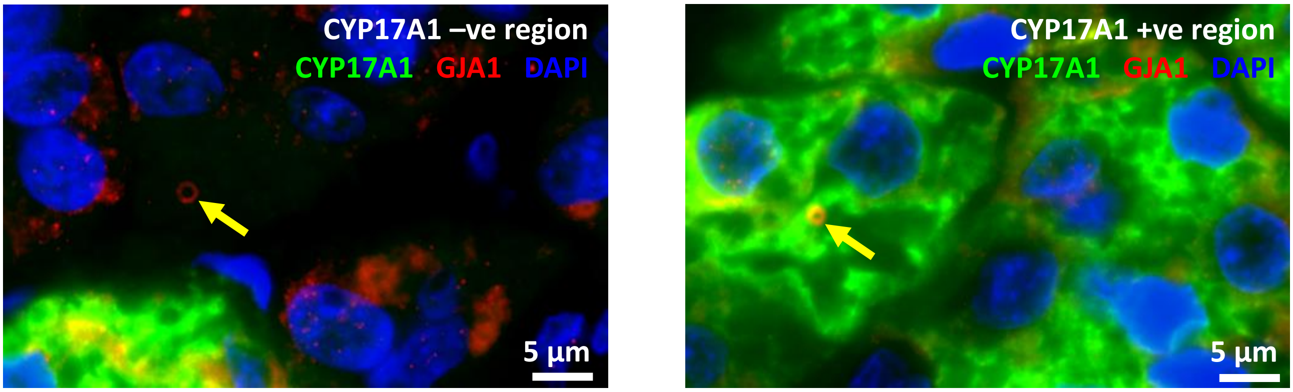

Supplementary Fig.6
GJA1 is least expressed in aldosterone- producing micronodules (APM)

a. IFC for CYP11B2 (aldosterone synthesis marker), DAB2 (ZG marker), CYP17A1 (ZF marker), and GJA1.
Serial IFC staining of an adrenal from a primary aldosteronism patient, by mouse anti-CYP11B2, rabbit anti-DAB2, mouse anti-CYP17A1 and rabbit anti-GJA1, and a no primary (1°) antibody control. The bottom panel is an enlarged image of the 3rd (CYP17A1 and GJA1) section, showing only the GJA1 IFC staining (red). The images show an aldosterone-producing micronodule (APM) with positive (+ve) staining for CYP11B2 (magenta) and DAB2 (cyan), and negative (-ve) staining for CYP17A1 (green). There was a paucity of GJA1 expression in APM compared to surrounding tissue. Highest expression of GJA1 was seen in CYP11B2 –ve, DAB2 –ve, CYP17A1 +ve regions, likely to be ZF. The bottom of the image includes the edge of the aldosterone-producing adenoma (APA) in this patient. Zoomed image of white box region is shown in Fig. 5a. Scale bar as indicated on images.

b. IFC for CYP11B2, WGA (cell membrane marker), and GJA1.
IFC for CYP11B2 (green), WGA (white), and GJA1 (red) was performed in serial sections of adrenal shown in Extended Data Fig. 7a. Zoomed image of adjacent adrenal cortex (red box) and CYP11B2 positive APM regions (yellow box) are in the panels below with or without WGA staining. Membranous expression of GJA1 seen in adjacent adrenal was not seen in CYP11B2 positive APM regions. Presence of annular GJ in a CYP11B2 positive cell is highlighted by a red arrow. Scale bar as indicated on images.

c. Example of the ZF, ZG, APM IFC staining of GJA1 colocalizing with WGA (cell membrane marker).
IFC for CYP17A1, VSNL1, or CYP11B2 (as indicated), counterstained with DAPI (blue), WGA (white), or GJA1 (Red) was performed in serial sections of the adrenal shown in Extended Data Fig. 2e. The Top panels show regions of ZF (CYP17A1 +ve), ZG (VSNL1 +ve), and APM (CYP11B2 +ve). The Middle panels show the same images as the top panels, with the green channel turned off, allowing visualization of the decreasing expression of GJA1 in the different adrenal zones (ZF>>ZG>APM). The Bottom panels show no 1° antibody controls, performed on separate serial sections from the regions shown on the Top panels. Only minute levels of autofluorescence was visualized.

d. Semi-quantification of GJA1 protein in ZG and APMs in adrenal tissues adjacent to an APA.
IHC staining scores were performed by 2 independent histopathologists (as indicated by the circle and square symbols). *Left* panel, regions were classified as APM (CYP11B2 IHC score >+2), or based on their CYP17A1 IHC staining scores. *P*-value shown for overall Chi-square test, $\chi 2 = 49.03$, *df* 9, *n*=6 adrenals. *Right* panel, regions were classified as ZG, intermediate zone (ZI) or ZF based on KCNJ5 and CYP17A1 IHC score as specified in Supplementary Table 12. *P*-value shown for overall Chi-square test, $\chi 2 = 100.2$, df 9, n=8 adrenals. Different colours codes for the 14 different adrenals.

e. Example of the IHC staining score 0, +1, +2, +3.
A serial sections of an adrenal containing an APM stained with CYP11B2, KCNJ5, CYP17A1, and GJA1 (HPA). The IHC for GJA1 was confirmed to have worked as the non-CYP11B2 positive cells (magnified in box) had GJA1 staining.

Supplementary Fig. 6. GJA1 is least expressed in aldosterone-producing micronodules (APM)

a. IFC for CYP11B2 (aldosterone synthesis marker), DAB2 (ZG marker), CYP17A1 (ZF marker), and GJA1.

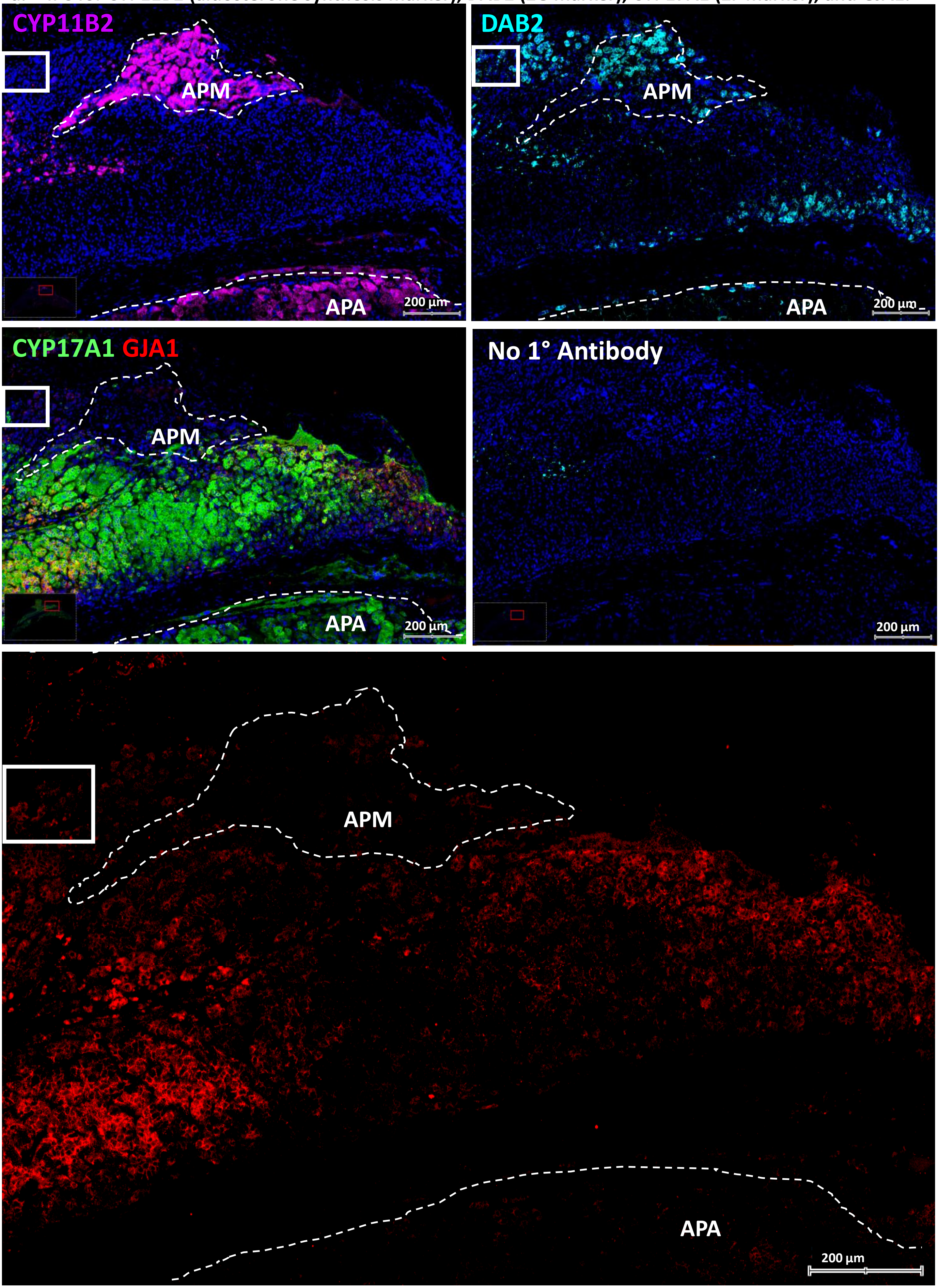

Supplementary Fig. 6. GJA1 is least expressed in aldosterone- producing micronodules (APM)

b. IFC for CYP11B2, WGA (cell membrane marker), and GJA1.

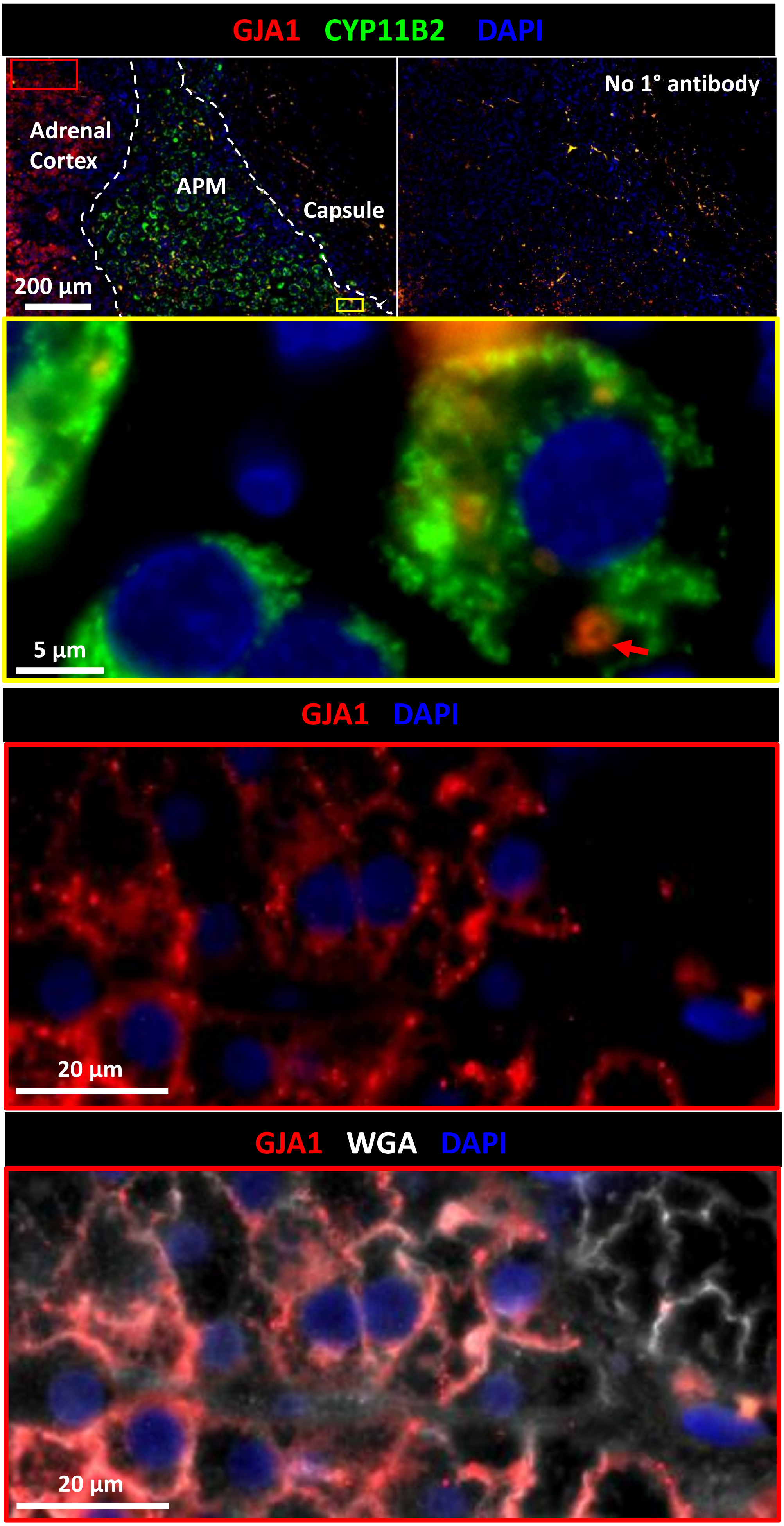

Supplementary Fig. 6
GJA1 is least expressed in aldosterone- producing micronodules (APM)

c. Example of the ZF, ZG, APM IFC staining of GJA1 colocalizing with WGA (cell membrane marker).

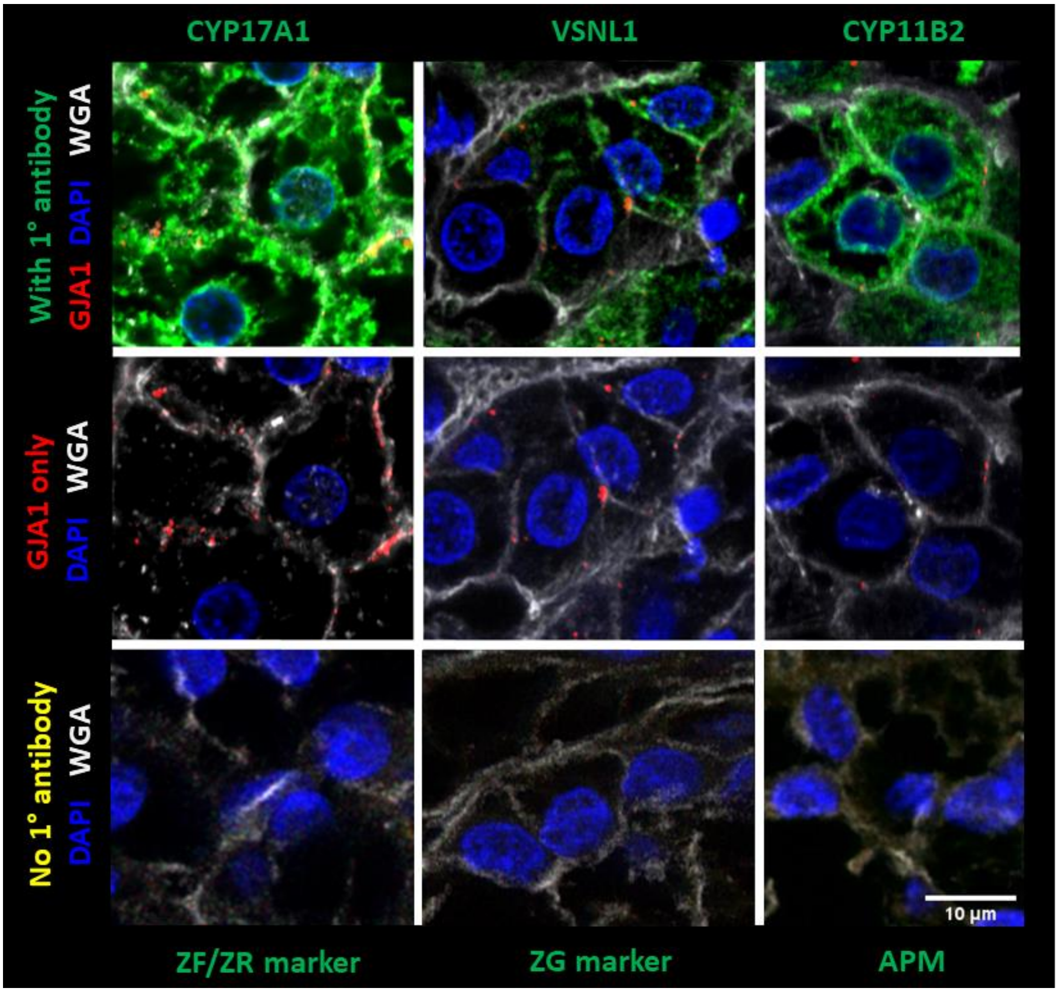

d. Semi-quantification of GJA1 protein in ZG and APMs in 14 adrenal tissues adjacent to an APA.

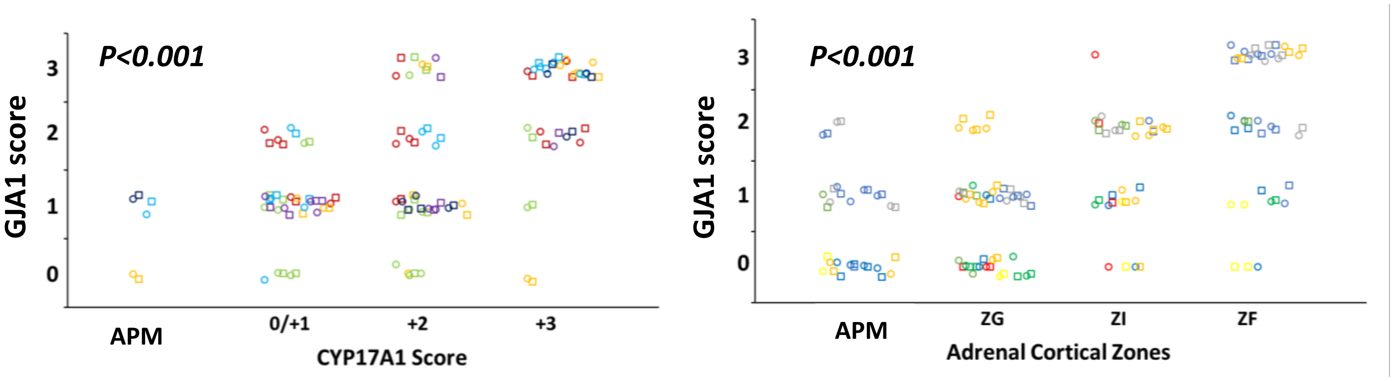

e. Example of the IHC staining score 0, +1, +2, +3.

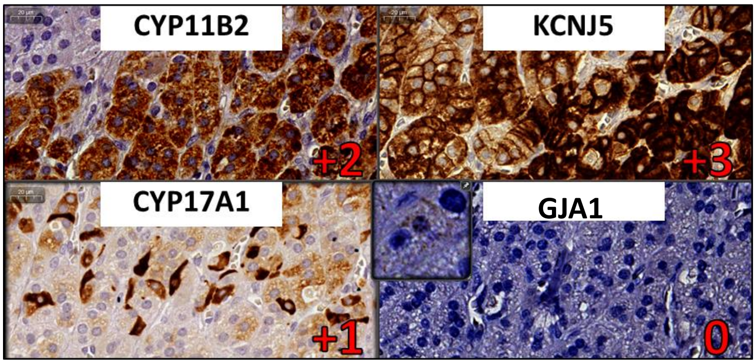

Supplementary Fig. 7
TJP1 is expressed in ZG but reduced in APM

a. GJA1 and TJP1 protein expression in index patient P1 and 3 APAs from 3 control adrenals.
Both GJA1 and TJP1 were expressed variably in CYP11B2 expressing areas of APAs from 3 control adrenals (Control Adrenal 2-4). + denotes area of interest, which is shown in higher power in the right hand panels. Scale bar: 2 mm in low power view and 20 µm in high power view.

b. TJP1-selective staining in ZG cells.
TJP1-selective staining in ZG cells of index case was seen in serial sections stained for CADM1, GJA1, KCNJ5 and CYP17A1. ZG region was characterised by intense staining of CADM1 and KCNJ5, and weak staining of GJA1 and CYP17A1. To note, the weak staining for GJA1 seen in the peri-capsular outer ZG region disappeared when GJA1 (HPA) antibody was blocked with the antigen peptide control (not shown in figure). Scale bar: 20 μm.

c. TJP1 similar to GJA1 staining is higher in ZG compared to APMs.
GJA1 staining was higher in ZG compared to APM's in index patient and control adrenal 2 from a primary aldosteronism patient harbouring a *KCNJ5* mutant APA. This was confirmed with 2 different GJA1 antibodies [GJA1 (C6219) and GJA1 (HPA)]. Tight junction protein 1 (TJP1), that has a PDZ domain that can bind to a PDZ binding motif as in CADM1, was also similarly highly expressed in ZG compared to APMs. Scale bar: 100 µm in low power view and 20 µm in high power view.

Supplementary Fig. 7
TJP1 is expressed in ZG but reduced in APM

a. GJA1 and TJP1 protein expression in index patient P1 and 3 APAs from 3 control adrenals.

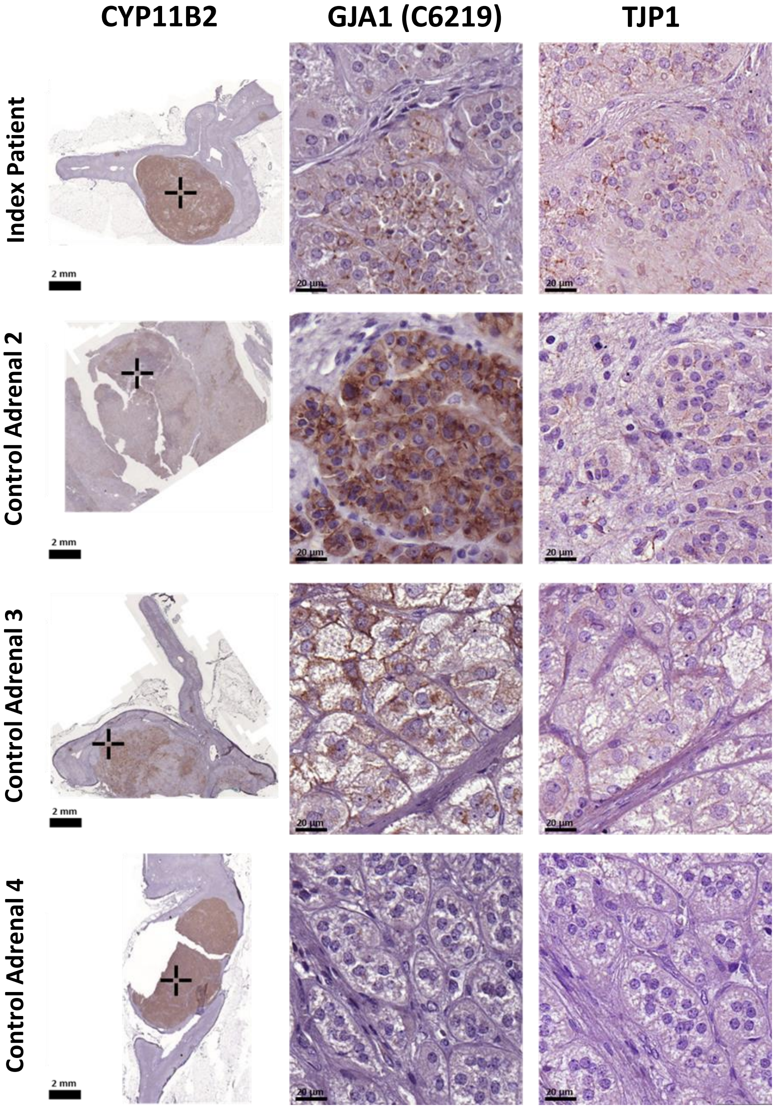

b. TJP1-selective staining in ZG cells.

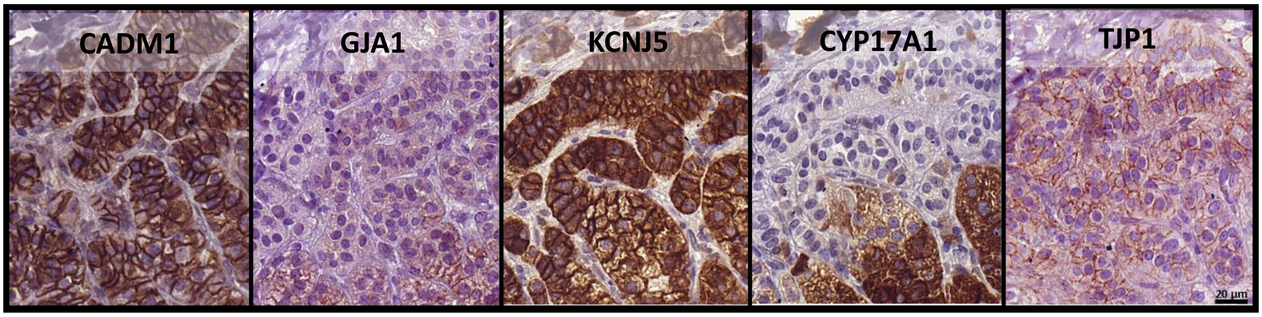

Supplementary Fig. 7
TJP1 is expressed in ZG but reduced in APM

c. TJP1 similar to GJA1 staining is higher in ZG compared to APMs.

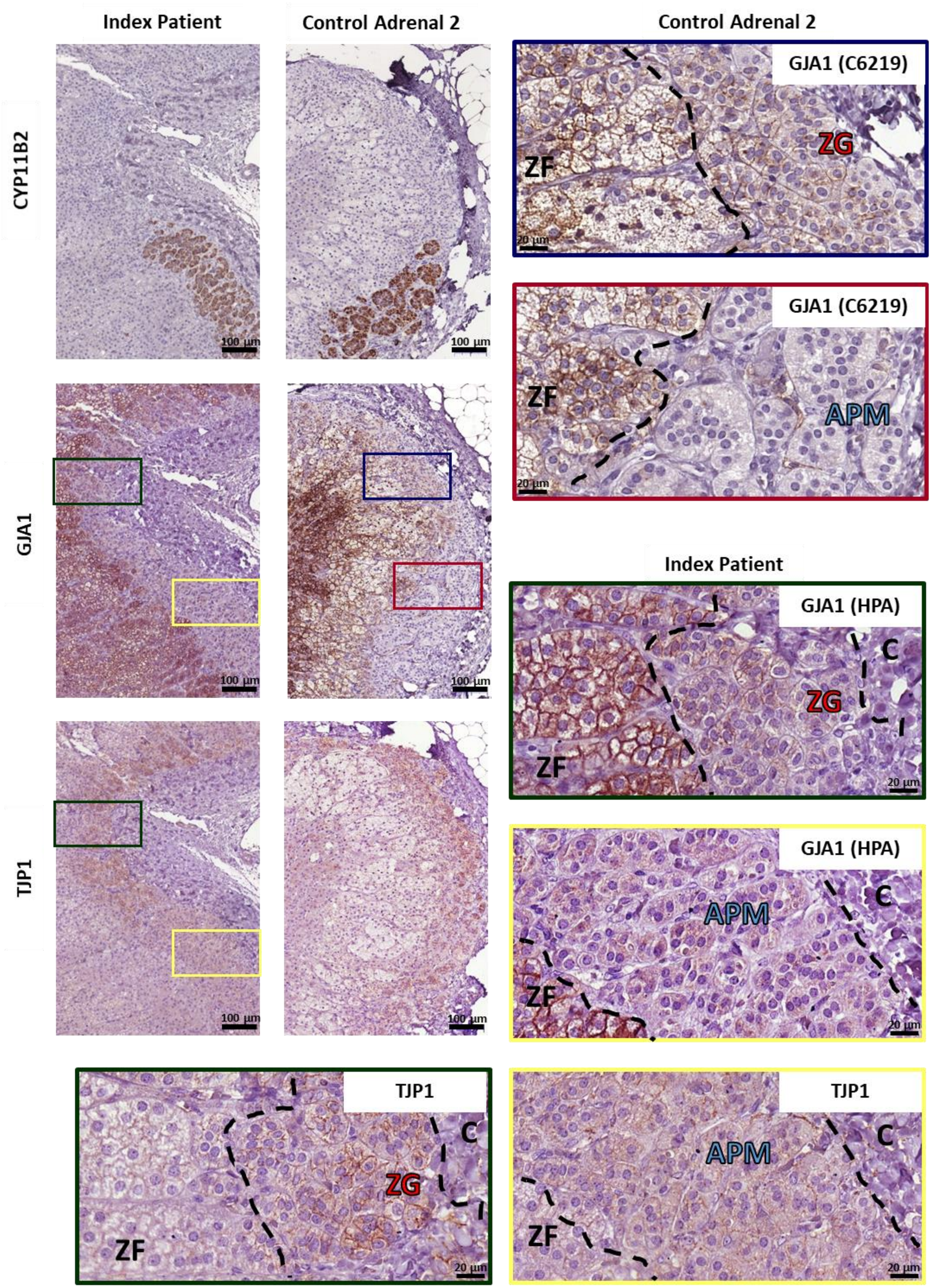

Supplementary Fig. 8. Inhibition of gap junction communication in adrenocortical cells

a. Effect of mimetic peptide Gap27 on *CYP11B2* expression and aldosterone production in the absence and presence of angiotensin II (Ang II).

In an independent pilot experiment to Fig. 5b, un-stimulated and angiotensin II (Ang II)–stimulated H295R cells were treated with Gap27 for 24 hours. In the presence of Ang II, both *CYP11B2* (*Left* panel, *n*=12) and aldosterone (*Right* panel, *n*=24) were increased, whereas in the absence of Ang II stimulation, only *CYP11B2* mRNA expression was increased with the treatment of Gap27. Statistical significance between groups were measured using the Kruskal-Wallis H test. CYP11B2; Unstimulated, $\chi2(3)=26.07$, $p=9 \times 10^{-6}$ and Stimulated, $\chi2(3)=33.44$, $p=2.6 \times 10^{-7}$. Aldosterone; Stimulated, $\chi2(3)=26.948$, $p=6 \times 10^{-6}$. Post-hoc analysis performed using two-sided Dunn's multiple comparisons test, compared to 0 µM Gap27. For CYP11B2: Unstimulated ****$p<0.0001$, Stimulated *$p=0.0054$, ****$p<0.0001$. For Aldosterone: Stimulated ***$p=0.0002$, ****$p<0.0001$.

b. Silencing of *GJA1* and *GJC1* mRNA expression in H295R cells.

Silencing of *GJA1* or *GJC1* alone, using targeted SiRNAs, successfully reduced expression of the GJs at mRNA level, by 70% and 52% respectively (*n*=3). Comparison using two-sided Student's T-test. For GJA1 *p*=0.0009, GJC1 *p*=0.0021.

c. Protein expression of *GJA1* and *GJC1* in silenced H295R cells.

Total protein from cell lysates of silenced H295R cells were immunoblotted for GJA1 and GJC1 (*n*=3). An almost complete absence of GJA1 protein expression was seen in SiGJA1 cells. There was an apparent reduction of GJC1 protein in SiGJC1 cells.

d. *CYP11B2* mRNA expression and aldosterone production in silenced H295R cells.

Single silencing of *GJA1* or *GJC1* alone did not have a significant effect on *CYP11B2* mRNA expression (*F*=3.148, *p*=0.0920, *n*=3 except for SiScr, n=6) or aldosterone secretion (*F*=0.6194, *p*=0.5478, *n*=6 except for SiScr, n=12). Statistical significance between groups were measured using one-way ANOVA.

e. *GJA1* and *GJC1* mRNA expression in co-silenced H295R cells (SiGJA1/GJC1).

Reduced *GJA1* (left panel) and *GJC1* (right panel) mRNA expression was seen in H295R cells silenced for both *GJA1* and *GJC1* using targeted SiRNAs (*n*=10). Statistical significance measured using two-sided Student's T-test. ****$p<0.0001$.

f. *GJA1* and *GJC1* protein expression in co-silenced H295R cells (SiGJA1/GJC1).

Total protein from cell lysates of co-silenced H295R cells were immunoblotted for GJA1 and GJC1 (*n*=3). An almost complete absence of GJA1 and GJC1 protein expression were seen in *SiGJA1/GJC1* co-silenced cells.

g. Effect of Gap27 on *CYP11B2* expression and aldosterone production in un-stimulated cells from normal adrenals adjacent to an APA.

Treatment with 250 µM Gap27 for 24 hours on un-stimulated primary human adrenal cells (from 3 different adrenals; Adr 1, Adr 2, and Adr 4) increased *CYP11B2* expression (*p*=0.0071) and aldosterone production (*p*=0.0241). Statistical significance measured using two-sided Student's T-test. mRNA expression for primary adrenal cells is normalised by *β-actin*. (*n*=10)

h. Effect of Gap27 on *CYP11B2* expression and aldosterone production in angiotensin II (Ang II)-stimulated cells from normal adrenals adjacent to a cortisol-producing adenoma.

Treatment of Gap27 250 µM for 24 hours did not increase *CYP11B2* expression nor aldosterone production in angiotensin II (Ang II)-stimulated primary adrenal cells adjacent to a cortisol-producing adenoma (Adr 5; *n*=3). A large *CYP11B2* Ang II response was seen in these cells which was reduced when the confluency of the cells was reduced (≤50% confluency). Statistical analysis performed using one-way ANOVA. CYP11B2, *F*=83.648, $p<0.0001$ and aldosterone, *F*=9.9998, *p*=0.0044. Post-hoc analysis performed using two-sided Sidak's multiple comparisons test $^{\#\#\#}p<0.0001$, $^{\#\#}p=0.005$, **$p=0.0099$, *$p=0.0261$

Results are expressed as fold-change relative to the untreated/SiScr cells. Data represents mean, error bars show S.E.M. n = biological replicates (independent wells).

Supplementary Fig. 8
Inhibition of gap junction communication in adrenocortical cells

a. Effect of mimetic peptide Gap27 on CYP11B2 expression and aldosterone production in un-stimulated and angiotensin II (Ang II)–stimulated H295R cells.

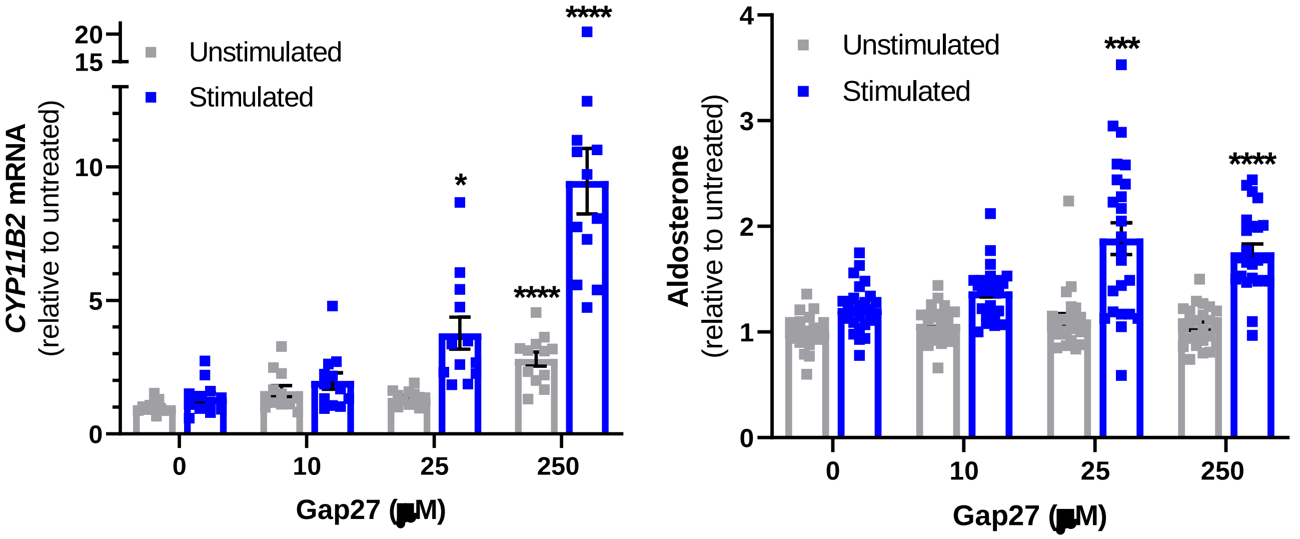

b. Silencing of *GJA1* and *GJC1* mRNA expression in H295R cells.

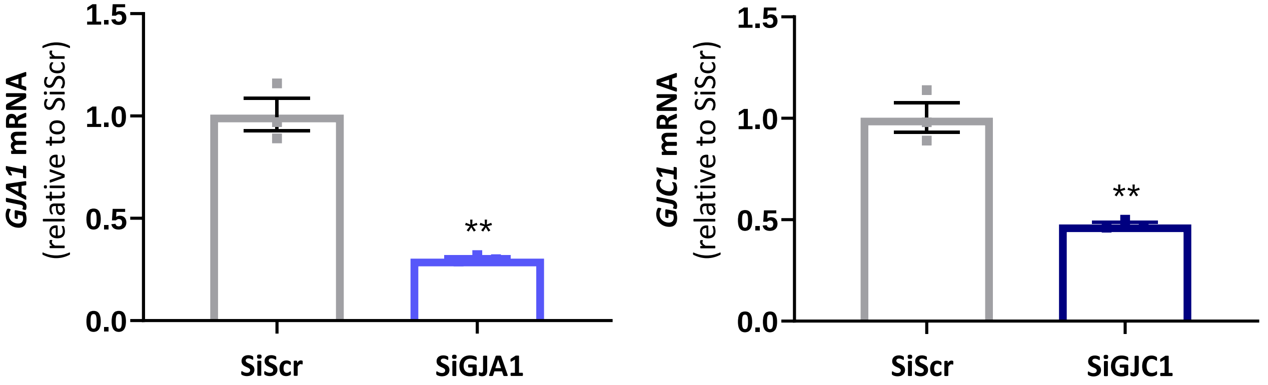

c. Protein expression of GJA1 and GJC1 in silenced H295R cells.

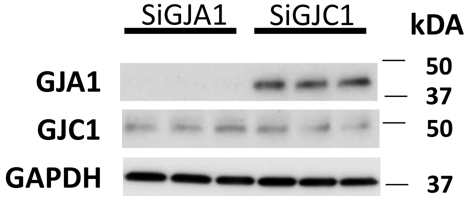

d. *CYP11B2* mRNA expression and aldosterone production in silenced H295R cells.

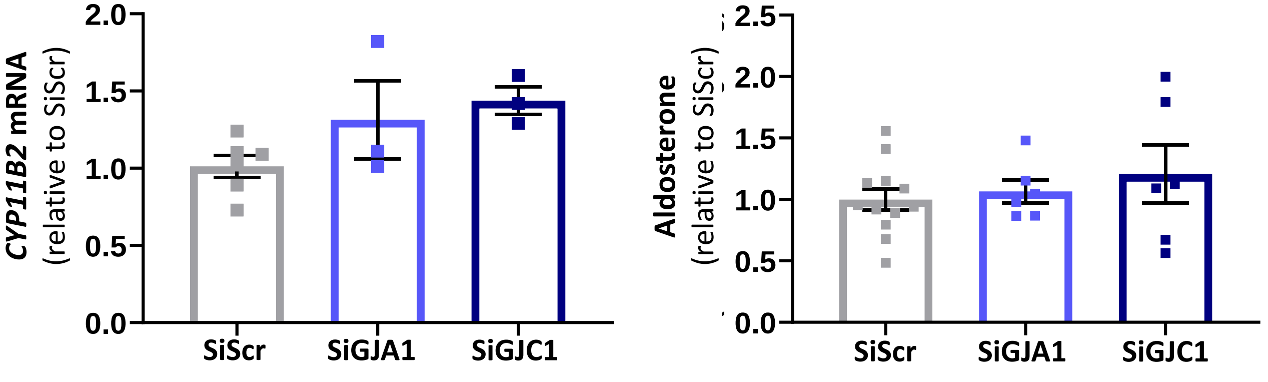

Supplementary Fig. 8
Inhibition of gap junction communication in adrenocortical cells

e. *GJA1* and *GJC1* mRNA expression in co-silenced H295R cells (SiGJA1/GJC1).

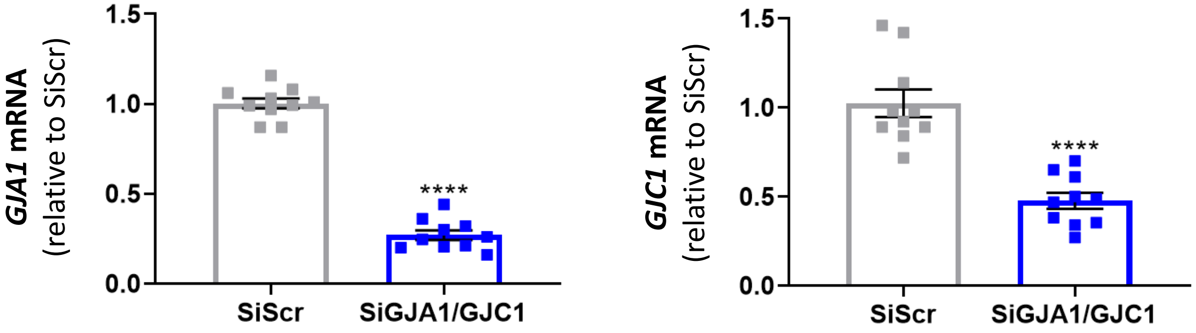

f. *GJA1* and *GJC1* protein expression in co-silenced H295R cells (SiGJA1/GJC1).

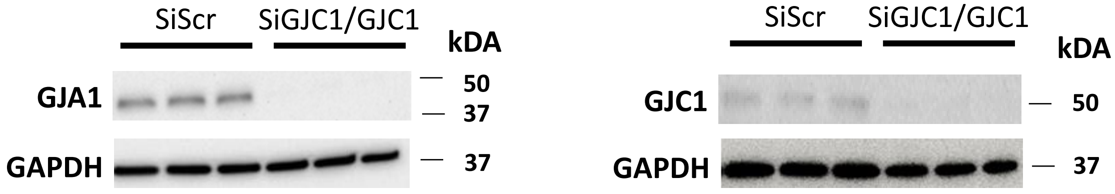

g. Effect of Gap27 on CYP11B2 expression and aldosterone production in un-stimulated cells from normal adrenals adjacent to an APA.

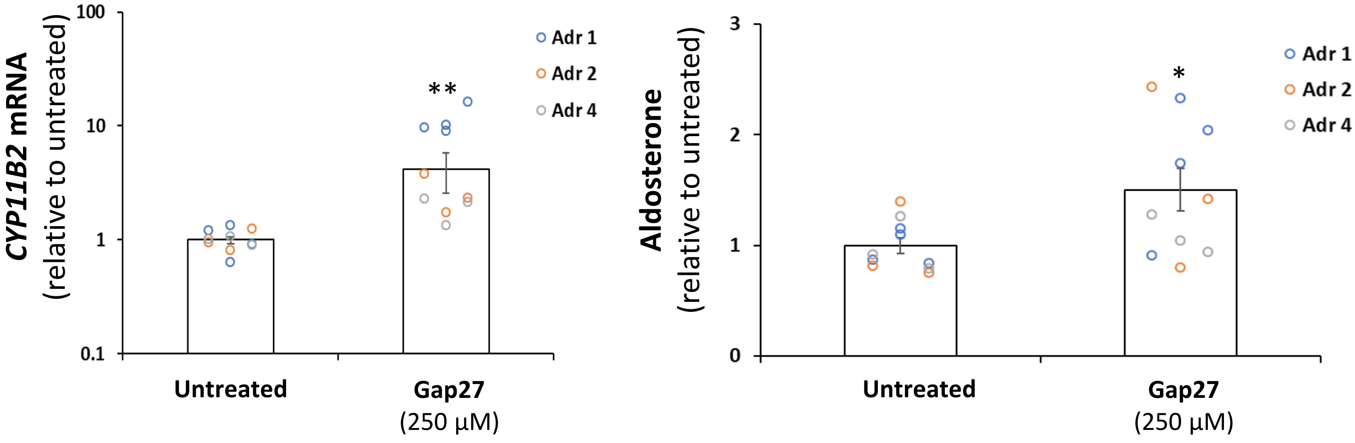

h. Effect of Gap27 on CYP11B2 expression and aldosterone production in angiotensin II (Ang II)-stimulated cells from normal adrenals adjacent to a cortisol-producing adenoma.

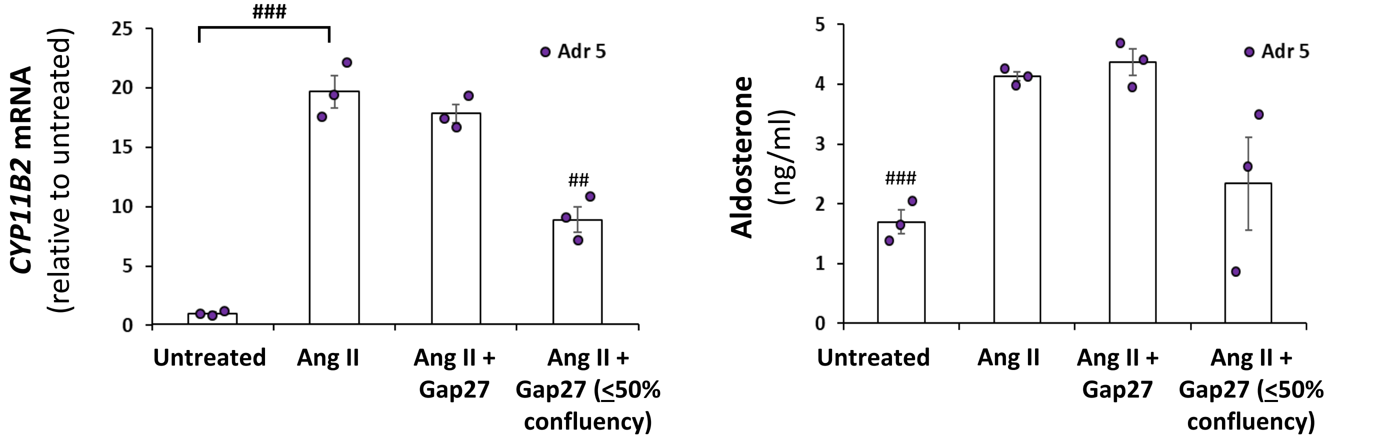

Supplementary Fig. 9
Inhibition of gap junctions in H295R cells increased baseline calcium activity but did not affect calcium oscillation in cells

a. Selection of regions of interest (ROI).
Fluo 4AM was used to measure calcium flux and oscillations in H295R cells. Regions of interest (ROIs) in a cell was selected for mean cell intensity (RFU) measurements.

b. Representative graphs of calcium measurements.
Examples of different traces analysed. *Top left* panel shows a trace with little deflection from the baseline level, suggesting no calcium oscillation activity within the cell. *Top right* panel shows a trace with calcium oscillations as there are changes in mean cell fluorescence intensity of the cell. *Bottom left* panel shows a trace with low baseline calcium intensity whereas *bottom right* panel shows a trace with high baseline calcium intensity. To note, the mean cell intensity of all oscillating cells returned to the baseline, demonstrating all cells were viable.

c. Effect of Gap27 on calcium activity in unstimulated H295R cells.
Treatment with Gap27 did not alter the number cells with calcium oscillation (Left panel). However an overall increase in the baseline calcium intensity of the cells were observed (*Right* panel). *n*=44 cells in each treatment group. Data represents mean, error bars show S.E.M. Statistical analysis was performed using tow-sided Fisher's Exact Test for comparing number of oscillating cells between different conditions (p=0.5223). Two-sided Mann-Whitney U test was used for comparing baseline calcium intensity: ****p<0.0001.

d. Effect of Gap 27 on calcium activity in angiotensin II (AngII)-stimulated H295R cells.
Left panel, in an independent experiment to Supplementary Fig. 9c, the number of cells with calcium oscillation was significantly increased when treated with AngII (****p<0.0001). No additional increased was observed in Gap27 treated AngII-stimulated cells (p= 0.11668). *Right* panel, cells treated with Ang II also demonstrated an overall increase in the baseline calcium intensity (p= 0.0125). The increase in baseline calcium intensity was also again observed in Gap27 treated cells (**p=0.0017). The addition of Gap27 to AngII stimulated cells did not significantly further increase baseline calcium intensity (p=0.1985). *n*=45 in each treatment group. Data represents mean, error bars show S.E.M. Statistical analysis was performed using two-sided Fisher's Exact Test for comparing number of oscillating cells between different conditions. Kruskal-Wallis test and two-sided Dunn's multiple comparison test were used for post-hoc analysis of baseline calcium intensity. n.s., not significant.

Supplementary Fig. 9

Inhibition of gap junctions in H295R cells increased baseline calcium activity but did not affect calcium oscillation in cells

a. Selection of regions of interest (ROI).

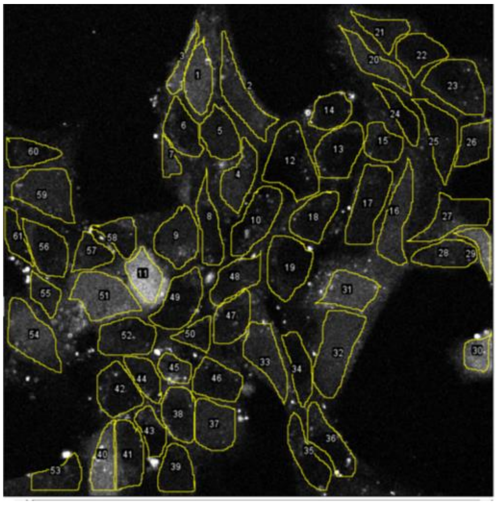

b. Representative graphs of calcium measurements.

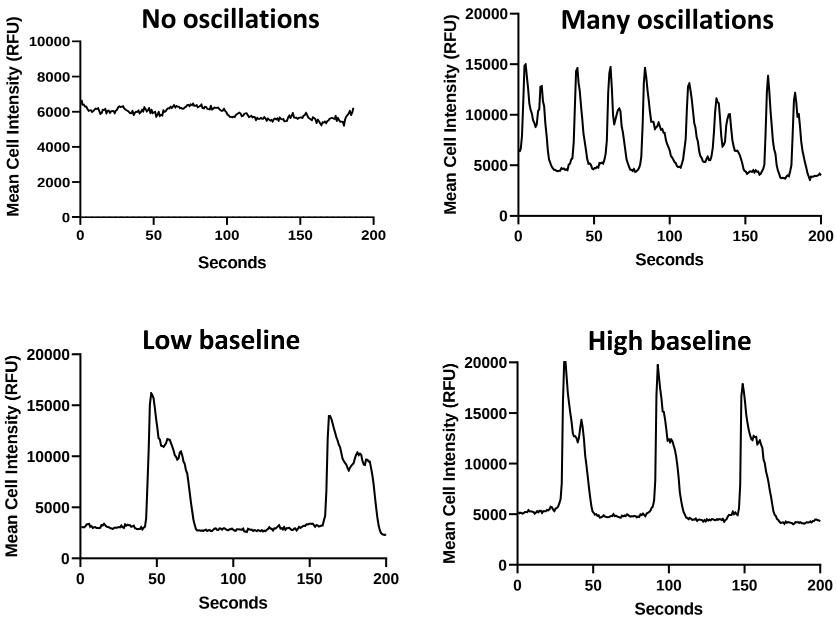

c. Effect of Gap27 on calcium activity in unstimulated H295R cells.

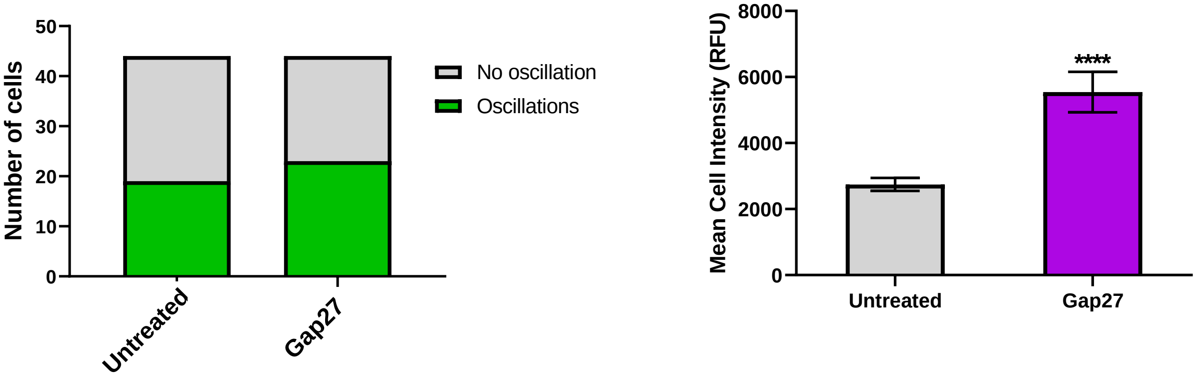

d. Effect of Gap 27 on calcium activity in angiotensin II (AngII)-stimulated H295R cells.

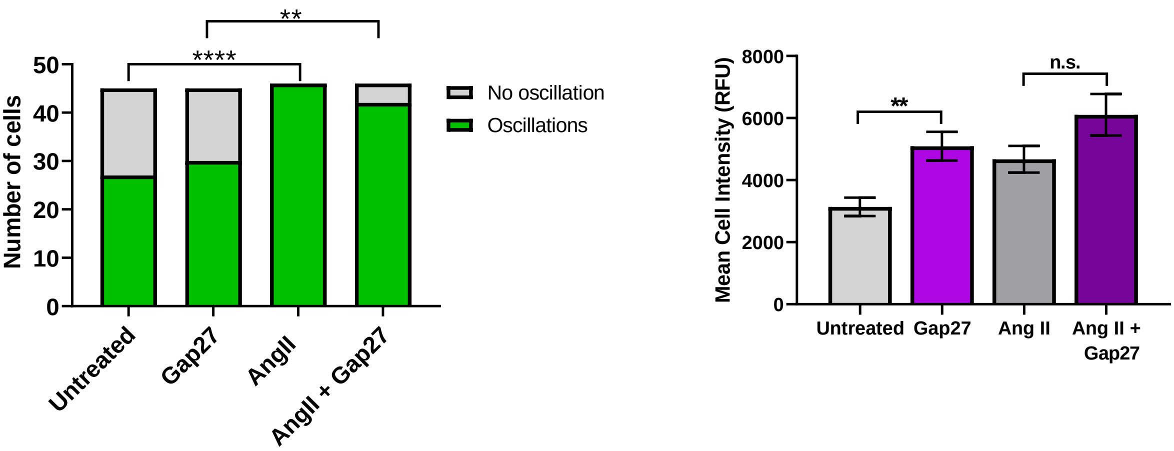

Supplementary Fig. 10

Heat map of differentially expressed genes in H295R cells transduced with sh*CADM1* or non-targeting shRNA

The columns represent different conditions in the silencing experiments. Each row represents a gene whose expression was either upregulated >1.5 fold or downregulated <0.7 fold in *CADM1*-silenced cells compared to non-targeting controls. Red represents upregulation and green represents downregulation of genes. The RNAseq results for *CYP11B2* (arrowed) replicates the decrease seen by qPCR (Fig. 2d).

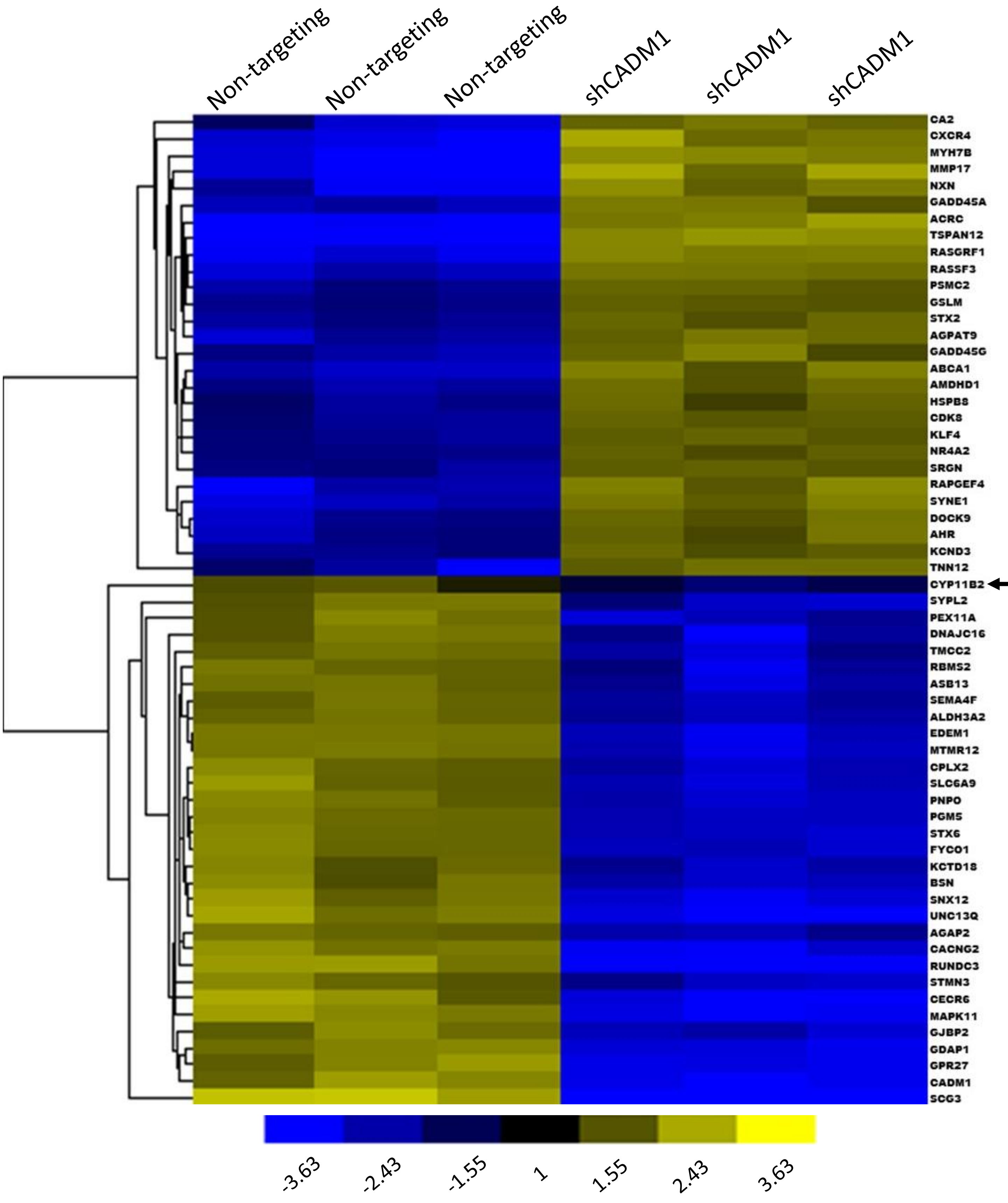

Supplementary Fig. 11

Graphs showing the diurnal expression of the clock genes in the mouse adrenal

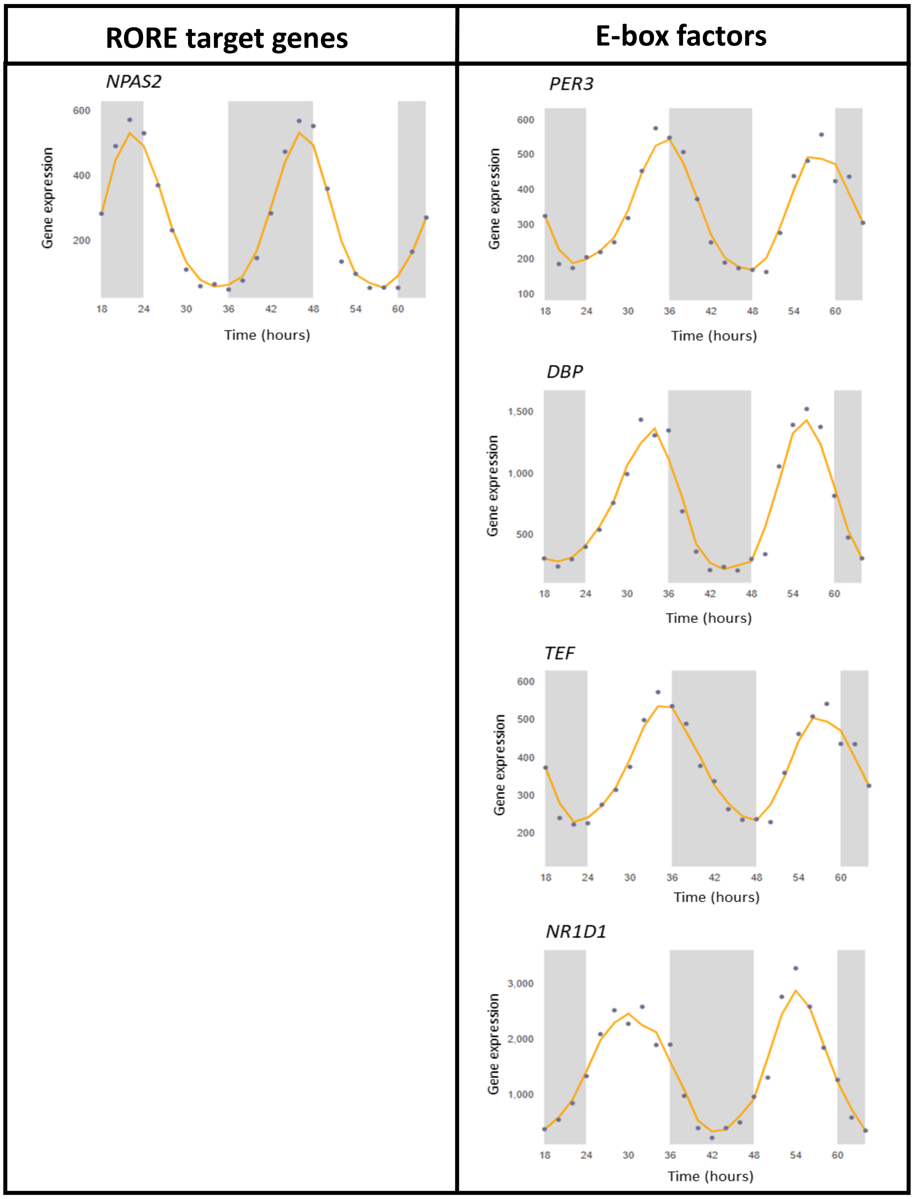

Reproduced with kind permission from http://circadb.hogeneschlab.org.

Supplementary Table 1
Deleterious CADM1 somatic mutation identified in 40 sequential APAs (Cambridge Cohort)

Whole exome sequencing of 40 sequential APAs. The genotype of recurrent aldosterone-driving mutations was previously published[17].

Sample	Age	Gender	Ancestry	Gene	Mutation
**176T**	64	Male	European	*ATP1A1*	L104R
**182T**	41	Male	European	*ATP1A1*	L104R
**187T**	33	Male	European	*ATP1A1*	L104R
**200T**	59	Male	European	*ATP1A1*	960_964del
**228T**	50	Male	European	*ATP1A1*	L104R
**141T**	53	Male	European	*ATP2B3*	del423-424
**148T**	54	Male	European	*ATP2B3*	del423-424
**156T**	66	Male	European	*ATP2B3*	del423-424
**192T**	63	Female	European	*ATP2B3*	del423-424
**181T**	47	Male	European	*CACNA1D*	G403R
**194T**	55	Male	European	*CACNA1D*	V1338M
**195T**	36	Female	African-Caribbean	*CACNA1D*	I650N
**196T**	63	Male	European	*CACNA1D*	R990G
**202T**	53	Male	European	*CACNA1D*	F1147L
**214T**	49	Male	European	*CACNA1D*	F747L
**223T**	65	Male	African-Caribbean	*CACNA1D*	I750M
**233T**	47	Male	European	*CACNA1D*	P1336R
**239T1**	55	Male	European	*CACNA1D*	G403R-8B
**240T**	57	Male	European	*CACNA1D*	F747L
**252T**	52	Male	European	*CACNA1D*	V1338M
**184T**	**48**	**Male**	European	***CADM1***	**V380D**
**14T**	20	Female	European	*CTNNB1*	T41A
**238T**	35	Female	European	*CTNNB1*	S45P
**171T**	34	Female	European	*KCNJ5*	G151R
**185T**	42	Female	European	*KCNJ5*	G151R
**190T**	47	Female	European	*KCNJ5*	G151R
**191T**	39	Female	European	*KCNJ5*	L168R
**198T**	41	Female	European	*KCNJ5*	L168R
**204T**	28	Female	European	*KCNJ5*	L168R
**219T**	49	Female	European	*KCNJ5*	L168R
**221T**	63	Male	European	*KCNJ5*	L168R
**244T**	55	Female	European	*KCNJ5*	L168R
**30T**	55	Male	European		
**136T**	59	Male	European		
**183T**	56	Male	European		
**218T**	56	Female	African-Caribbean		
**227T**	59	Male	European		
**235T**	34	Male	European		
**247T**	61	Male	European		
**250T**	69	Male	European		

Supplementary Table 2
Sociodemography of WES PA patients (Cambridge cohort) by aldosterone-driving mutations genotype

Genotype	ATP1A1	ATP2B3	CACNA1D	CTNNB1	KCNJ5	CADM1	Unknown	All tumours
No. of tumours	5 (12.5%)	4 (10%)	11 (27.5%)	2 (5%)	9 (22.5%)	1 (2.5%)	8 (20%)	40
Age (years)								
Median	50	58.5	53	27.5	42	48	56	53
Range	33-64	53-66	36-65	20-35	28-63	N/A	34-69	20-69
Gender								
Female	0 (0%)	1 (25%)	1 (9%)	2 (100%)	8 (88.9%)	0 (0%)	2 (22.2%)	13 (32.5%)
Male	5 (100%)	3 (75%)	10 (91%)	0 (0%)	1 (11.1%)	1 (100%)	7 (77.8%)	27 (67.5%)

Supplementary Table 3
All verified somatic mutations affecting coding regions discovered in 184T from patient P1.

Gene	Chr	Position (GRCh37)	Reference Allele	Alternative Allele	Protein Change	Reads (Total/Variant)
CADM1	11	115049435	A	T	p.V380D	123/28
GRIK2	6	102372571	G	C	p.G615A	81/11
LAMC3	9	133884793	C	A	p.H64Q	27/5

Supplementary Table 4
Estimated prevalence of CADM1 mutation across study cohorts

Cohort	Total number of APAs that underwent WES	Number of APAs that underwent TGS of *CADM1*	Number of APAs with known aldo-driver mutation excluded	Total no. of APAs reviewed for prevalence estimation	No. of codon 379 or 380 m*CADM1* identified in cohort	Estimated Prevalence of m*CADM1* APA by cohort
Cambridge	53	*12*	*31*	*96*	*1*	*1.04%*
German	81	0	15	96	1	1.04%
French	34	43	247	324	3	0.93%
USA	0	204	N/A	204	1	0.49%
Others (London/Japan)	0	41	N/A	41	0	0.00%

*TGS, targeted sequencing. aldo-, aldosterone. m*CADM1*, mutant *CADM1*. N/A, not available.

Supplementary Table 5
RNA expression of GJs in human adrenocortical cells

a. Microarray data showing average Log_2 RNA expression of GJs in the ZG and ZF in the adjacent adrenal gland (AAG) next to 7 *KCNJ5*-mutant APAs, 7 ZG-like APAs and 7 phaeochromocytoma (Phaeo).

	ZF in AAG next to...			ZG in AAG next to...		
	KCNJ5-mutant APA	ZG-like APA	Phaeo	KCNJ5-mutant APA	ZG-like APA	Phaeo
GJA1	10.06	10.16	10.38	8.54	8.20	8.40
GJA10	2.37	2.40	2.43	2.25	2.34	2.29
GJA3	3.99	3.92	4.16	4.08	3.91	3.88
GJA4	5.02	5.22	5.11	5.22	5.67	5.37
GJA5	2.33	2.32	2.40	2.46	2.75	2.66
GJA8	2.49	2.44	2.52	2.48	2.49	2.44
GJA9	2.61	2.72	2.80	2.54	2.58	2.47
GJB1	3.25	3.25	3.25	3.23	3.24	3.19
GJB2	2.76	2.77	2.74	2.69	2.79	2.66
GJB3	2.51	2.39	2.55	2.38	2.42	2.36
GJB4	3.12	2.94	3.15	3.03	2.98	2.93
GJB5	2.60	2.62	2.68	2.46	2.63	2.55
GJB6	2.93	2.92	3.08	2.87	2.99	2.78
GJB7	2.81	2.80	2.89	2.85	2.79	2.78
GJC1	3.19	3.16	3.30	3.62	3.80	3.47
GJC2	2.44	2.37	2.42	2.46	2.39	2.41
GJC3	2.93	2.82	2.91	2.79	2.74	2.70
GJD2	4.42	4.63	4.53	4.38	4.40	4.48
GJD3	2.49	2.50	2.46	2.49	2.65	2.57
GJD4	2.71	2.69	2.71	2.71	2.79	2.64
GJD4	3.01	3.03	3.07	3.11	3.19	3.01

b. RNA sequencing data showing average read numbers of all gap junctions in H295R cells (transduced with empty vector (EV) or untransduced (UT) cells). *GJC1* is the most abundant gap junction expressed in H295R cells, followed by *GJA1*.

.

	UT	EV
GJA1	2.55	2.68
GJA10	0.00	0.00
GJA3	0.10	0.15
GJA4	0.00	0.00
GJA5	0.03	0.02
GJA8	0.00	0.00
GJA9	0.00	0.00
GJB1	0.01	0.01
GJB2	0.37	0.27
GJB3	0.02	0.00
GJB6	0.02	0.00
GJB7	0.00	0.00
GJC1	7.65	6.90
GJC2	0.83	0.70
GJC3	0.07	0.04
GJD2	0.00	0.01
GJD3	0.02	0.06
GJD4	0.02	0.01

Supplementary Table 6
List of differentially expressed genes in H295R transduced cells

a. The 20 most up-regulated protein coding genes in mutant *CADM1* transduced cells, compared to wild-type (*p*<5.00E-08)*.

Gene	Fold-change	*p*- value
CYP11B2	13.78	8.10E-08
SCG2	10.08	9.19E-10
MYOM1	7.10	8.84E-12
MRAP	5.92	5.06E-10
RAPGEF4	5.53	9.39E-08
RFX6	5.35	4.20E-08
MC2R	4.96	2.25E-15
RGS4	4.61	2.20E-08
CYP11B1	4.53	4.05E-09
PRKCH	4.27	4.40E-20
IGFN1	4.22	2.00E-08
NGEF	3.57	3.42E-07
KIRREL3	3.31	2.67E-10
FOS	3.17	1.14E-07
NR4A2	2.95	1.42E-08
PITPNC1	2.91	1.56E-08
PLEKHG1	2.91	3.54E-07
COL25A1	2.90	7.37E-14
RBPJ	2.81	1.15E-16
DPP4	*2.73*	5.89E-16

Statistical analyses performed using two-sided one-way ANOVA, compared to **a.** wild-type or **b.** non-targeting cells. *p* values are listed above.

b. The 40 most down-regulated protein coding genes (*p*<0.0005) and *CYP11B2* in sh*CADM1* transduced cells, compared to non-targeting controls. A filter of an average read number >0.5 in controls and >0.1 in sh*CADM1* transduced cells was used*.

Gene	Average Non-Targeting	Average shCADM1	Fold-change	*p*-value
SCG3	2.06	0.13	0.06	0.0000
GNG4	0.82	0.15	0.19	0.0001
RUNDC3A	2.62	0.60	0.23	0.0001
MAPK11	1.02	0.25	0.24	0.0001
UNC13A	6.14	1.61	0.26	0.0002
TLR3	0.71	0.19	0.27	0.0002
CADM1	9.70	2.68	0.28	0.0002
RBM20	0.65	0.18	0.28	0.0000
CACNG2	2.92	0.85	0.29	0.0001
GPR27	4.07	1.19	0.29	0.0003
GDAP1	6.72	2.02	0.30	0.0000
PHF21B	0.76	0.23	0.30	0.0000
SNX12	13.19	4.02	0.30	0.0004
PQLC3	0.56	0.17	0.31	0.0002
PIGZ	0.52	0.17	0.32	0.0005
SNHG16	12.93	4.19	0.32	0.0003
KIAA0040	1.12	0.37	0.33	0.0002
MTMR12	8.82	2.94	0.33	0.0000
EDEM1	9.03	3.02	0.33	0.0000
KCNC2	0.63	0.21	0.34	0.0004
STX6	5.72	1.98	0.35	0.0001
FYCO1	2.48	0.86	0.35	0.0001
PNPO	12.68	4.45	0.35	0.0001
G3BP2	15.91	5.63	0.35	0.0002
PGM5	4.61	1.63	0.35	0.0000
ZDHHC23	2.33	0.83	0.36	0.0000
CPLX2	1.48	0.54	0.37	0.0003
PEX11A	1.02	0.37	0.37	0.0004
STMN3	14.82	5.55	0.37	0.0005
ASB13	8.04	3.03	0.38	0.0001
SYPL2	2.75	1.04	0.38	0.0005
SYT14	0.58	0.22	0.38	0.0003
KCTD18	1.30	0.50	0.39	0.0005
RBMS2	4.08	1.58	0.39	0.0003
TMCC2	1.33	0.52	0.39	0.0001
EIF4E3	0.96	0.37	0.39	0.0001
AGAP2	1.04	0.41	0.39	0.0000
SEMA4F	3.35	1.32	0.39	0.0000
ALDH3A2	29.77	11.76	0.39	0.0000
ADD3	7.16	2.83	0.39	0.0001
CYP11B2	4.29	2.58	0.60	0.0045

Supplementary Table 7
Most significant biological processes enriched in *CADM1* mutant-transduced cells

RNA sequencing data from H295R cells transduced with empty vector, wild-type (WT) or mutant *CADM1*. Genes with >1.5 fold increase or <0.7 fold reduction in expression in mutant *CADM1,* compared to WT, were selected for gene enrichment analysis using Database for Annotation, visualization, and Integrated Discovery (DAVID; david.ncifcrf.gov). A Modified Fisher Exact *p*-value is shown, denoting strength of enrichment of genes (observed versus expected) in the annotation categories.

Category	Term	Count	%	*p* -value	Genes	List Total	Pop Hits	Pop Total	Fold Enrichment	Bonferroni	Benjamini	FDR
UP_KEYWORDS	Biological rhythms	14	0.02	1.02E-06	CDK5R1, KLF9, KLF10, CREM, CIART, PROX1, AHR, NPAS2, NR1D1, DBP, TEF, PER3, NFIL3, SIK1	402	125	20581	5.73	3.23E-04	3.23E-04	0.0014
UP_KEYWORDS	Cell junction	34	0.05	1.34E-06	STON2, LZTS1, GRIK2, GRIK4, CABP1, GJA1, GABBR2, AFAP1L1, GRIN3A, CXADR, RIMS4, NOV, CXCR4, PPL, SV2C, CHRNA3, DPP4, DTNA, SYNPO, FLRT2, KCND2, MAGI1, KCNB1, SLC6A17, HOMER1, HOMER2, SYNGR3, CAMK2N1, TMEM47, TMEM163, CLDN1, SDCBP, ERC2, KCTD12	402	675	20581	2.58	4.25E-04	2.12E-04	0.0018
UP_KEYWORDS	Synapse	23	0.04	2.26E-06	STON2, FLRT2, LZTS1, KCND2, GRIK2, KCNB1, CABP1, GRIK4, GABBR2, SLC6A17, GRIN3A, HOMER1, HOMER2, SYNGR3, RIMS4, CAMK2N1, TMEM163, ERC2, SV2C, CHRNA3, KCTD12, DTNA, SYNPO	402	357	20581	3.30	7.13E-04	2.38E-04	0.0030
UP_KEYWORDS	Glycoprotein	127	0.20	8.36E-06	ATP1B1, GRIK2, GRIK4, EFNA3, C3ORF80, GRIN3A, VIPR1, TGFB2, ST3GAL1, NOV, S1PR3, BDNF, DIRC2, WNT4, BRINP2, UNC5B, HIST1H2BL, HIST1H2BI, SEMA7A, ST3GAL6, KCNK5, TFPI2, CHRNA3, EFNB1, OLFML2B, STIM2, OLFML2A, TMEM200B, COLEC12, TMEM132E, MC2R, STC1, EFNA5, FRAS1, SLC2A13, FGFR1, FGFR4, IL22RA1, MME, EPHB2, IL12RB2, IL17D, PROCR, BCHE, B3GNT7, CNR1, P2RY2, ADRA2A, GCNT1, ANGPTL2, LFNG, MUC1, BMP4, DAND5, VSTM4, TGFBR2, SLC6A17, CACNA2D3, PCDH18, ATP7A, LYVE1, CD55, SEMA6D, WSCD1, FAM198A, SFRP1, CACNA1G, CACNA1C, CHRD, BMP6, ACVRL1, GABBR2, APCDD1, CXADR, GPC4, POMGNT2, SLC1A3, CXCR4, CLUL1, HIST3H2BB, SV2C, MYC, DPP4, KIRREL3, EVA1C, PGAP1, CHST2, SIGLEC11, COL25A1, SEL1L3, MAN1A1, LMBRD1, SLIT2, TAS2R19, CD82, GRM7, CA4, KCNH2, KCNH3, TSHR, RHBG, SPOCK1, ISM1, GPR3, LINGO1, ADAMTS12, KCNE4, SCG2, SYNPO, CNNM2, HIST1H2BB, FLRT2, TNFSF4, HIST1H2BF, TMPRSS9, NPR1, NPR3, NID2, TMEM2, CDH12, SULF2, CDON, LRRN1, TMTC1, FAM155A, PRSS23, CDH11	402	4551	20581	1.43	2.64E-03	6.61E-04	0.0113
GOTERM_MF_ DIRECT	GO:0043565~sequence-specific DNA binding	28	0.04	1.56E-05	FOSL2, CREM, CXXC5, FOXO6, VDR, FOS, FOXQ1, OSR2, BCL2, HEY2, TEF, ETV1, NFIL3, MYC, ETV4, NR1H4, KLF5, MAFF, SOX13, EMX2, NR4A2, NR4A1, NR4A3, HES1, MSX1, HIVEP2, RBPJ, RERE	357	518	16881	2.56	9.90E-03	9.90E-03	0.0232
GOTERM_BP_ DIRECT	GO:0045944~positive regulation of transcription from RNA polymerase II promoter	43	0.07	1.60E-05	AKNA, FOSL2, ACVRL1, LMO2, CREM, ABLIM3, LMO4, MEIS1, FOS, VDR, NPAS2, OSR2, HEY2, TEF, AUTS2, ETV1, MAML3, MYB, MYC, ETV5, ETV4, NR1H4, CYR61, FOXD3, BMP4, KLF5, MAFF, SSBP3, MYO6, RFX6, NR4A2, NR4A1, NR4A3, PROX1, AHR, HES1, MSX1, DBP, CDON, RBPJ, TBL1X, RERE, BMP6	362	981	16792	2.03	3.54E-02	3.54E-02	0.0279
GOTERM_CC_ DIRECT	GO:0030054~cell junction	26	0.04	1.63E-05	STON2, LZTS1, GRIK2, CABP1, GRIK4, GABBR2, AFAP1L1, GRIN3A, CXADR, RIMS4, CXCR4, SV2C, CHRNA3, DTNA, KCND2, MAGI1, KCNB1, SLC6A17, HOMER1, HOMER2, SYNGR3, CAMK2N1, TMEM47, TMEM163, ERC2, KCTD12	386	459	18224	2.67	4.43E-03	4.43E-03	0.0215
GOTERM_BP_ DIRECT	GO:0043401~steroid hormone mediated signaling pathway	9	0.01	2.78E-05	BMP4, VDR, NR1D1, NR4A2, NR4A1, NR4A3, PAQR9, NR1H4, PAQR5	362	57	16792	7.32	6.07E-02	3.08E-02	0.0485
UP_KEYWORDS	Membrane	186	0.29	3.47E-05	CYP3A5, ATP1B1, GRIK2, GRIK4, EFNA3, C3ORF80, SLC7A8, GRIN3A, VIPR1, ST3GAL1, S1PR3, DIRC2, BDNF, UNC5B, SEMA7A, KCNK5, ST3GAL6, RAPGEF4, CHRNA3, SLC4A5, MYO6, KCND2, MAGI1, EFNB1, STIM2, TMEM200B, COLEC12, TMEM132E, TMEM133, PITPNM1, PITPNM2, MC2R, SDCBP, EFNA5, FRAS1, FGFR1, SLC2A13, CDK5R1, FGFR4, MOB4, IL22RA1, MRAP, GNAI1, MRAS, MME, PAQR9, EPHB2, PAQR5, KIAA1549L, IL12RB2, PROCR, NDRG4, BCHE, P2RY2, CNR1, B3GNT7, PPL, ADRA2A, SLC35F3, GCNT1, LFNG, CYP19A1, MUC1, REEP6, TRPC1, VSTM4, OSBPL6, KCNB1, TGFBR2, REEP1, SLC6A17, HOMER1, CACNA2D3, HOMER2, SYNGR3, CAMK2N1, PCDH18, ATP7A, CORO1C, SYNE1, MYO10, CD55, LYVE1, WSCD1, SEMA6D, CACNA1G, SYTL2, RHBDL3, CACNA1C, KCTD12, LZTS1, ACVRL1, RGS7BP, CYP11B1, CYP11B2, GJA1, GABBR2, CXADR, APCDD1, GPC4, SDIM1, EFHD2, FAR2, TSPAN12, AP1S1, SLC1A3, MCTP2, POMGNT2, CYP39A1, CXCR4, CCDC180, MYB, SV2C, DPP4, KIRREL3, ZDHHC2, EVA1C, PGAP1, CHST2, SIGLEC11, SEL1L3, COL25A1, MAN1A1, LMBRD1, TMEM246, TAS2R19, TLCD2, GRM7, CD82, CLDN1, CA4, INPP4B, KCNH2, TSHR, KCNH3, RASD2, SNX10, SLC27A4, STON2, MFSD6, FATE1, RAB3C, GDAP1, CABP1, RHBG, CABP7, EEA1, GNG11, SLC47A1, ALDH3A2, GPR3, LINGO1, GLIPR2, CKMT2, DGKG, BCL2, CAMK2D, RPIA, KCNE4, SYNPO, DTNA, CNNM2, FLRT2, NGEF, TNFSF4, TMPRSS9, NPR1, DGKH, NPR3, CYSTM1, TMEM2, CDH12, TMEM47, SAMD5, KCNN1, PDE2A, TMEM163, PTP4A1, CDON, LRRN1, MBOAT1, TMTC1, FAM155A, SNX33, PHLDB2, CDH11	402	7494	20581	1.27	1.09E-02	2.19E-03	0.0467
GOTERM_CC_ DIRECT	GO:0045211~post-synaptic membrane	16	0.03	4.46E-05	LZTS1, KCND2, GRIK2, KCNB1, CABP1, GRIK4, GABBR2, GRIN3A, HOMER1, HOMER2, CAMK2N1, SYNE1, GRM7, CHRNA3, KCTD12, SYNPO	386	211	18224	3.58	1.21E-02	6.05E-03	0.0587

Supplementary Table 8
List of genes that were upregulated in both Adr 184T from index patient P1 and *CADM1* mutant transduced H295R cells

RNA sequencing data showing average read numbers for genes which showed increase in expression in the index *CADM1*-mutant APA (184T) compared to 2 comparative, ZG-like APA controls (192T and 195T); and which showed increased expression in mutant *CADM1*-transduced H295R cells, compared to wild-type (WT). Comparisons made using two-sided one-way ANOVA. 192T, an del423_424 *ATP2B3*-mutant APA; 195T, a I650N *CACNA1D*-mutant APA.

	RNA Sequencing of human APA (T) and adjacent 'normal' adrenal (N)							RNA Sequencing of H295R cells transduced with WT or Mutant *CADM1*	
	mRNA Expression						Fold change in 184T mRNA expression compared to average of 192T and 195T		
Gene ID	184N	184T	192N	192T	195N	195T		Fold-increase in Mutants	P value
MYOM1	0.64	6.06	0.48	3.81	0.52	1.75	2.18	7.10	8.84E-12
AQP2	0.93	24.29	0.05	2.16	0.17	0.38	19.13	7.01	4.31E-04
VDR	6.26	21.79	2.61	11.44	2.66	7.02	2.36	1.97	1.37E-10
C9orf47	2.32	2.74	1.14	1.19	1.43	1.42	2.10	1.81	1.41E-12
C12orf68	1.45	3.57	1.04	1.43	1.05	1.59	2.37	1.76	6.61E-12
MPP3	0.45	1.61	0.36	0.73	0.52	0.49	2.64	1.59	7.95E-09
TSPAN12	14.46	13.96	4.44	3.40	6.29	5.41	3.17	1.55	3.30E-12
MEGF10	0.78	1.14	0.26	0.37	0.37	0.72	2.10	1.46	6.13E-07
P2RY2	1.89	2.28	1.08	1.08	1.82	1.15	2.04	1.45	5.49E-04
KCNA4	1.94	6.61	2.83	3.03	1.17	3.00	2.19	1.40	3.40E-15
NPAS2	1.69	2.62	1.08	1.25	1.69	1.48	1.92	1.39	4.74E-10
KAZN	2.36	3.48	1.42	1.73	0.88	1.43	2.20	1.38	1.08E-03
PITPNM1	4.96	6.03	2.97	3.19	3.07	2.77	2.02	1.35	7.61E-05
LRRC73	1.38	2.07	1.15	0.78	1.08	0.77	2.69	1.24	4.91E-03

Supplementary Table 9
KEGG pathways enriched in *CADM1* mutant-transduced cells

RNA sequencing data from H295R cells transduced with empty vector, wild-type (WT) or mutant *CADM1*. Genes with >1.5 fold increase or <0.7 fold reduction in expression in mutant *CADM1,* compared to WT, were selected for gene enrichment analysis using Database for Annotation, visualization, and Integrated Discovery (DAVID; david.ncifcrf.gov). A Modified Fisher Exact *p*-value is shown, denoting strength of enrichment of genes (observed versus expected) in the annotation categories.

Term	Count	%	P Value	Genes	List Total	Pop Hits	Pop Total	Fold Enrichment	Bonferroni	Benjamini	FDR
hsa04925:Aldosterone synthesis and secretion	9	0.0187	1.40E-04	PDE2A, CYP11B2, MC2R, CACNA1G, CAMK2D, NR4A2, NPR1, NR4A1, CACNA1C	132	81	6879	5.7904	0.0269	0.0269	0.1744
hsa04360:Axon guidance	11	0.0229	1.41E-04	NGEF, SEMA6D, UNC5B, GNAI1, CXCR4, EFNB1, SEMA7A, EFNA3, EFNA5, SLIT2, EPHB2	132	127	6879	4.5138	0.0271	0.0136	0.1755
hsa04724:Glutamatergic synapse	9	0.0187	0.0014159	TRPC1, SLC1A3, GNAI1, GRIK2, GNG11, GRIN3A, HOMER1, CACNA1C, HOMER2	132	114	6879	4.1142	0.2414	0.0880	1.7530
hsa04024:cAMP signaling pathway	11	0.0229	0.0043605	FOS, ATP1B1, GNAI1, MC2R, CAMK2D, NPR1, RAPGEF4, GABBR2, GRIN3A, CACNA1C, TSHR	132	198	6879	2.8952	0.5735	0.1919	5.3086
hsa04010:MAPK signaling pathway	12	0.0249	0.0086201	FGFR1, FOS, FGFR4, MRAS, TGFBR2, CACNA1G, NR4A1, FGF13, CACNA1C, DUSP8, MYC, TGFB2	132	253	6879	2.4718	0.8152	0.2866	10.2429
hsa04015:Rap1 signaling pathway	10	0.0208	0.018459	FGFR1, FGFR4, MAGI1, GNAI1, MRAS, CNR1, EFNA3, FGF13, EFNA5, RAPGEF4	132	210	6879	2.4816	0.9736	0.4542	20.7497
hsa05034:Alcoholism	9	0.0187	0.019389	HIST1H2BL, HIST1H2BF, GNAI1, HIST1H2BI, GNG11, HIST1H2AK, GRIN3A, HIST3H2BB, HIST1H4I	132	177	6879	2.6498	0.9780	0.4204	21.6818
hsa04713:Circadian entrainment	6	0.0125	0.0344458	FOS, GNAI1, CACNA1G, CAMK2D, GNG11, CACNA1C	132	95	6879	3.2914	0.9989	0.5745	35.4372
hsa04380:Osteoclast differentiation	7	0.0146	0.0385054	FOS, FOSL2, SOCS3, TGFBR2, SOCS1, FHL2, TGFB2	132	131	6879	2.7847	0.9995	0.5729	38.7448
hsa05166:HTLV-I infection	10	0.0208	0.0527375	E2F2, FOS, WNT4, MSX1, MRAS, CREM, TGFBR2, MYB, MYC, TGFB2	132	254	6879	2.0517	1.0000	0.6523	49.1486
hsa04930:Type II diabetes mellitus	4	0.0083	0.0627959	SOCS3, SOCS1, CACNA1G, CACNA1C	132	48	6879	4.3428	1.0000	0.6833	55.4924
hsa00330:Arginine and proline metabolism	4	0.0083	0.0692686	ARG2, CKMT2, SMS, ALDH3A2	132	50	6879	4.1691	1.0000	0.6885	59.1807
hsa04350:TGF-beta signaling pathway	5	0.0104	0.0757107	BMP4, TGFBR2, MYC, TGFB2, BMP6	132	84	6879	3.1020	1.0000	0.6930	62.5704
hsa05202:Transcriptional misregulation in cancer	7	0.0146	0.098651	TGFBR2, ETV1, NR4A3, MEIS1, MYC, ETV5, ETV4	132	167	6879	2.1844	1.0000	0.7646	72.6489

Supplementary Table 10
Primers used for CADM1 Sanger Sequencing

Genetic template	Forward Primer 5’ → 3’	Reverse primer 5’ →3’
cDNA (UK)	ATTCCCGAGCAGGTGAAGAAGG	AGTGAAGTATGTACCTTTATGTCTGGC
gDNA (UK/German)	GTGAAGAAGGCTCGATCAGG	TTCAGGAATCAGATAGGCCG
gDNA (France)	GGCTTCTGGTGCCTTGTAAT	TCTCCAAAGGCAGAAGCTGT

Supplementary Table 11
List of primary antibodies used for IHC staining of adrenal sections, including dilutions and positive control tissue used

Primary Antibody	Source/<br>Catalog #/<br>Lot #/<br>Immunogen sequence (when published)	Primary Antibody Dilution	Positive Tissue Control
Mouse monoclonal anti-CYP11B2	N/A<br>*Gift from Prof Celso Gomez-Sanchez[13]	1:100	Adrenal
Rat monoclonal anti-CYP11B1	N/A<br>*Gift from Prof Celso Gomez-Sanchez[13]	1:100	Adrenal
Rabbit polyclonal anti-CYP17A1	N/A<br>*Gift from Prof Celso Gomez-Sanchez	1:200	Testis
Rabbit polyclonal anti-KCNJ5	Source: Sigma-Aldrich<br>Catalog #: HPA017353<br>Lot #: 000004263<br>Immunogen sequence:<br>VTPWDPKKIPKQARDYVPIATDRTRLLAEGKKPRQRYMEK<br>SGKCNVHHGNVQETY	1:100	Adrenal
Rabbit polyclonal anti-GJA1 (HPA)	Source: Sigma-Aldrich<br>Catalog #: HPA035097<br>Lot #: A114990<br>Immunogen sequence:<br>EQNWANYSAEQNRMGQAGSTISNSHAQPFDFPDDNQ<br>NSKKLAAGHELQPLAIVDQRPSSRASSRASSRPR	1:100	Heart/<br>Testis
Rabbit polyclonal anti-GJA1 (C6219)	Source: Sigma-Aldrich<br>Catalog #: C6219<br>Lot #:90420<br>Immunogen sequence: KPSSRASSRASSRPRPDDLEI	1:500	Heart/<br>Testis
Rabbit polyclonal anti-TJP1	Source: Sigma-Aldrich<br>Catalog #: HPA001636<br>Lot #: 000001836<br>Immunogen sequence:<br>RKLYERSHKLRKNNHHLFTTTINLNSMNDGWYGALKEAI<br>QQQQNQLVWVSEGKADGATSDDLDLHDDRLSYLSAPGS<br>EYSMYSTDSRHTSDYEDTDTEGGAYTDQELDETLNDEVG<br>TPPESAITRSSEPVRED	1:400	Kidney
Rabbit polyclonal anti-CADM1	Source: Sigma-Aldrich<br>Catalog #: S4945<br>Lot #: 025K4823	1:3000-<br>5000	Testis
Rabbit polyclonal anti-AQP2	Source: Novus Biologicals<br>Catalog #: NB110-74682<br>Lot #: F	1:1000	Kidney

Supplementary Table 12
Definition of adrenocortical zones based on IHC scores

IHC Score	APM	ZG	ZI	ZF
CYP11B2	>2	<1	<1	<1
CYP17A1	0-3	<1	>2	>2
KCNJ5	0-3	>2	>CYP17A1	<CYP17A1

APM: aldosterone-producing micronodule, ZG: zona glomerulosa, ZI: zona intermedia, ZF: zona fasciculata.

Supplementary Table 13
List of primary and Alexa Fluor™(AF) conjugated secondary antibodies used for IFC staining of adrenal sections

Antibody	Source (Catalog #)	Lot #	Antibody Dilution
Mouse anti-CYP11B2	N/A<br>*Gift from Prof Celso Gomez-Sanchez[13]	41-13B19/19/2018	1:100
Mouse anti-CYP17A1	N/A<br>*Gift from Prof Celso Gomez-Sanchez	10-19-64-7710	1:2000
Rabbit anti-GJA1	Sigma-Aldrich (C6219)	A114990	0.5 µL/ml
Mouse anti-VSNL1	EMD Millipore (MABN762, clone 2D11)	3277598	0.5 µL/ml
Rabbit anti-DAB2	Sigma-Aldrich (HPA028888)	N/A	1:500
Goat anti-mouse antibody AF 488	Invitrogen (A-10680)	N/A	2 µg/mL
Goat anti-rabbit antibody AF 568	Invitrogen (A-11011)	N/A	2 µg/mL

Supplementary Table 14
Primers used for generation of pLOC plasmids

Name of primer	Primer Sequence (5’→ 3’)
TF-CADM1-F	CACCATGGATTACAAGGATGACGACGATAAGGGATCCATGGCGAGTGTAGTGCTG
M-CADM1-R	GCATGAATTCCTACAGATCTTCTTCGCTAATCAGTTTCTGTTCGATGAAGTACTCTTTCTTTTCTTCG
Ins-Ex9A-F	gaaccagcagttcacgATTCCCGAGCAGGTGAAG
Ins-Ex9A-R	tgtcgtcgccgttgtgtCTGTGATGATGGTAAGGATG
G379D-F	GTGATCGGTGaCGTCGTGGCG
G379D-R	GGCATGATCCACTGCCCTG
V380D-F	ATCGGTGGCGaCGTGGCGGTG
V380D-R	CACGGCATGATCCACTGCC

Supplementary Table 15
Primers used for generation of wild-type, G379D and V380D *CADM1*

	Forward primer 5’→ 3’	Reverse primer 5’→ 3’
Wild-type CADM1	agtctgaggcaggtgcccgacat	gttccaatgaggtgtccaactg
G379D CADM1	tgatcggtgacgtcgtggcggtg	caccgccacgacgtcaccgatca
V380D CADM1	tgatcggtggcgacgtggcggtg	caccgccacgtcgccaccgatca

Supplementary Table 16
Commercial Taqman Gene Expression Assays used for quantification of mRNA expression of genes of interest

Gene	Assay ID	Amplicon Length
CADM1	Hs00942509_m1	77
CYP11B2	Hs01597732_m1	137
GJA1	Hs00748445_s1	142
GJC1	Hs00748445_s1	55
LGR5	Hs00969422_m1	62
GSTA3	Hs01598344_m1	142
AQP2	HS05033967_s1	154
b-actin	Hs01060665_g1	63
18S	Hs99999901_s1	187

Source data for Supplementary Figure 2a

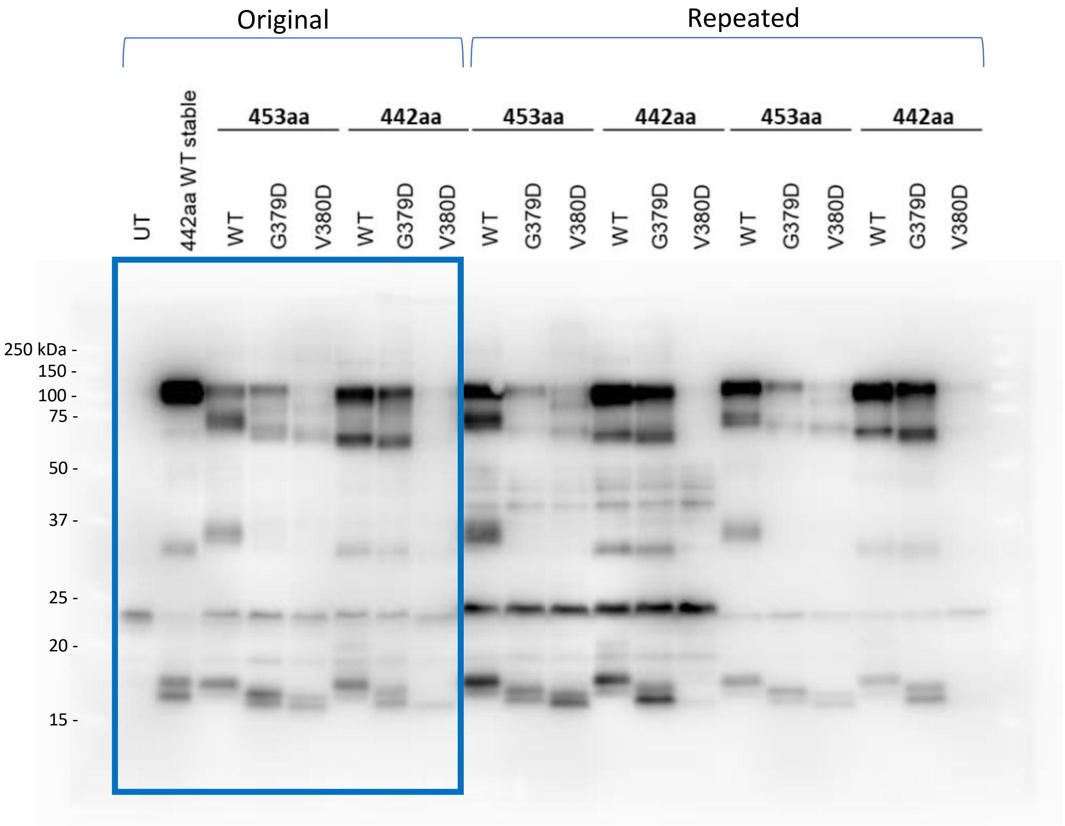

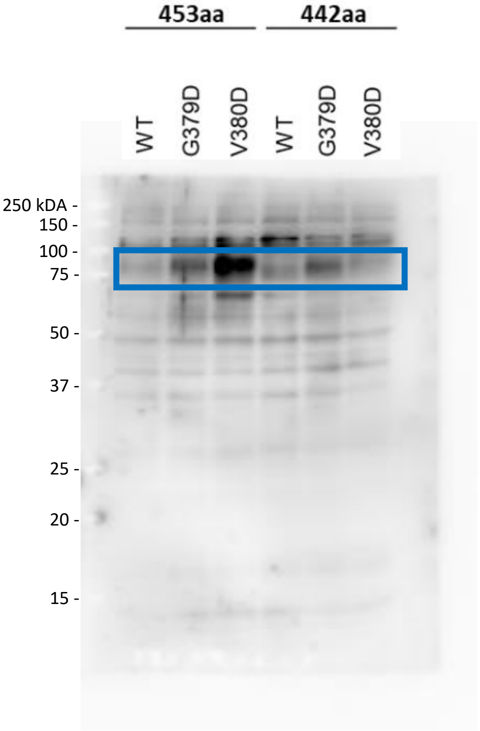

Top immunoblot show cell lysates stained using custom-made C-terminal CADM1. Bottom immunoblot show the medium supernatant from same cells stained using an N-terminal CADM1 antibody.

Areas of Western Blot cropped and shown in **Supplementary Fig. 2a** are highlighted by the blue boxes

Source data for Supplementary Figure 2b

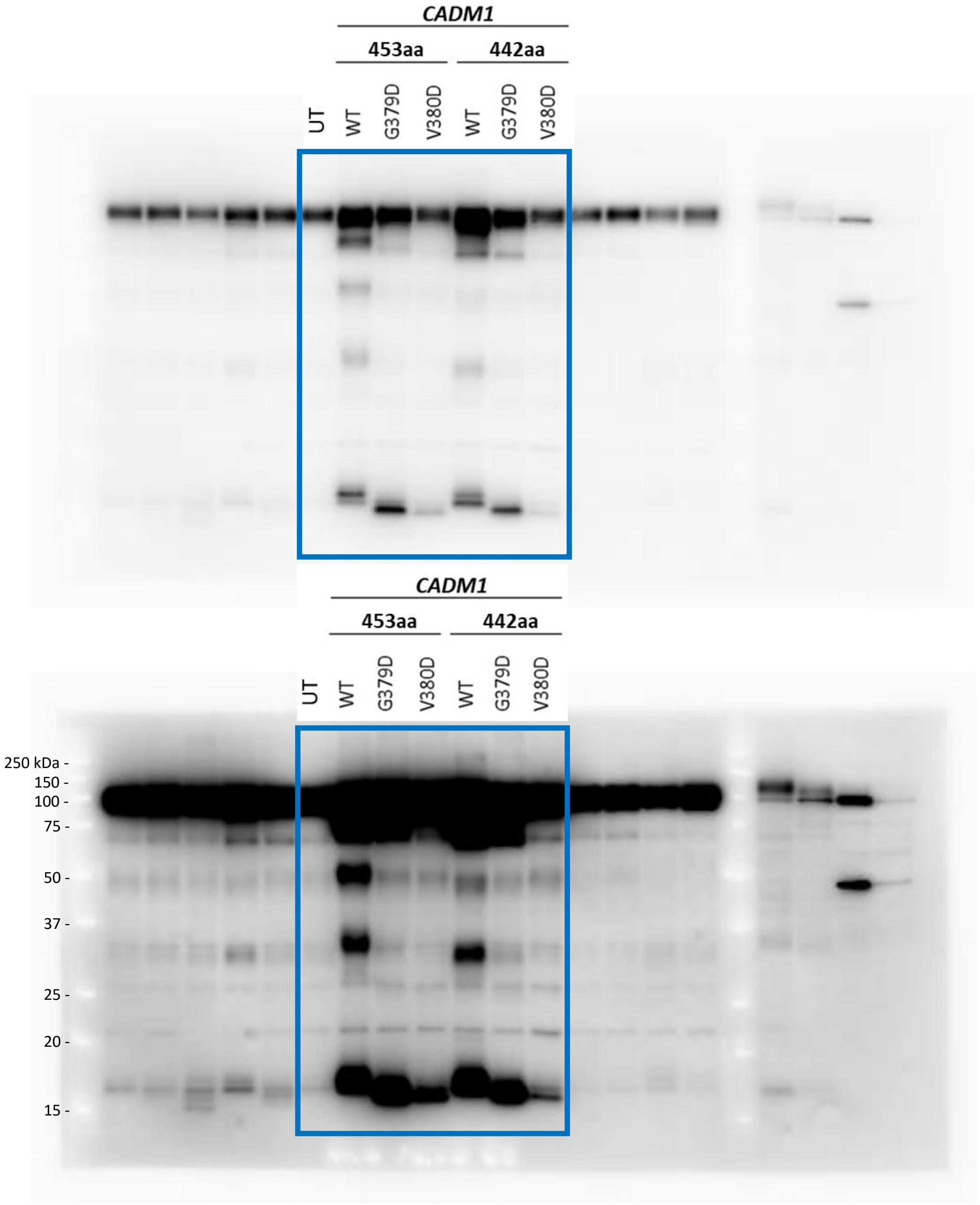

Long exposure

Very long exposure to show protein ladder

Cell lysates were immunoblotted with a custom-made anti-CADM1 C-terminal antibody.

Areas of Western Blot cropped and shown in **Supplementary Fig. 2b** are highlighted by the purple and blue boxes respectively

Source data for Supplementary Figure 2c

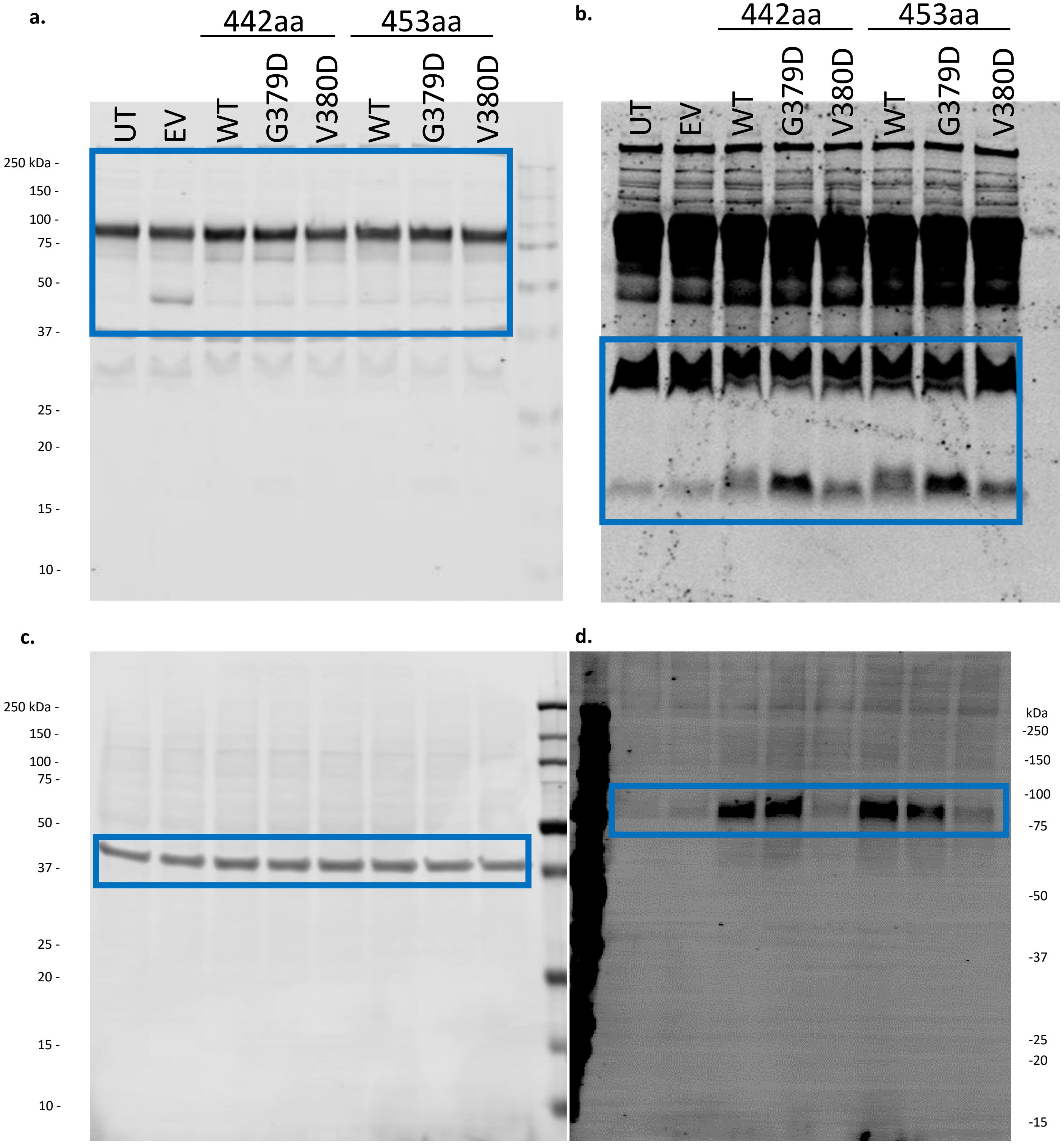

Total protein from cell lysates of transduced H295R cells with wild-typr (WT) or mutant CADM1 (G379D or V380D) were immunoblotted with **a.** c-terminal CADM1 antibody (short exposure), **b.** c-terminal CADM1 antibody (long exposure), and **c.** GAPDH. The blots were sequentially immunoblotted, after stripped of previous antibodies. **d**. The medium supernantant of same cells were immunoblotted with an n-terminal CADM1 antibody. Areas of Western Blot cropped and shown in **Supplementary Fig. 2c** are highlighted by the blue box. UT, untransduced; EV, empty vector.

Source data for Supplementary Fig. 8c

a.

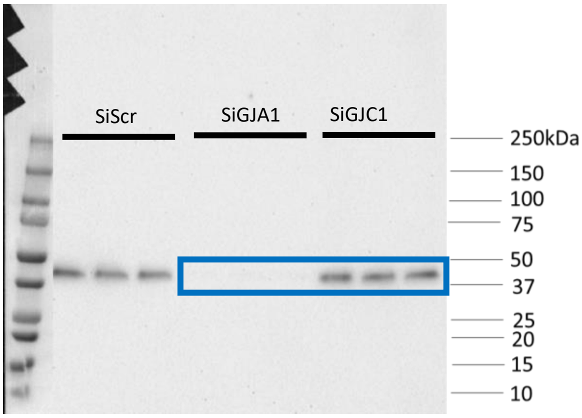

b.

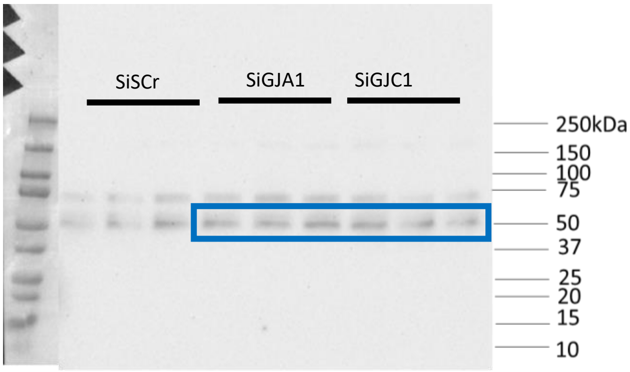

c.

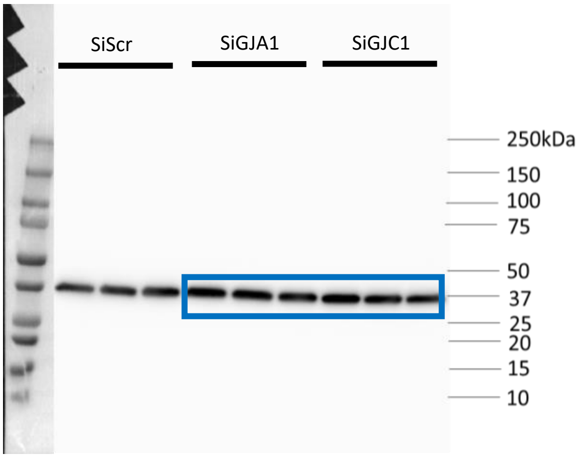

Total protein from cell lysates of single-silenced (using either SiGJA1 or SJC1) H295R cells were immunoblotted for **a.** GJA1 and **b.** GJC1. **c.** GAPDH. The blots were sequentially immunoblotted, after stripped of previous antibodies. Areas of Western Blot cropped and shown in **Supplementary Fig. 8c** are highlighted by the blue box.

Source data for Supplementary Fig. 8f

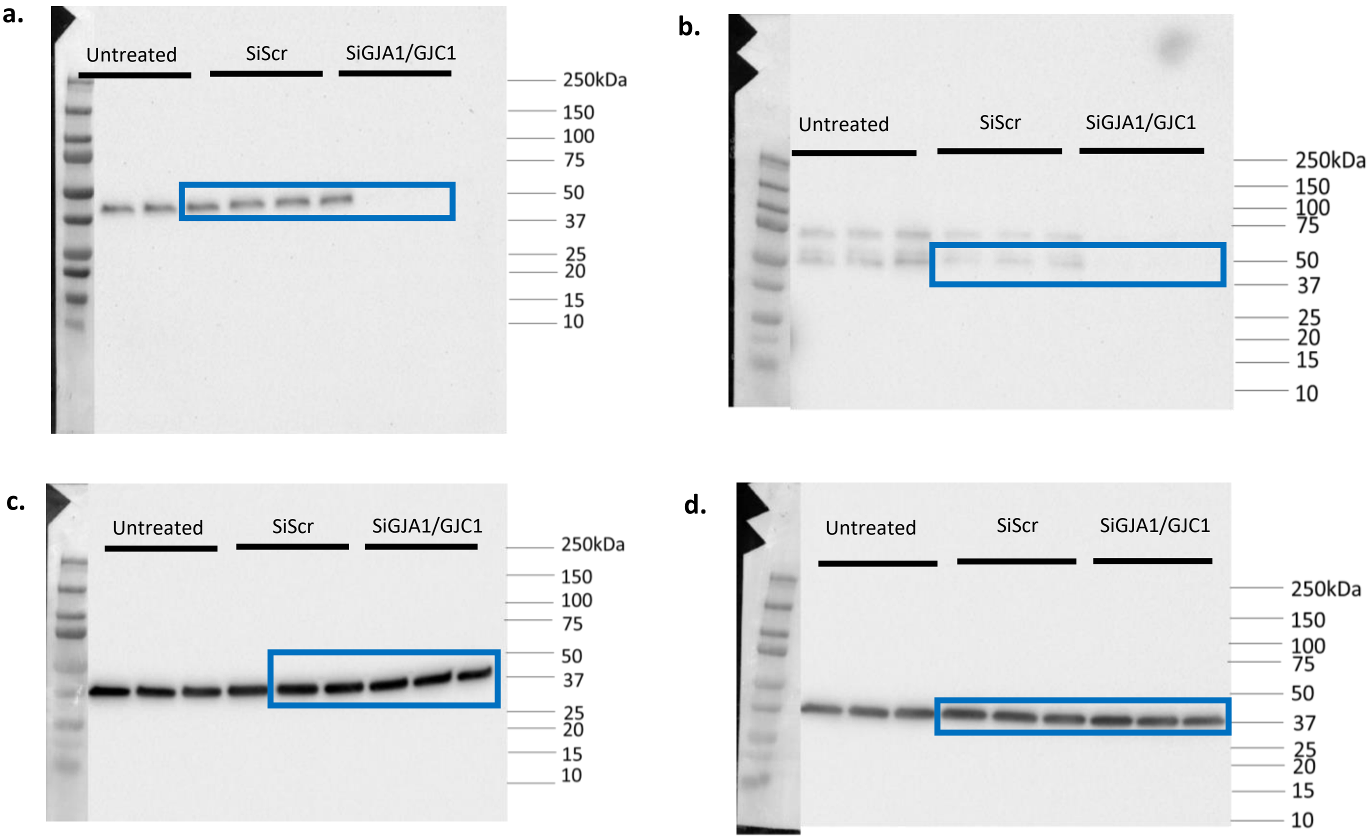

Total protein from cell lysates of co-silenced (SiGJA1/SJC1) H295R cells were immunoblotted for **a.** GJA1 and **b.** GJC1. **c-d.** These blots were further stripped and immunoblotted for GAPDH. Areas of Western Blot cropped and shown in **Supplementary Fig. 8f** are highlighted by the blue box. Samples were derived from the same experiment and the gels/blots were processed in parallel.

Supplementary Note

Results

Calcium Oscillations

Studies in the pancreas suggest GJs coordinate intracellular calcium oscillations in islet cells[1]. Their inhibition increases basal insulin secretion but prevents pulsatile secretion in response to glucose[2]. Calcium oscillations are well recognized in mouse adrenal ZG to regulate aldosterone production[3-5], coordinated within rosettes of cells[6]. Inhibition of GJs with Gap27 did not affect oscillations of individual H295R adrenocortical cells, which (in mouse ZG) are transmitted by adherens junctions[6]; however, Gap27 increased mean fluorescence intensity of cells at baseline (**Supplementary Fig. 9a-c**). The increase was similar to that caused by angiotensin-II, but Gap27 did not significantly augment the effect of angiotensin II (**Supplementary Fig. 9d**). Many small-molecule mediators pass through GJs and are candidates for controlling calcium release from intracellular stores[7,8].

Silencing of CADM1 in H295R cells

The most downregulated gene, by 16-fold, secretogranin 3 (*SCG3*) shares 46% identity, organ-distribution and function with the most upregulated on mutation, *SCG2*. However, most of the other large changes were in the same direction in the two microarrays, except for the adrenal peroxisome target of fibrates, *ALDH3A2* (**Fig. 6b**, **Supplementary Fig. 10** and **Supplementary Table 6b**). Germline inactivation of *ALDH3A2* was recently reported in adrenal hypoplasia[9].

Discussion

Result anomalies

Two anomalies required explanation. First, both silencing and mutation of CADM1 inhibited GJ communication, but had opposite effects on aldosterone production. The clue to resolving this anomaly came from the study of *FGFR3* mutations, which in addition to their effects on ectodomain orientation constitutively recapitulate the intracellular kinase activation by ligand binding and dimerization[44,10]. Similarly, the intercellular adhesion of CADM1 ectodomains is anticipated to have two roles. One is extracellular, concerning communication between cells, and the other involves intracellular actions via its C-terminus. It is likely that loss of both the C-terminus and N-terminus of CADM1 on silencing, compared to activation of the cytosolic tail region by intact CADM1 dimers, underlies the opposite effects of silencing and mutation on aldosterone production.

The other anomaly was the sparsity of gap junctions in ZG[11]. Although they have previously been seen on electron microscopy, and connections between adrenomedullary and ZG cells are also described, their reported abundance was clearly less than in other adrenal zones[12,13]. Herein, extensive RNA and protein analyses confirmed expression of GJA1 protein in human ZG, probably more than in other species. Furthermore, detection of annular GJs infers at least transient formation of intact GJs, since annular GJs require GJA1 proteins from both the communicating cells. Their shorter half-life than most membrane proteins[14,15] may contribute to the discrepancy between abundance of *GJA1* mRNA and sparsity of protein in ZG[11-13]. Semi-quantitation showed inverse correlation between sites of CYP11B2 and GJA1 expression, whereas CYP17A1 (required for cortisol synthesis) and GJA1 were positively correlated. Indeed, the rarity of GJs in APMs resembles the picture in rodent ZG, so a greater importance of GJIC in human than rodent ZG is consistent with the dominance of inhibitory pathways in adult human ZG[16-21]. In other tissues, e.g. sinus node of the heart and olfactory bulb, small or sparse GJs play an important role in their physiology[22,23].

Aquaporin expression in CADM1 mutant APAs

Several aquaporins (AQP1, AQP2, AQP5) have been reported in secretory vesicle membranes and shown to be involved in gating of water into the vesicles, swelling on secretion, or trafficking to

plasma membranes[24,25-28]. The same aquaporins show circadian variation in mouse (AQP1 and AQP5) (http://circadb.hogeneschlab.org)[29] or human tissues (AQP2)[30]. Traditionally, steroid secretion is considered a passive process of lipid-soluble molecules diffusing across the plasma membrane, but in the setting of 10-20 fold increases in CYP11B2 expression, this may need re-examination.

References

1. Benninger, R.K., Head, W.S., Zhang, M., Satin, L.S. & Piston, D.W. Gap junctions and other mechanisms of cell-cell communication regulate basal insulin secretion in the pancreatic islet. *J Physiol* **589**, 5453-66 (2011).
2. Ravier, M.A. *et al.* Loss of connexin36 channels alters beta-cell coupling, islet synchronization of glucose-induced Ca2+ and insulin oscillations, and basal insulin release. *Diabetes* **54**, 1798-807 (2005).
3. Spät, A., Enyedi, P., Hajnóczky, G. & Hunyady, L. Generation and role of calcium signal in adrenal glomerulosa cells. *Exp Physiol* **76**, 859-85 (1991).
4. Barrett, P.Q. *et al.* Role of voltage-gated calcium channels in the regulation of aldosterone production from zona glomerulosa cells of the adrenal cortex. *J Physiol* **594**, 5851-5860 (2016).
5. Penton, D. *et al.* Task3 potassium channel gene invalidation causes low renin and salt-sensitive arterial hypertension. *Endocrinology* **153**, 4740-8 (2012).
6. Guagliardo, N.A. *et al.* Angiotensin II induces coordinated calcium bursts in aldosterone-producing adrenal rosettes. *Nat Commun* **11**, 1679 (2020).
7. Murray, S.A., Davis, K. & Gay, V. ACTH and adrenocortical gap junctions. *Microsc Res Tech* **61**, 240-6 (2003).
8. Spat, A., Hunyady, L. & Szanda, G. Signaling Interactions in the Adrenal Cortex. *Front Endocrinol (Lausanne)* **7**, 17 (2016).
9. Abu Diab, A. *et al.* The combination of whole-exome sequencing and clinical analysis allows better diagnosis of rare syndromic retinal dystrophies. *Acta Ophthalmol* **97**, e877-e886 (2019).
10. Li, E., You, M. & Hristova, K. FGFR3 dimer stabilization due to a single amino acid pathogenic mutation. *J Mol Biol* **356**, 600-12 (2006).
11. Murray, S.A., Nickel, B.M. & Gay, V.L. Gap junctions as modulators of adrenal cortical cell proliferation and steroidogenesis. *Mol Cell Endocrinol* **300**, 51-6 (2009).
12. Bornstein, S.R., Ehrhart-Bornstein, M. & Scherbaum, W.A. Morphological and functional studies of the paracrine interaction between cortex and medulla in the adrenal gland. *Microsc Res Tech* **36**, 520-33 (1997).
13. Palacios, G. Cell junctions in the adrenal cortex of the postnatal rat. *J Anat* **129**, 695-701 (1979).
14. Solan, J.L. & Lampe, P.D. Specific Cx43 phosphorylation events regulate gap junction turnover in vivo. *FEBS Lett* **588**, 1423-9 (2014).
15. Segretain, D. & Falk, M.M. Regulation of connexin biosynthesis, assembly, gap junction formation, and removal. *Biochimica et Biophysica Acta (BBA) - Biomembranes* **1662**, 3-21 (2004).
16. Shaikh, L.H. *et al.* LGR5 Activates Noncanonical Wnt Signaling and Inhibits Aldosterone Production in the Human Adrenal. *J Clin Endocrinol Metab* **100**, E836-44 (2015).
17. Zhou, J. *et al.* Transcriptome Pathway Analysis of Pathological and Physiological Aldosterone-Producing Human Tissues. *Hypertension* **68**, 1424-1431 (2016).

18. Maniero, C. *et al.* NEFM (Neurofilament Medium) Polypeptide, a Marker for Zona Glomerulosa Cells in Human Adrenal, Inhibits D1R (Dopamine D1 Receptor)-Mediated Secretion of Aldosterone. *Hypertension* **70**, 357-364 (2017).
19. Maniero, C. *et al.* ANO4 (Anoctamin 4) Is a Novel Marker of Zona Glomerulosa That Regulates Stimulated Aldosterone Secretion. *Hypertension* **74**, 1152-1159 (2019).
20. Gomez-Sanchez, C.E. *et al.* Development of monoclonal antibodies against human CYP11B1 and CYP11B2. *Mol Cell Endocrinol* **383**, 111-7 (2014).
21. Nishimoto, K. *et al.* Adrenocortical Zonation in Humans under Normal and Pathological Conditions. *The Journal of Clinical Endocrinology & Metabolism* **95**, 2296-2305 (2010).
22. Pouille, F., McTavish, T.S., Hunter, L.E., Restrepo, D. & Schoppa, N.E. Intraglomerular gap junctions enhance interglomerular synchrony in a sparsely connected olfactory bulb network. *J Physiol* **595**, 5965-5986 (2017).
23. Davis, L.M., Rodefeld, M.E., Green, K., Beyer, E.C. & Saffitz, J.E. Gap junction protein phenotypes of the human heart and conduction system. *J Cardiovasc Electrophysiol* **6**, 813-22 (1995).
24. Jena, B.P. Aquaporin regulation: Lessons from secretory vesicles. *Vitam Horm* **112**, 147-162 (2020).
25. Arnaoutova, I. *et al.* Aquaporin 1 is important for maintaining secretory granule biogenesis in endocrine cells. *Mol Endocrinol* **22**, 1924-34 (2008).
26. Cho, S.J. *et al.* Aquaporin 1 regulates GTP-induced rapid gating of water in secretory vesicles. *Proc Natl Acad Sci U S A* **99**, 4720-4 (2002).
27. Roche, J.V., Nesverova, V., Olsson, C., Deen, P.M. & Tornroth-Horsefield, S. Structural Insights into AQP2 Targeting to Multivesicular Bodies. *Int J Mol Sci* **20**(2019).
28. Noda, Y. & Sasaki, S. The role of actin remodeling in the trafficking of intracellular vesicles, transporters, and channels: focusing on aquaporin-2. *Pflugers Arch* **456**, 737-45 (2008).
29. Hogenesch, J. CircaDB.
30. Zuber, A.M. *et al.* Molecular clock is involved in predictive circadian adjustment of renal function. *Proc Natl Acad Sci U S A* **106**, 16523-8 (2009).
