# Supplementary material for: Somatic mutations of CADM1 in aldosterone-producing adenomas and gap junction-dependent regulation of aldosterone production
Source: Nat Genet. 2023 Jun 8;55(6):1009–21. doi: 10.1038/s41588-023-01403-0 (PMC10260400; doi:10.1038/s41588-023-01403-0)
Supplement: Supplementary file 2 — Reporting Summary [file 41588_2023_1403_MOESM2_ESM.pdf]

nature research

Corresponding author(s): Elena A.B. Azizan
Morris J. Brown

Last updated by author(s): 12/12/2022

Reporting Summary

Nature Research wishes to improve the reproducibility of the work that we publish. This form provides structure for consistency and transparency in reporting. For further information on Nature Research policies, see our Editorial Policies and the Editorial Policy Checklist.

Statistics

For all statistical analyses, confirm that the following items are present in the figure legend, table legend, main text, or Methods section.

n/a	Confirmed	
☐	☒	The exact sample size (*n*) for each experimental group/condition, given as a discrete number and unit of measurement
☐	☒	A statement on whether measurements were taken from distinct samples or whether the same sample was measured repeatedly
☐	☒	The statistical test(s) used AND whether they are one- or two-sided<br>*Only common tests should be described solely by name; describe more complex techniques in the Methods section.*
☐	☒	A description of all covariates tested
☐	☒	A description of any assumptions or corrections, such as tests of normality and adjustment for multiple comparisons
☐	☒	A full description of the statistical parameters including central tendency (e.g. means) or other basic estimates (e.g. regression coefficient) AND variation (e.g. standard deviation) or associated estimates of uncertainty (e.g. confidence intervals)
☐	☒	For null hypothesis testing, the test statistic (e.g. *F*, *t*, *r*) with confidence intervals, effect sizes, degrees of freedom and *P* value noted<br>*Give P values as exact values whenever suitable.*
☒	☐	For Bayesian analysis, information on the choice of priors and Markov chain Monte Carlo settings
☒	☐	For hierarchical and complex designs, identification of the appropriate level for tests and full reporting of outcomes
☒	☐	Estimates of effect sizes (e.g. Cohen's *d*, Pearson's *r*), indicating how they were calculated

Our web collection on statistics for biologists contains articles on many of the points above.

Software and code

Policy information about availability of computer code

Data collection	3DHISTECH Pannaromic MIDI scanner Software v1.18 (3D HISTCH, Hungary) for IHC slide image acquisition<br>CaseViewer Software v2.4 for IHC slide image acquisition<br>Vectra® automated imaging system software v.3.0.5 (Perkin Elmer) for IHC slide image acquisition<br><br>TissueFAXS SL Q+ upright epifluorescence microscope and viewed with TissueFAXS slide viewer 7.0 software for IFC image acquisition<br><br>NIS-Elements v4.5 (Nikon, Japan): fluorescence and transmitted light microscopy image acquisition<br><br>FLUOstar Omega series v5.7 (BMG Labtech, UK): Homogeneous Time Resolved Fluorescence 24 (HTRF) aldosterone assay acquisition<br><br>BD FACSDiva Software v6.1.3 (BD Biosciences, USA): Fluorescence-activated cell sorting (FACS) for lentivirus titration<br><br>CFX Manage TM Software v3.1 (Bio-Rad, USA) or 7900 SDS v2.4.1 (Applied Biosystems, USA): RT-PCR data acquisition<br><br>NextSeq 500 high-output 150 cycle kit v2.5 and bcl2fastq Conversion Software v1.8.4: RNA sequencing
Data analysis	For protein structure modelling: QUARK March 2018 (https://zhanglab.ccmb.med.umich.edu/QUARK/) and GROMACs (2019 release) was used for structure analysis of the transmembrane domains and intercellular domains of wild-type and mutant CADM1 because no template structure of these domains for homology modelling were available. PyMOL v.2.1 (Schrödinger, LLC) for visualization of the CADM1 structures. ZDOCK v.3.0.2 (http://zdock.umassmed.edu/) was used for docking analysis for the dimerization and tetramerization of the CADM1.<br>The proteins structure of the transmembrane domain of CADM1 was also separately modelled using Phyre2 v2 (http://www.sbg.bio.ic.ac.uk/~phyre2/html/page.cgi?id=index) and TMDOCK (https://membranome.org/tmdock) was used to predict the insertion and homodimerization

of the transmembrane region of the wild type and Phyre predicted mutant (G379D and V380D) structures.
Statistical analysis was performed using R software, R Core Team. R (v.3.5.0) was used for one-way ANOVA followed by Tukey's post hoc tests (ptukey function of R).

GATC Viewer v.1.00 or UniPro UGENE v.1.28.1 for Sanger sequencing alignment

Flowing Software v.2.5.1 for lentivirus titration using FACS

Microsoft Excel 2016 for HTRF aldosterone assay and qPCR (2-ΔΔCT) calculations

Fiji ImageJ v.1.52p Java 1.8.0_172 for western blot quantification
FIJI ImageJ software v1.8.0_72 was used to prepare the IF images for presentation

Prism (Graphpad software v.8.4.3 or later) for statistical analysis for aldosterone, qPCR, western blot and calcium oscillation data.

Partek Flow software (Partek, St. Louis, Missouri, United States), including its annotation tool and GSA tool was used for RNA sequencing analysis. STAR -2.6.1d was used for sequence alignment and DAVID Bioinformatics Resources v.6.8 (https://david.ncifcrf.gov/tools.jsp) for gene enrichment analysis.

For manuscripts utilizing custom algorithms or software that are central to the research but not yet described in published literature, software must be made available to editors and reviewers. We strongly encourage code deposition in a community repository (e.g. GitHub). See the Nature Research guidelines for submitting code & software for further information.

Data

Policy information about availability of data

All manuscripts must include a data availability statement. This statement should provide the following information, where applicable:

- Accession codes, unique identifiers, or web links for publicly available datasets
- A list of figures that have associated raw data
- A description of any restrictions on data availability

The RNAseq dataset used to generate Fig. 6, Supplementary Fig. 10, Supplementary Table 5b, Supplementary Tables 6-9 is shown in Supplementary Data 1-3. The WES raw data is publicly available from the Sequence Read Archive (https://www.ncbi.nlm.nih.gov/sra/docs/) under accession nos. PRJNA732946 and PRJNA729738. Source data for Figure 2c-e, Figure 4b, Figure 4d, Figure 5b-d, Extended Figure 9a-b, Supplementary Figure 4a-b, Supplementary Figure 5d, Supplementary Figure 8a-b, Supplementary Figure 8d-e, Supplementary Figure 8g-h, Supplementary Figure 9c-d is provided with the paper.

Field-specific reporting

Please select the one below that is the best fit for your research. If you are not sure, read the appropriate sections before making your selection.

☒ Life sciences ☐ Behavioural & social sciences ☐ Ecological, evolutionary & environmental sciences

For a reference copy of the document with all sections, see nature.com/documents/nr-reporting-summary-flat.pdf

Life sciences study design

All studies must disclose on these points even when the disclosure is negative.

Sample size	Sample size used in experiments were determined by pilot studies which generally indicated that n values of minimum of 3 was required to give a significant result. The following criteria were also used to pre-determined sample size:<br>Consistency in experimental replicates performed.<br>Consistency in difference between isoforms and mutations detected, compared to wild-type or untreated cells.<br>A minimum of 2 independent results to represent experimental variability (number specified in figure legends or methods section).<br>For dye transfer assays, all cells within a 50 um radius of the original cell of interest were included in the analysis.<br>For quantification of gap junction formation in H295R cells, cells within each well were systemically imaged. All successfully transfected cells were accounted for to limit bias.<br>For calcium oscillation experiments, ROIs were determined for all cells in the image field. For quantification the condition with the fewest numbers of cells in a single image field across all experiments was used to determine the sample size (44-46). The 44-46 cells selected for quantification in all other conditions were done so at random.
Data exclusions	No data that passed quality control were excluded.
Replication	All experiments were repeated, a minimum two times, with independent cohorts of biological samples. All attempts at replication was successful and all experimental findings were reliably reproduced.
Randomization	Randomization is not relevant to our study because experimental groups are determined by genotype.
Blinding	Investigators were not blinded to experimental group as data collection and quantification was objective and not impacted by investigators presumptions.

Reporting for specific materials, systems and methods

We require information from authors about some types of materials, experimental systems and methods used in many studies. Here, indicate whether each material, system or method listed is relevant to your study. If you are not sure if a list item applies to your research, read the appropriate section before selecting a response.

Materials & experimental systems

n/a	Involved in the study
☐	☒ Antibodies
☐	☒ Eukaryotic cell lines
☒	☐ Palaeontology and archaeology
☒	☐ Animals and other organisms
☐	☒ Human research participants
☒	☐ Clinical data
☒	☐ Dual use research of concern

Methods

n/a	Involved in the study
☒	☐ ChIP-seq
☒	☐ Flow cytometry
☒	☐ MRI-based neuroimaging

Antibodies

Antibodies used

Primary antibodies used
CADM1 C-terminal (polyclonal), 1:5000, Sigma-Aldrich, S4945
CADM1 N-terminal (3E1), MBL, CM004-3
β-actin (6D1), dilution 1:2000, Medical & Biological Laboratories (MBL), PM053
GAPDH (GAPDH-71.1), dilution 1:40,000, Sigma-Aldrich, G8795
GJA1 (polyclonal), dilution 1:8,000 for Western Blot, dilution 1:500 for IHC, Sigma-Aldrich, C6219
GJA1 (polyclonal), dilution 1:100 (for IHC), Sigma-Aldrich, HPA035097
GJC1 (polyclonal), dilution 1:2,500, Invitrogen, PA5-79311
KCNJ5 (polyclonal), dilution 1:100, Sigma-Aldrich, HPA017353
TJP1 (polyclonal), dilution 1:400, Sigma-Aldrich, HPA001636
VSNL1 (clone 2D11), 0.5 uL/ml, EMD Millipore, MABN762
DAB2 (polyclonal), dilution 1:500, Atlas Antibodies, HPA028888
Wheat germ agglutinin, Alexa FluorTM (AF) 647 conjugate, 5ul/ml, Invitrogen, W32466
CYP17A1 (polyclonal), dilution 1:2,000 (10-19-64-7710 mouse IgG 25), kindly gifted from Professor Celso E Gomez-Sanchez (The University of Mississippi, USA)
Custom made antibodies to CYP11B2 (mouse mAb) [1], dilution 1:100 (41-13B19/19/2018), kindly gifted from Professor Celso E Gomez-Sanchez (The University of Mississippi, USA)
Custom made rabbit polyclonal anti-CADM1 antibody [2], dilution 1:2,000

Secondary antibodies used
Anti-rabbit (polyclonal), Sigma-Aldrich, A0545
Anti-chicken (polyclonal), MBL, PM010-7
Anti-mouse (H&L), Vector Laboratories, BA-9200
Anti-mouse 488, Invitrogen, A-10680
Anti-rabbit AF 568 (IgG), Invitrogen, A-11011

Validation

Antibodies were selected based on previous experience of the investigators and their use in the literature on human cell lines. Antibodies previously not used by the investigators were validated using negative +/- peptide controls.

All commercial antibodies used have been validated in previously published studies as listed in their manufactures' websites, unless otherwise stated:
CADM1 C-terminal https://www.sigmaaldrich.com/catalog/product/sigma/s4945?lang=en&region=GB
CADM1 N-terminal https://www.mblbio.com/bio/g/dtl/A/index.html?pcd=CM004-3
tGFP https://www.origene.com/catalog/antibodies/tag-antibodies/ta150041/mouse-monoclonal-turbogfp-antibody-clone-oti2h8
EGFP https://www.abcam.com/gfp-antibody-ab5450.html
β-actin https://ruo.mbl.co.jp/bio/e/dtl/A/?pcd=M177-3
β-actin https://www.sigmaaldrich.com/catalog/product/sigma/a2066?lang=en&region=GB
GAPDH https://www.sigmaaldrich.com/catalog/product/sigma/g8795?lang=en&region=GB
Connexin-43 (C6219) https://www.sigmaaldrich.com/catalog/product/sigma/c6219?lang=en&region=GB
Connexin-43 (HPA035097) https://www.sigmaaldrich.com/catalog/product/sigma/hpa035097?lang=en&region=GB
Connexin-45 No citations are available on manufactures' website. For Western blot validation, five different CX-45 antibodies were validated in this study by comparing protein expression in two cell lines (HEK293T and H295R) with known high endogenous expression of CX-45, and in silenced cells. The results were correlated with mRNA expression. Only one antibody (selected) was effective, although yielded non-specific bands.
KCNJ5 https://www.sigmaaldrich.com/catalog/product/sigma/hpa017353?ang=en&region=GB&gclid=Cj0KCQiA0-6ABhDMARIsAFVdQv-y-FwndiyP5lk_zuNbGJ7f9xfjQ8C6TNydycq4Jia22jqYUtWDcQYaAslWEALw_wcB
TJP1 https://www.sigmaaldrich.com/catalog/product/sigma/hpa001636?lang=en&region=GB
VSNL1 https://www.merckmillipore.com/GB/en/product/Anti-VSNL1-Antibody-clone-2D11,MM_NF-MABN762
DAB2 https://www.atlasantibodies.com/products/antibodies/primary-antibodies/triple-a-polyclonals/dab2-antibody-hpa028888/

Custom made antibodies to CYP11B2 [1] and CADM1 [2] were validated using negative controls. The CYP11B2 antibody has been extensively cited, including [3-5]. Citations for CADM1 antibody include [6-8], for CYP17A1 include [9].

References:
1. Gomez-Sanchez, C.E. et al. Development of monoclonal antibodies against human CYP11B1 and CYP11B2. Mol Cell Endocrinol 383, 111-7 (2014).
2. Furuno, T. et al. The spermatogenic Ig superfamily/synaptic cell adhesion molecule mast-cell adhesion molecule promotes interaction with nerves. J Immunol 174, 6934-42 (2005).
3. Azizan, E.A. et al. Somatic mutations in ATP1A1 and CACNA1D underlie a common subtype of adrenal hypertension. Nat Genet 45, 1055-60 (2013).
4. Nishimoto, K. et al. Immunohistochemistry of aldosterone synthase leads the way to the pathogenesis of primary aldosteronism. Molecular and Cellular Endocrinology 441, 124-133 (2017).
5. Nishimoto, K. et al. Adrenocortical Zonation in Humans under Normal and Pathological Conditions. The Journal of Clinical Endocrinology & Metabolism 95, 2296-2305 (2010).
6. Hagiyama, M., Ichiyanagi, N., Kimura, K.B., Murakami, Y. & Ito, A. Expression of a soluble isoform of cell adhesion molecule 1 in the brain and its involvement in directional neurite outgrowth. Am J Pathol 174, 2278-89 (2009).
7. Mimae, T. et al. Increased ectodomain shedding of lung epithelial cell adhesion molecule 1 as a cause of increased alveolar cell apoptosis in emphysema. Thorax 69, 223-31 (2014).
8. Koma, Y. et al. Cell adhesion molecule 1 is a novel pancreatic-islet cell adhesion molecule that mediates nerve-islet cell interactions. Gastroenterology 134, 1544-54 (2008).
9. Gomez-Sanchez, Celso E, and Elise P Gomez-Sanchez. “Immunohistochemistry of the adrenal in primary aldosteronism.” Current opinion in endocrinology, diabetes, and obesity vol. 23,242-8 (2016)

Eukaryotic cell lines

Policy information about cell lines

Cell line source(s)	H295R cells were purchased from ECACC. NIH-3T3 cells were purchased from ATCC. Hek293T cells were a gift from Professor Xiao’s lab, William Harvey Research Institute, Queen Mary University of London, originally form ATCC.
Authentication	The cell lines used were not authenticated.
Mycoplasma contamination	The cell lines were not tested for mycoplasma contamination
Commonly misidentified lines (See ICLAC register)	No commonly misidentified cell lines were used in the study

Human research participants

Policy information about studies involving human research participants

Population characteristics	The human research participants were all adults with a diagnosis of Primary Aldosteronism who proceeded to unilateral adrenalectomy. Diagnosis, investigations and decision for surgery were made in accordance with local institutional guidelines. The demographics of the cohort in which WES was performed is detailed in the Supplementary Table 1
Recruitment	Participants were all recruited from secondary or tertiary care settings, at the hospital where adrenalectomies were being performed. All patients undergoing adrenalectomy at the specified sites were offered the opportunity to participate, with adrenal tissue collection and genetic analysis performed only in those who gave written informed consent. Potential selection bias includes willingness of patients to be enrolled into the study and the availability of adrenal tumor tissue with DNA suitable for WES.
Ethics oversight	Consent for adrenal tissue collection and genetic investigations were taken in accordance with local institutional guidelines and approved by the local ethics committee. The individual ethics committees for each centre are:<br>Cambridgeshire Research Ethics Committee for Addenbrooke’s Hospital, University of Cambridge, UK;<br>The Ethics Committee of University Hospital Munich, Germany;<br>Cambridge East Research Ethics Committee for St Bartholomew’s Hospital, Queen Mary University of London, UK;<br>Assistance Publique-Hôpitaux de Paris Research Ethics Committee, Paris, France;<br>The Institutional Review Board for Tohoku University Hospital, Sendai, Japan;<br>The Ethics Committee of Hiroshima University, for Graduate School of Biomedical and Health Sciences, Hiroshima University, Hiroshima, Japan;<br>Institutional review board, University of Pennsylvania, Philadelphia, United States of America;<br>University Hospital Hradec Kralove Ethics Committee, Czech Republic;<br>National University of Malaysia Research Ethics Committee, Malaysia.

Note that full information on the approval of the study protocol must also be provided in the manuscript.
