# Supplementary material for: Somatic mutations of CADM1 in aldosterone-producing adenomas and gap junction-dependent regulation of aldosterone production
Source: Nat Genet. 2023 Jun 8;55(6):1009–21. doi: 10.1038/s41588-023-01403-0 (PMC10260400; doi:10.1038/s41588-023-01403-0)
Supplement: Source Data Fig. 2 — Full-length blots. [file 41588_2023_1403_MOESM9_ESM.pdf]

Source data for Figure 2b

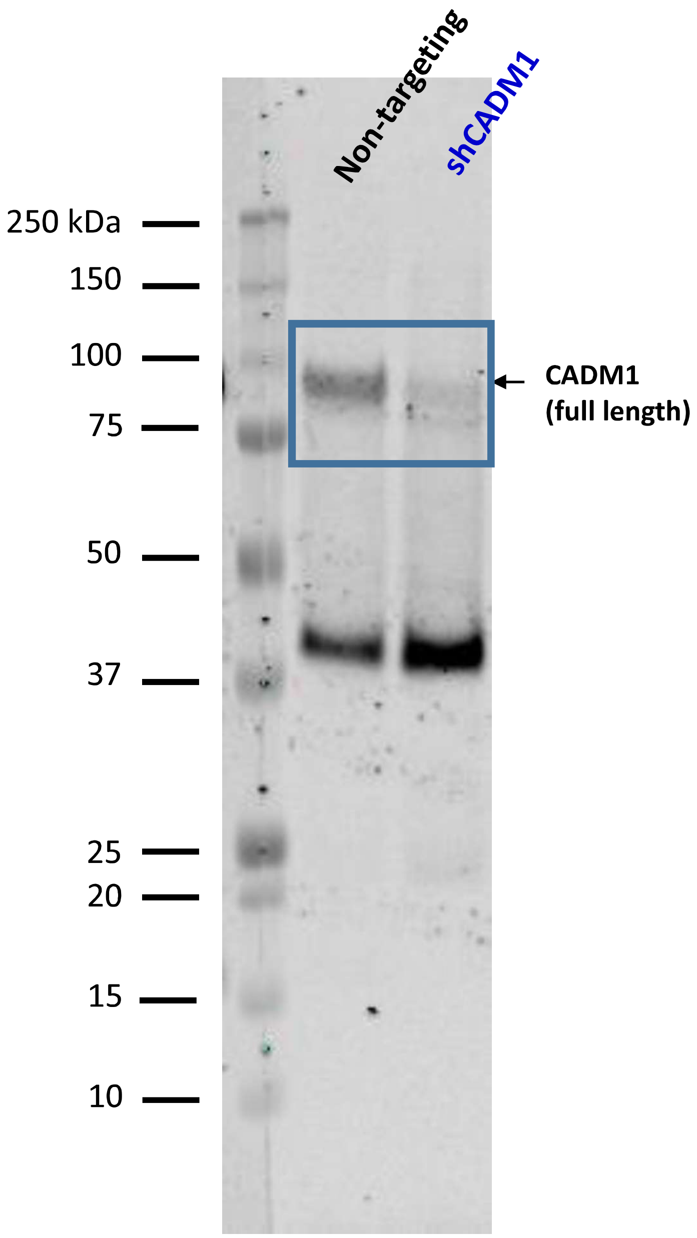

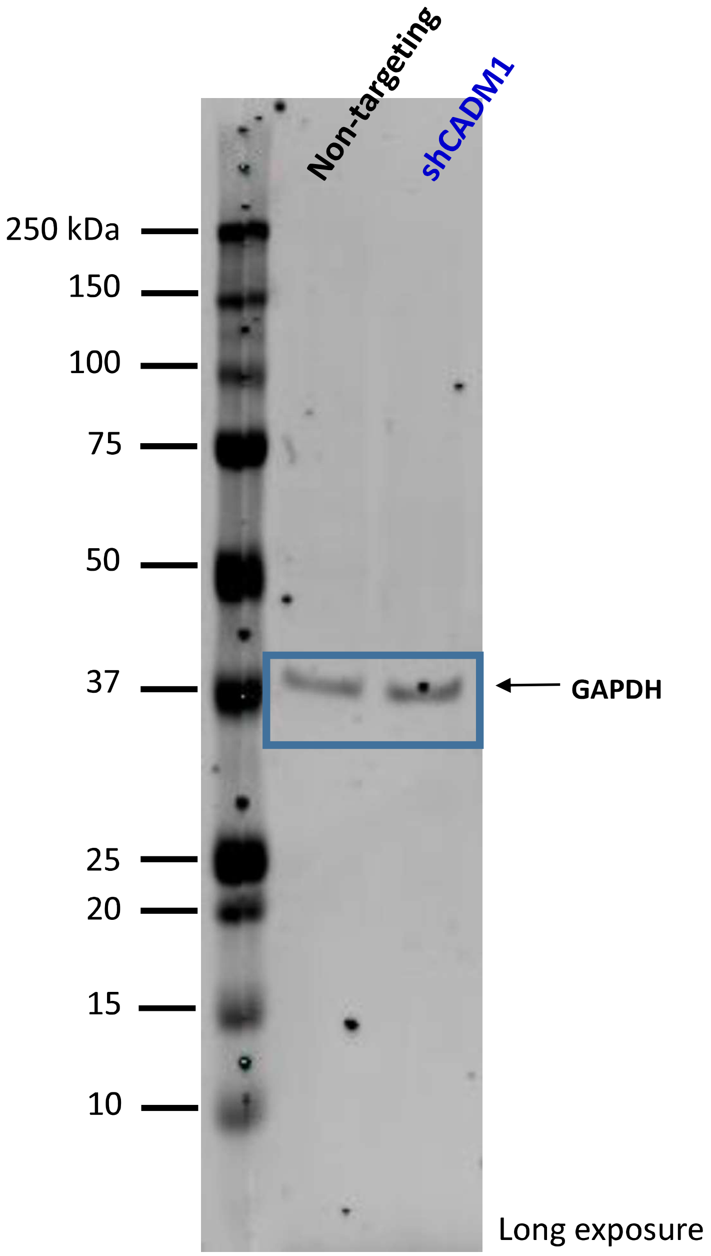

Total protein from cell lysates of transduced H295R cells with shRNA either non-targeting or targeting CADM1 (shCADM1) were immunoblotted for CADM1 (Left blot) and GAPDH (Right blot). The blots were sequentially immunoblotted, after stripped of previous antibodies. Areas of Western Blot cropped and shown in **Fig. 2b** are highlighted by the blue box.
