# Supplementary material for: Somatic mutations of CADM1 in aldosterone-producing adenomas and gap junction-dependent regulation of aldosterone production
Source: Nat Genet. 2023 Jun 8;55(6):1009–21. doi: 10.1038/s41588-023-01403-0 (PMC10260400; doi:10.1038/s41588-023-01403-0)
Supplement: Source Data Fig. 3 — Full-length blots (long exposure and very long exposure (to show protein ladder). [file 41588_2023_1403_MOESM11_ESM.pdf]

Source data for Figure 3a

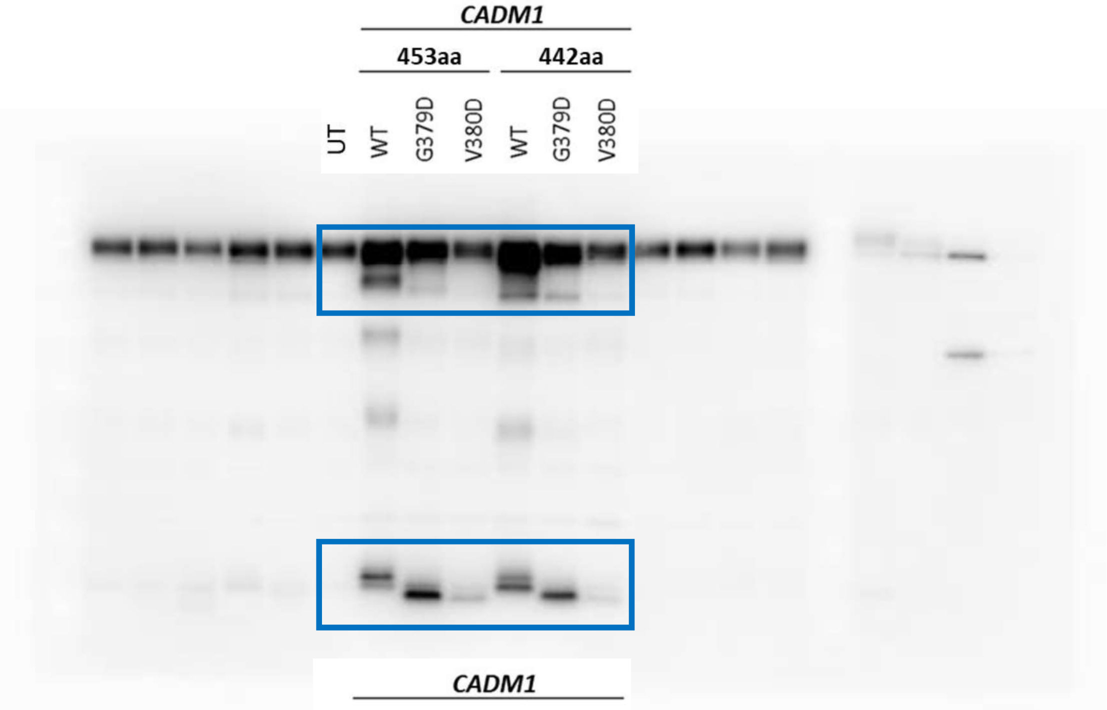

Long exposure

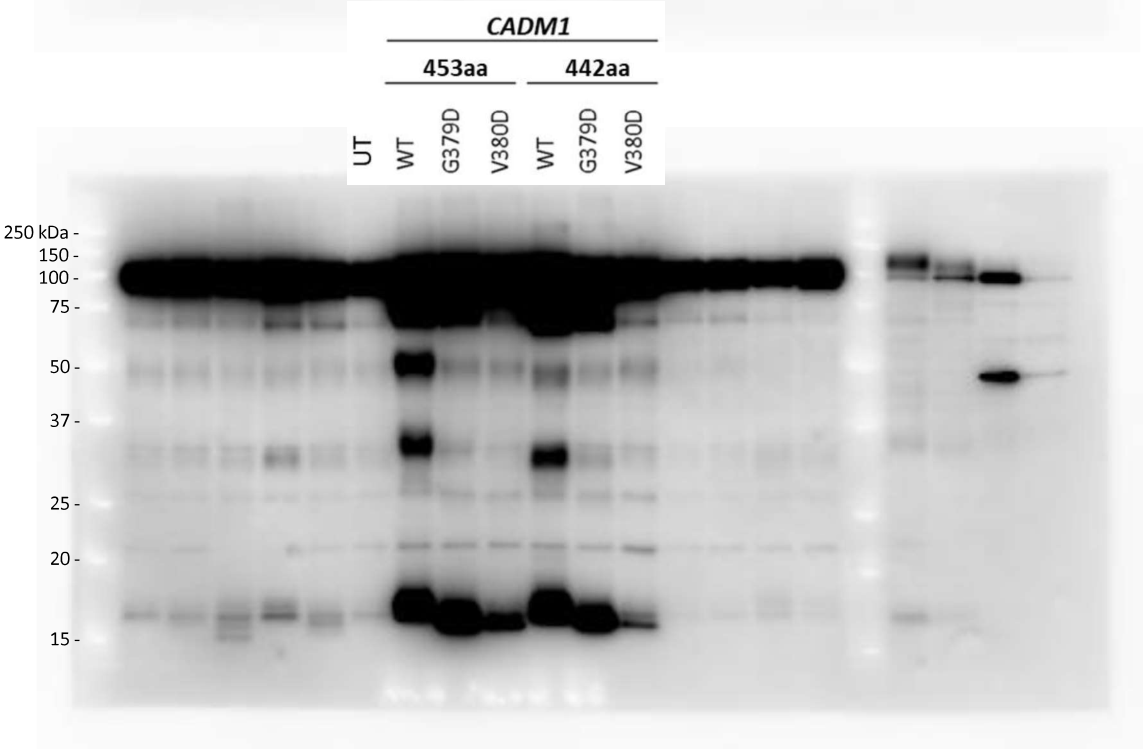

Very long exposure to show protein ladder

Cell lysates were immunoblotted with a custom-made anti-CADM1 C-terminal antibody.

Areas of Western Blot cropped and shown in **Figure 3a** are highlighted by the blue boxes respectively.
