# Supplementary figures and images for: Somatic mutations of CADM1 in aldosterone-producing adenomas and gap junction-dependent regulation of aldosterone production
Source: Nat Genet. 2023 Jun 8;55(6):1009–21. doi: 10.1038/s41588-023-01403-0 (PMC10260400; doi:10.1038/s41588-023-01403-0)

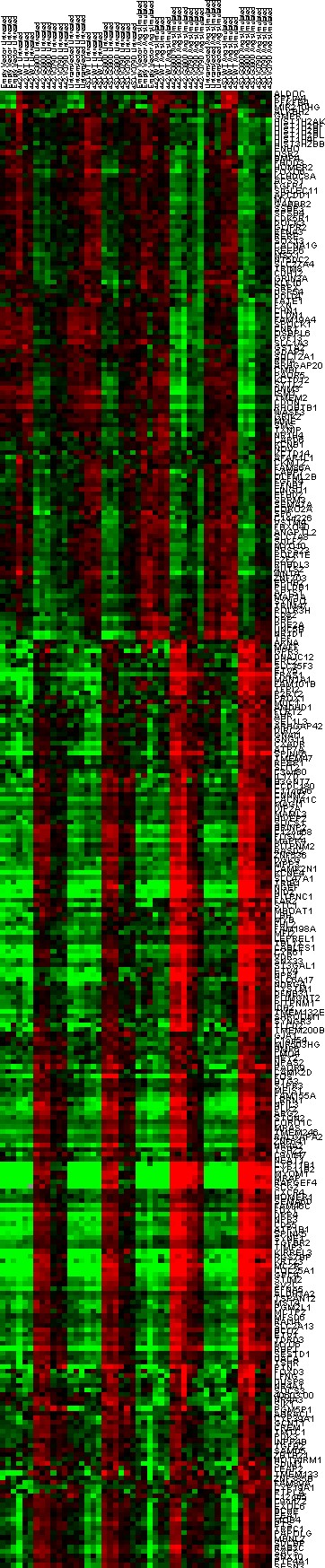

Supplement: Source Data Fig. 6 — Uncropped heatmap with gene names. [file 41588_2023_1403_MOESM14_ESM.jpg]
