# Supplementary material for: Somatic mutations of CADM1 in aldosterone-producing adenomas and gap junction-dependent regulation of aldosterone production
Source: Nat Genet. 2023 Jun 8;55(6):1009–21. doi: 10.1038/s41588-023-01403-0 (PMC10260400; doi:10.1038/s41588-023-01403-0)
Supplement: Source Data Extended Data Fig. 3 — Full-length gels of Extended Data Fig. 3a (along with GAPDH control). [file 41588_2023_1403_MOESM15_ESM.pdf]

Source data for Extended Data Figure 3a

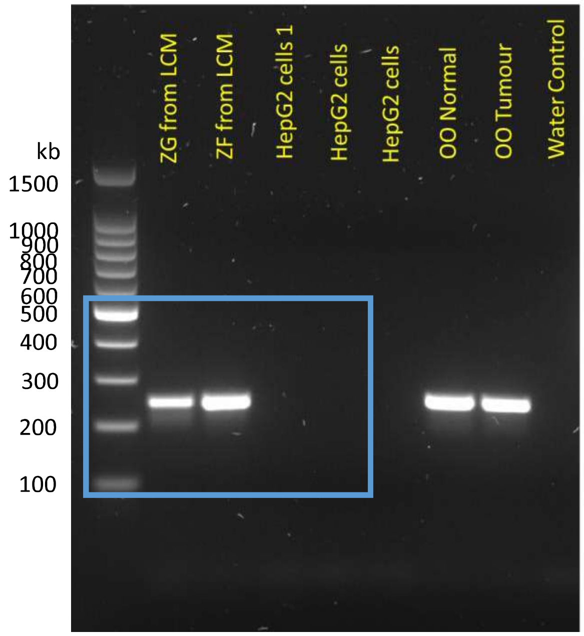

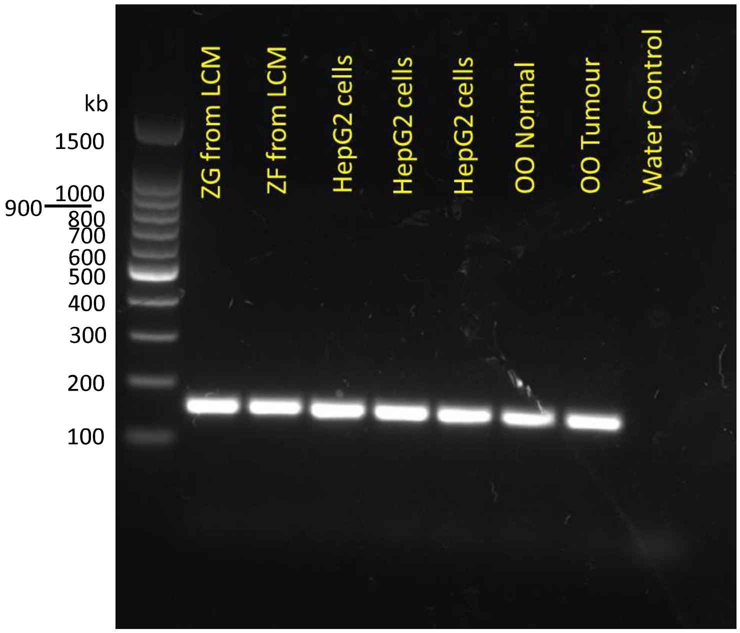

Gel showing **a.** GJA1 PCR products from cDNA reverse transcribed from ZG and ZF RNA samples procured using laser capture dissection (LCM). HepG2 cells were used as a negative control as they do not express GJA1. **b.** GAPDH PCR products from the same set of samples are shown to demonstrate equal loading of PCR products (gels run sequentially). Quantification of RNA expression (the 5.45-fold higher expression of *GJA1* in ZF LCM cDNA than ZG LCM samples shown in Extended Data Figure 3a) was performed using RT-PCR.

Areas of gel cropped and shown in **Extended Data Figure 3a** is highlighted by the blue box.
